# Supplementary figures and images for: Prevalence of the metabolic syndrome in African populations: A systematic review and meta-analysis
Source: PLoS One. 2023 Jul 27;18(7):e0289155. doi: 10.1371/journal.pone.0289155 (PMC10374159; doi:10.1371/journal.pone.0289155)

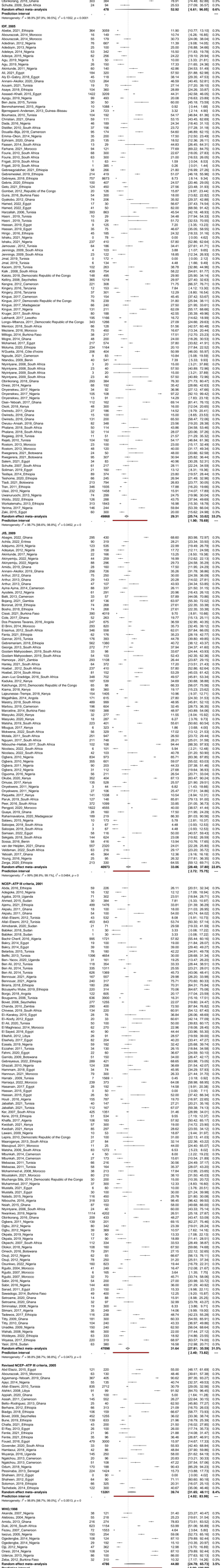

Supplement: S1 Fig — (PDF) [file pone.0289155.s001.pdf]

S1 Fig. Funnel chart for publications of the metabolic syndrome prevalence in Africa.

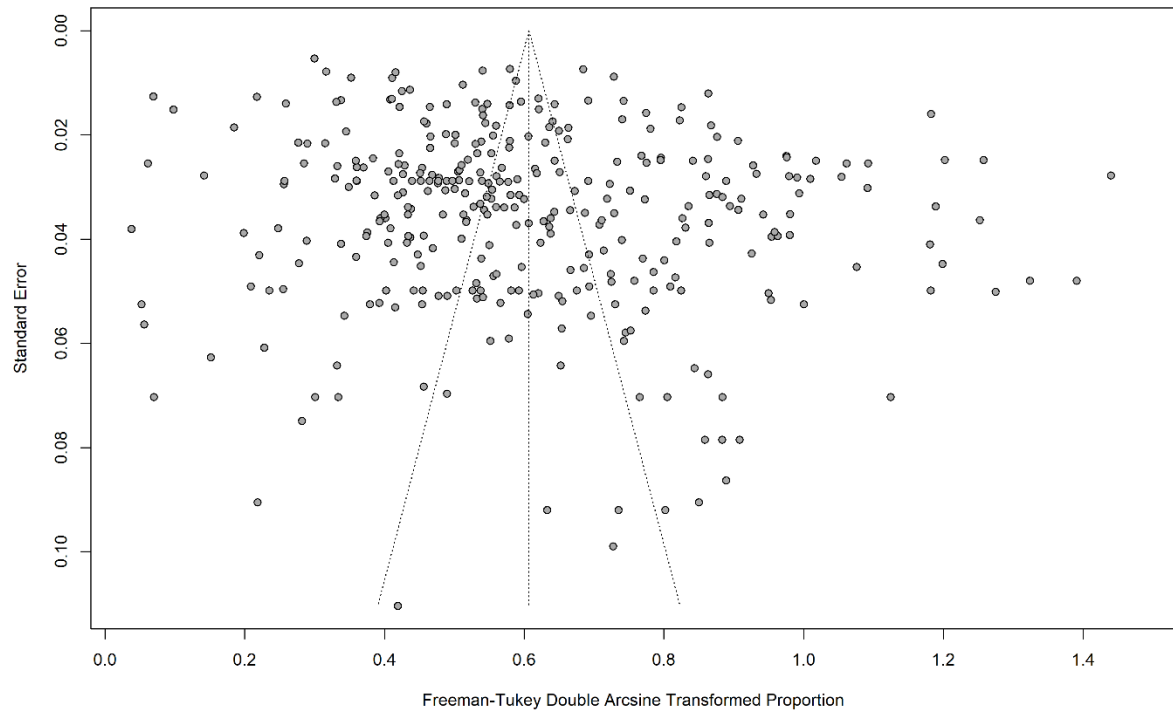

P Egger = < 0.0001

Supplement: S2 Fig — (PDF) [file pone.0289155.s002.pdf]
