# Supplementary material for: Prevalence of the metabolic syndrome in African populations: A systematic review and meta-analysis
Source: PLoS One. 2023 Jul 27;18(7):e0289155. doi: 10.1371/journal.pone.0289155 (PMC10374159; doi:10.1371/journal.pone.0289155)
Supplement: S2 Table — (PDF) [file pone.0289155.s004.pdf]

S2 Table: Search strategy in PubMed

Search	Virus
#1 Condition	Metabolic syndrome OR Metabolic Syndromes OR Syndrome, Metabolic OR Syndromes, Metabolic OR Metabolic Syndrome X OR Insulin Resistance Syndrome X OR Syndrome X, Metabolic OR Syndrome X, Insulin Resistance OR Metabolic X Syndrome OR Syndrome, Metabolic X OR X Syndrome, Metabolic OR Dysmetabolic Syndrome X OR Syndrome X, Dysmetabolic OR Reaven Syndrome X OR Syndrome X, Reaven OR Metabolic Cardiovascular Syndrome OR Cardiovascular Syndrome, Metabolic OR Cardiovascular Syndromes, Metabolic OR Syndrome, Metabolic Cardiovascular OR Cardiometabolic Syndrome OR Cardiometabolic Syndromes OR Syndrome, Cardiometabolic OR Syndromes, Cardiometabolic
#2 Context	Africa* OR Algeria OR Angola OR Benin OR Botswana OR "Burkina Faso" OR Burundi OR Cameroon OR "Canary Islands" OR "Cape Verde" OR "Central African Republic" OR Chad OR Comoros OR Congo OR "Democratic Republic of Congo" OR Djibouti OR Egypt OR "Equatorial Guinea" OR Eritrea OR Ethiopia OR Gabon OR Gambia OR Ghana OR Guinea OR "Guinea Bissau" OR "Ivory Coast" OR "Cote d'Ivoire" OR Jamahiriya OR Kenya OR Lesotho OR Liberia OR Libya OR Madagascar OR Malawi OR Mali OR Mauritania OR Mauritius OR Mayotte OR Morocco OR Mozambique OR Namibia OR Niger OR Nigeria OR Principe OR Reunion OR Rwanda OR "Sao Tome" OR Senegal OR Seychelles OR "Sierra Leone" OR Somalia OR "South Africa" OR “South Sudan” OR "St Helena" OR Sudan OR Swaziland OR Tanzania OR Togo OR Tunisia OR Uganda OR "Western Sahara" OR Zaire OR Zambia OR Zimbabwe OR "Central Africa" OR "Central African" OR "West Africa" OR "West African" OR "Western Africa" OR "Western African" OR "East Africa" OR "East African" OR "Eastern Africa" OR "Eastern African" OR "North Africa" OR "North African" OR "Northern Africa" OR "Northern African" OR "South African" OR "Southern Africa" OR "Southern African" OR "sub Saharan Africa" OR "sub Saharan African" OR "sub Saharan Africa" OR "sub Saharan African”
#3	#1 AND #2
#4	Limit #3 in English and French
