# Supplementary material for: Prevalence of the metabolic syndrome in African populations: A systematic review and meta-analysis
Source: PLoS One. 2023 Jul 27;18(7):e0289155. doi: 10.1371/journal.pone.0289155 (PMC10374159; doi:10.1371/journal.pone.0289155)
Supplement: S3 Table — (PDF) [file pone.0289155.s005.pdf]

S3 Table. Items for risk of bias assessment

**Hoy et al. tool for cross sectional studies**	**Yes (1)/No (0)**
**External validity**	
1. Was the study's target population a close representation of the national population in relation to metabolic syndrome prevalence or case fatality rate?	**1**
2. Was the sampling frame a true or close representation of the population?	**1**
3. Was some form of random selection used to select the sample, OR was a census undertaken?	**1**
4. Was the likelihood of non-response bias minimal?	**1**
**Internal validity**	
5. Were data collected directly from the subjects (as opposed to a proxy)?	**1**
6. Was an acceptable case definition used in the study?	**1**
7. Was the metabolic syndrome definition shown to have reliability and validity?	**1**
8. Was the same mode data collection used for all subjects?	**1**
9. Was the length of the length of the study period > 1 year?	**1**
10. Were the numerator(s) and denominator(s) for the prevalence or case fatality rate of metabolic syndrome appropriate?	**1**
Total score	**10**
**Interpretation of the risk of bias tool**<br>• 7-10: Low risk of bias<br>• 4-6: Moderate risk of bias<br>• 0-3: High risk of bias	
