# Supplementary material for: Prevalence of the metabolic syndrome in African populations: A systematic review and meta-analysis
Source: PLoS One. 2023 Jul 27;18(7):e0289155. doi: 10.1371/journal.pone.0289155 (PMC10374159; doi:10.1371/journal.pone.0289155)
Supplement: S4 Table — (PDF) [file pone.0289155.s006.pdf]

S4 Table. Characteristics of included studies

**Characteristics**	**Overall (345)**	**IDF, 2005 (115)**	**NCEP-ATP III criteria, 2001 (109)**	**JIS, 2009 (78)**	**Revised NCEP-ATP III criteria, 2005 (29)**	**WHO,1998 (12)**	**AACE 2003 (2)**
**Year of publication; range**	2004-2022	2006-2022	2004-2022	2010-2022	2008-2022	2004-2021	2009-2018
**Period of inclusion of participants; range**	1985-2021	1985-2021	1985-2020	2005-2020	2004-2021	1985-2017	2011-2012
**Study Design**							
Cross-sectional	318 (92.2)	102 (88.7)	100 (91.7)	75 (96.2)	27 (93.1)	12 (100)	2 (100)
Case control	20 (5.8)	9 (7.8)	9 (8.3)		2 (6.9)		
Cohort (Baseline data)	7 (2.0)	4 (3.5)		3 (3.9)			
**Sampling**							
Non probabilistic	284 (82.3)	97 (84.4)	92 (84.4)	60 (76.9)	23 (79.3)	10 (83.3)	2 (100)
Probabilistic	61 (17.7)	18 (15.7)	17 (15.6)	18 (23.1)	6 (20.7)	2 (16.7)	
**Setting**							
Hospital-based	248 (71.9)	79 (68.7)	87 (79.8)	49 (62.8)	23 (79.3)	9 (75.0)	1 (50.0)
Community-based	91 (26.4)	33 (28.7)	21 (19.3)	27 (34.6)	6 (20.7)	3 (25.0)	1 (50.0)
Hospital/community based	6 (1.7)	3 (2.6)	1 (0.9)	2 (2.6)			
**Number of sites**							
Monocenter	238 (69.0)	76 (66.1)	79 (72.5)	48 (61.5)	25 (86.2)	9 (75.0)	1 (50.0)
Multicenter	104 (30.1)	38 (33.0)	30 (27.5)	28 (35.9)	4 (13.8)	3 (25.0)	1 (50.0)
Nationally representative	3 (0.9)	1 (0.9)		2 (2.6)			
**Timing of samples collection**							
Prospetively	337 (97.7)	110 (95.7)	108 (99.1)	77 (98.7)	28 (96.6)	12 (100)	2 (100)
Retrospectively	8 (2.3)	5 (4.4)	1 (0.9)	1 (1.3)	1 (3.5)		
**Countries**							
Nigeria	66 (19.1)	20 (17.4)	21 (19.3)	15 (19.2)	2 (6.9)	8 (66.7)	
South Africa	49 (14.2)	18 (15.7)	6 (5.5)	22 (28.2)	1 (3.5)	1 (8.3)	1 (50.0)

Ethiopia	36 (10.4)	14 (12.2)	9 (8.3)	5 (6.4)	8 (27.6)		
Ghana	34 (9.9)	11 (9.6)	8 (7.3)	10 (12.8)	3 (10.3)	1 (8.3)	1 (50.0)
Egypt	31 (9.0)	11 (9.6)	14 (12.8)	1 (1.3)	5 (17.2)		
Tunisia	29 (8.4)	12 (10.4)	15 (13.8)	1 (1.3)	1 (3.5)		
Morocco	18 (5.2)	6 (5.2)	9 (8.3)	2 (2.6)	1 (3.5)		
Cameroon	13 (3.8)	4 (3.5)	2 (1.8)	3 (3.9)	3 (10.3)	1 (8.3)	
Kenya	12 (3.5)	1 (0.9)	2 (1.8)	9 (11.5)			
Algeria	9 (2.6)	1 (0.9)	7 (6.4)		1 (3.5)		
Burkina Faso	6 (1.7)	2 (1.7)	1 (0.9)	2 (2.6)		1 (8.3)	
Democratic Republic of the Congo	6 (1.7)	3 (2.6)	2 (1.8)	1 (1.3)			
Botswana	5 (1.5)	3 (2.6)	2 (1.8)				
Sudan	5 (1.5)		5 (4.6)				
Angola	3 (0.9)		1 (0.9)	2 (2.6)			
Seychelles	3 (0.9)	1 (0.9)	1 (0.9)		1 (3.5)		
Uganda	3 (0.9)		2 (1.8)		1 (3.5)		
Benin	2 (0.6)	1 (0.9)		1 (1.3)			
Libya	2 (0.6)		1 (0.9)		1 (3.5)		
Madagascar	2 (0.6)	1 (0.9)		1 (1.3)			
Tanzania	2 (0.6)	2 (1.7)					
Zambia	2 (0.6)		1 (0.9)	1 (1.3)			
Côte d'Ivoire	1 (0.3)	1 (0.9)					
Eritrea	1 (0.3)			1 (1.3)			
Gambia	1 (0.3)			1 (1.3)			
Guinea-Bissau	1 (0.3)	1 (0.9)					
Lesotho	1 (0.3)	1 (0.9)					
Republic of the Congo	1 (0.3)	1 (0.9)					
Senegal	1 (0.3)				1 (3.5)		
**WHO Region**							
Africa	260 (75.4)	86 (74.8)	65 (59.6)	74 (94.9)	21 (72.4)	12 (100)	2 (100)
Eastern Mediterranean	85 (24.6)	29 (25.2)	44 (40.4)	4 (5.1)	8 (27.6)		

**UNSD Region**							
West Africa	112 (32.5)	36 (31.3)	30 (27.5)	29 (37.2)	6 (20.7)	10 (83.3)	1 (50.0)
Northern Africa	94 (27.3)	30 (26.1)	51 (46.8)	4 (5.1)	9 (31.0)		
Eastern Africa	61 (17.7)	19 (16.5)	15 (13.8)	17 (21.8)	10 (34.5)		
Southern Africa	55 (15.9)	22 (19.1)	8 (7.3)	22 (28.2)	1 (3.5)	1 (8.3)	1 (50.0)
Central Africa	23 (6.7)	8 (7.0)	5 (4.6)	6 (7.7)	3 (10.3)	1 (8.3)	
**Country income level**							
Lower-middle income economies	225 (65.2)	72 (62.6)	80 (73.4)	45 (57.7)	17 (58.6)	10 (83.3)	1 (50.0)
Low-income economies	61 (17.7)	21 (18.3)	19 (17.4)	11 (14.1)	9 (31.0)	1 (8.3)	
Upper-middle-income economies	56 (16.2)	21 (18.3)	9 (8.3)	22 (28.2)	2 (6.9)	1 (8.3)	1 (50.0)
High-income economies	3 (0.9)	1 (0.9)	1 (0.9)		1 (3.5)		
**Age range**							
Adults: 18+ years	257 (74.5)	75 (65.2)	86 (78.9)	64 (82.1)	20 (69.0)	11 (91.7)	1 (50.0)
All ages	20 (5.8)	8 (7.0)	5 (4.6)	6 (7.7)	1 (3.5)		
Children : Birth-18 years	18 (5.2)	11 (9.6)	7 (6.4)				
Unclear/Not reported	50 (14.5)	21 (18.3)	11 (10.1)	8 (10.3)	8 (27.6)	1 (8.3)	1 (50.0)
**Population categories**							
Apparently healthy individuals	167 (48.4)	57 (49.6)	48 (44.0)	46 (59.0)	10 (34.5)	4 (33.3)	2 (100)
Type 2 diabetes patients	46 (13.3)	10 (8.7)	13 (11.9)	12 (15.4)	4 (13.8)	7 (58.3)	
HIV infected patients	32 (9.3)	15 (13.0)	5 (4.6)	8 (10.3)	4 (13.8)		
Hypertensive patients	18 (5.2)	6 (5.2)	7 (6.4)		4 (13.8)	1 (8.3)	
Psychiatric patients	13 (3.8)	5 (4.4)	5 (4.6)	1 (1.3)	2 (6.9)		

Patients with dermatological diseases	11 (3.2)	2 (1.7)	7 (6.4)	1 (1.3)	1 (3.5)		
Patients with obesity	11 (3.2)	6 (5.2)	5 (4.6)				
Postmenopausal women	8 (2.3)		6 (5.5)	2 (2.6)			
Patients with chronic diseases	6 (1.7)	1 (0.9)	1 (0.9)	2 (2.6)	2 (6.9)		
Patients with chronic kidney diseases	5 (1.5)		5 (4.6)				
Patients with coronary artery disease	5 (1.5)	4 (3.5)	1 (0.9)				
Patients with rheumatoid arthritis	5 (1.5)	3 (2.6)	1 (0.9)		1 (3.5)		
Patients with cardiovascular diseases	3 (0.9)		1 (0.9)	2 (2.6)			
Patients with Chronic musculoskeletal diseases	3 (0.9)	1 (0.9)	1 (0.9)	1 (1.3)			
Patients with respiratory tract diseases	3 (0.9)	2 (1.7)		1 (1.3)			
Patients with nonalcoholic fatty liver disease	2 (0.6)		2 (1.8)				
Patients with thyroid disease	2 (0.6)			1 (1.3)	1 (3.5)		
Patients with type 1 diabetes	2 (0.6)	2 (1.7)					

Exclusive narghile smokers	1 (0.3)	1 (0.9)					
patients with sickle cell disease	1 (0.3)		1 (0.9)				
Pregnant women	1 (0.3)			1 (1.3)			
**Risk of bias**							
Low risk of bias	174 (50.4)	63 (54.8)	46 (42.2)	47 (60.3)	14 (48.3)	3 (25.0)	1 (50.0)
Moderate risk of bias	171 (49.6)	52 (45.2)	63 (57.8)	31 (39.7)	15 (51.7)	9 (75.0)	1 (50.0)
