# Supplementary material for: Prevalence of the metabolic syndrome in African populations: A systematic review and meta-analysis
Source: PLoS One. 2023 Jul 27;18(7):e0289155. doi: 10.1371/journal.pone.0289155 (PMC10374159; doi:10.1371/journal.pone.0289155)
Supplement: S5 Table — (PDF) [file pone.0289155.s007.pdf]

Unique_ID	Author, Year of publication_Countries	Title	Study Design	Sampling	Sampling method	Setting	Number of sites	Timing of samples collection	Countries	WHO Region	UNSD Region	Country income level	Studyperiod	Age range	Population categories	MS definition
2	Abd Elaziz, 2015_Egypt	Prevalence of metabolic syndrome and cardiovascular risk factors among voluntary screened middle-aged and elderly Egyptians.	Cross-sectional	Non probabilistic	Consecutive sampling	Hospital-based	Monocenter	Prospetively	Egypt	Eastern Mediterranean	Northern Africa	Lower-middle income economies	Unclear/ Not reported	Adults: 18+ years	Apparently healthy individuals	Revised NCEP-ATP III, 2005
3	Abda, 2016_Ethiopia	Metabolic syndrome and associated factors among outpatients of Jimma University Teaching Hospital.	Cross-sectional	Non probabilistic	Consecutive sampling	Hospital-based	Monocenter	Prospetively	Ethiopia	Africa	Eastern Africa	Low-income economies	2014	Adults: 18+ years	Apparently healthy individuals	NCEP-ATP III, 2001
7	Abourazzak, 2015_Morocco	Does metabolic syndrome or its individual components affect pain and function in knee osteoarthritis women?	Cross-sectional	Non probabilistic	Consecutive sampling	Hospital-based	Monocenter	Prospetively	Morocco	Eastern Mediterranean	Northern Africa	Lower-middle income economies	Unclear/ Not reported	Adults: 18+ years	Patients with rheumatoid arthritis	Revised NCEP-ATP III, 2005
5	Abourazzak, 2014_Morocco	Prevalence of metabolic syndrome in patients with rheumatoid arthritis in Morocco: a cross-sectional study of 179 cases.	Cross-sectional	Non probabilistic	Consecutive sampling	Hospital-based	Monocenter	Prospetively	Morocco	Eastern Mediterranean	Northern Africa	Lower-middle income economies	Jul/2011-Dec/2012	Adults: 18+ years	Apparently healthy individuals	IDF, 2005
6	Abourazzak, 2014_Morocco	Prevalence of metabolic syndrome in patients with rheumatoid arthritis in Morocco: a cross-sectional study of 179 cases.	Cross-sectional	Non probabilistic	Consecutive sampling	Hospital-based	Monocenter	Prospetively	Morocco	Eastern Mediterranean	Northern Africa	Lower-middle income economies	Jul/2011-Dec/2012	Adults: 18+ years	Patients with rheumatoid arthritis	IDF, 2005
9	Adediran, 2012_Nigeria	Impact of urbanization and gender on frequency of metabolic syndrome among native Abuja settlers in Nigeria.	Cross-sectional	Non probabilistic	Consecutive sampling	Community-based	Multicenter	Prospetively	Nigeria	Africa	West Africa	Lower-middle income economies	Unclear/ Not reported	Adults: 18+ years	Apparently healthy individuals	IDF, 2005
10	Adedoyin, 2013_Nigeria	Relationship between socioeconomic status and metabolic syndrome among Nigerian adults.	Cross-sectional	Non probabilistic	Consecutive sampling	Community-based	Monocenter	Prospetively	Nigeria	Africa	West Africa	Lower-middle income economies	Unclear/ Not reported	Adults: 18+ years	Apparently healthy individuals	IDF, 2005
11	Adegoke, 2010_Nigeria	Prevalence of metabolic syndrome in a rural community in Nigeria.	Cross-sectional	Non probabilistic	Consecutive sampling	Community-based	Multicenter	Prospetively	Nigeria	Africa	West Africa	Lower-middle income economies	Unclear/ Not reported	Adults: 18+ years	Apparently healthy individuals	NCEP-ATP III, 2001
12	Adejumo, 2019_Nigeria	Anthropometric parameter that best predict metabolic syndrome in South west Nigeria.	Cross-sectional	Non probabilistic	Consecutive sampling	Community-based	Multicenter	Prospetively	Nigeria	Africa	West Africa	Lower-middle income economies	Unclear/ Not reported	Adults: 18+ years	Apparently healthy individuals	JIS, 2009
13	Adeleye, 2014_Nigeria	Metabolic Syndrome in Patients attending the Staff Clinic of a Nigerian Tertiary Hospital.	Cross-sectional	Non probabilistic	Consecutive sampling	Hospital-based	Monocenter	Prospetively	Nigeria	Africa	West Africa	Lower-middle income economies	Unclear/ Not reported	Adults: 18+ years	Apparently healthy individuals	IDF, 2005
14	Adeoye, 2015_Nigeria	Excess Metabolic Syndrome Risks Among Women Health Workers Compared With Men.	Cross-sectional	Non probabilistic	Consecutive sampling	Hospital-based	Monocenter	Prospetively	Nigeria	Africa	West Africa	Lower-middle income economies	Unclear/ Not reported	Unclear/Not reported	Apparently healthy individuals	IDF, 2005
15	Adeyeye, 2012_Nigeria	Understanding asthma and the metabolic syndrome - a Nigerian report.	Cross-sectional	Non probabilistic	Consecutive sampling	Hospital-based	Monocenter	Prospetively	Nigeria	Africa	West Africa	Lower-middle income economies	Unclear/ Not reported	All ages	Patients with respiratory tract diseases	JIS, 2009
16	Agaba, 2019_Uganda	Prevalence and Associated Factors of Metabolic Syndrome among Patients with Severe Mental Illness Attending a Tertiary Hospital in Southwest Uganda.	Cross-sectional	Non probabilistic	Consecutive sampling	Hospital-based	Monocenter	Prospetively	Uganda	Africa	Eastern Africa	Low-income economies	Oct/2018-Mar/2019	Adults: 18+ years	Psychiatric patients	NCEP-ATP III, 2001
17	Agu, 2019_Nigeria	Prevalence and associated risk factors of peripheral artery disease in virologically suppressed HIV-infected individuals on antiretroviral therapy in Kwara state, Nigeria: a cross sectional study.	Cross-sectional	Non probabilistic	Consecutive sampling	Hospital-based	Monocenter	Prospetively	Nigeria	Africa	West Africa	Lower-middle income economies	2018	Adults: 18+ years	Apparently healthy individuals	IDF, 2005
18	Agu, 2019_Nigeria	Prevalence and associated risk factors of peripheral artery disease in virologically suppressed HIV-infected individuals on antiretroviral therapy in Kwara state, Nigeria: a cross sectional study.	Cross-sectional	Non probabilistic	Consecutive sampling	Hospital-based	Monocenter	Prospetively	Nigeria	Africa	West Africa	Lower-middle income economies	2018	Adults: 18+ years	HIV infected patients	IDF, 2005
19	Agyemang-Yeboah, 2019_Ghana	Evaluation of Metabolic Syndrome and Its Associated Risk Factors in Type 2 Diabetes: A Descriptive Cross-Sectional Study at the Komfo Anokye Teaching Hospital, Kumasi, Ghana.	Cross-sectional	Non probabilistic	Consecutive sampling	Hospital-based	Monocenter	Prospetively	Ghana	Africa	West Africa	Lower-middle income economies	Unclear/ Not reported	Unclear/Not reported	Type 2 diabetes patients	Revised NCEP-ATP III, 2005
20	Ahmed, 2015_Sudan	Metabolic Syndrome among Undergraduate Students Attending Medical Clinics for Obligatory Medical Screening.	Cross-sectional	Non probabilistic	Consecutive sampling	Hospital-based	Multicenter	Prospetively	Sudan	Eastern Mediterranean	Northern Africa	Low-income economies	2013	Adults: 18+ years	Apparently healthy individuals	NCEP-ATP III, 2001
21	Ajayi, 2014_Nigeria	Metabolic syndrome: prevalence and association with electrocardiographic abnormalities in Nigerian hypertensive patients.	Cross-sectional	Non probabilistic	Consecutive sampling	Hospital-based	Monocenter	Prospetively	Nigeria	Africa	West Africa	Lower-middle income economies	2013	Adults: 18+ years	Hypertensive patients	Revised NCEP-ATP III, 2005
22	Ajemu, 2021_Ethiopia	Magnitude, components and predictors of metabolic syndrome in Northern Ethiopia: Evidences from regional NCDs STEPS survey, 2016.	Cross-sectional	Non probabilistic	Consecutive sampling	Community-based	Monocenter	Prospetively	Ethiopia	Africa	Eastern Africa	Low-income economies	2016	Adults: 18+ years	Apparently healthy individuals	NCEP-ATP III, 2001
23	Akande, 2007_Nigeria	Serum uric acid level as an independent component of the metabolic syndrome in type 2 diabetic blacks.	Cross-sectional	Non probabilistic	Consecutive sampling	Hospital-based	Monocenter	Prospetively	Nigeria	Africa	West Africa	Lower-middle income economies	Unclear/ Not reported	Adults: 18+ years	Type 2 diabetes patients	WHO,1998
24	Akintunde, 2011_Nigeria	Metabolic syndrome: comparison of occurrence using three definitions in hypertensive patients.	Cross-sectional	Non probabilistic	Consecutive sampling	Hospital-based	Monocenter	Prospetively	Nigeria	Africa	West Africa	Lower-middle income economies	Unclear/ Not reported	Adults: 18+ years	Hypertensive patients	IDF, 2005
25	Akintunde, 2017_Nigeria	Metabolic syndrome and occupation: Any association? Prevalence among auto technicians and school teachers in South West Nigeria.	Cross-sectional	Non probabilistic	Consecutive sampling	Community-based	Multicenter	Prospetively	Nigeria	Africa	West Africa	Lower-middle income economies	2016	Adults: 18+ years	Apparently healthy individuals	JIS, 2009
28	Akpalu, 2011_Ghana	The metabolic syndrome among patients with cardiovascular disease in Accra, Ghana.	Case control	Non probabilistic	Consecutive sampling	Hospital-based	Monocenter	Prospetively	Ghana	Africa	West Africa	Lower-middle income economies	Unclear/ Not reported	Adults: 18+ years	Apparently healthy individuals	NCEP-ATP III, 2001
29	Akpalu, 2011_Ghana	The metabolic syndrome among patients with cardiovascular disease in Accra, Ghana.	Case control	Non probabilistic	Consecutive sampling	Hospital-based	Monocenter	Prospetively	Ghana	Africa	West Africa	Lower-middle income economies	Unclear/ Not reported	Adults: 18+ years	Patients with cardiovascular diseases	NCEP-ATP III, 2001
30	Alebiosu, 2004_Nigeria	Metabolic syndrome in subjects with type-2 diabetes mellitus.	Cross-sectional	Non probabilistic	Consecutive sampling	Hospital-based	Monocenter	Prospetively	Nigeria	Africa	West Africa	Lower-middle income economies	Sep/1999-Aug/2001	Adults: 18+ years	Type 2 diabetes patients	WHO,1998
31	Ali, 2021_Egypt	Prevalence and Predictors of Metabolic Syndrome among Patients with Bronchial Asthma: A Cross Sectional Study.	Cross-sectional	Non probabilistic	Consecutive sampling	Hospital-based	Monocenter	Prospetively	Egypt	Eastern Mediterranean	Northern Africa	Lower-middle income economies	Unclear/ Not reported	Unclear/Not reported	Patients with respiratory tract diseases	IDF, 2005
33	Allal-Elasmi, 2012_Tunisia	Prehypertension among adults in Great Tunis region (Tunisia): A population-based study.	Cross-sectional	Non probabilistic	Consecutive sampling	Community-based	Monocenter	Prospetively	Tunisia	Eastern Mediterranean	Northern Africa	Lower-middle income economies	2004-2005	Adults: 18+ years	Apparently healthy individuals	NCEP-ATP III, 2001
34	Allal-Elasmi, 2012_Tunisia	Prehypertension among adults in Great Tunis region (Tunisia): A population-based study.	Cross-sectional	Non probabilistic	Consecutive sampling	Community-based	Monocenter	Prospetively	Tunisia	Eastern Mediterranean	Northern Africa	Lower-middle income economies	2004-2005	Adults: 18+ years	Hypertensive patients	NCEP-ATP III, 2001
32	Allal-Elasmi, 2010_Tunisia	The metabolic syndrome: prevalence, main characteristics and association with socio-economic status in adults living in Great Tunis.	Cross-sectional	Non probabilistic	Consecutive sampling	Community-based	Monocenter	Prospetively	Tunisia	Eastern Mediterranean	Northern Africa	Lower-middle income economies	2004-2005	Adults: 18+ years	Apparently healthy individuals	Revised NCEP-ATP III, 2005
35	Almobarak, 2020_Sudan	The prevalence of diabetes and metabolic syndrome and associated risk factors in Sudanese individuals with gallstones: a cross sectional survey.	Cross-sectional	Non probabilistic	Consecutive sampling	Hospital-based	Monocenter	Prospetively	Sudan	Eastern Mediterranean	Northern Africa	Low-income economies	2018	Adults: 18+ years	Patients with dermatological diseases	NCEP-ATP III, 2001
36	Alshkri, 2008_Libya	Metabolic Syndrome among Type-2 Diabetic Patients in Benghazi-Libya: A pilot study.	Cross-sectional	Non probabilistic	Consecutive sampling	Hospital-based	Monocenter	Prospetively	Libya	Eastern Mediterranean	Northern Africa	Upper-middle-income economies	2007	Adults: 18+ years	Type 2 diabetes patients	Revised NCEP-ATP III, 2005
37	Aly El-Gabry, 2018_Egypt	Antipsychotic Polypharmacy and Its Relation to Metabolic Syndrome in Patients With Schizophrenia: An Egyptian Study.	Cross-sectional	Non probabilistic	Consecutive sampling	Hospital-based	Multicenter	Prospetively	Egypt	Eastern Mediterranean	Northern Africa	Lower-middle income economies	Jun/2011-Jan/2013	Adults: 18+ years	Psychiatric patients	IDF, 2005
39	Amidu, 2019_Ghana	Comparative Abilities of Fasting Plasma Glucose and Haemoglobin A1c in Predicting Metabolic Syndrome among Apparently Healthy Normoglycemic Ghanaian Adults.	Cross-sectional	Non probabilistic	Consecutive sampling	Community-based	Monocenter	Prospetively	Ghana	Africa	West Africa	Lower-middle income economies	Sep/2017-Jan/2018	Adults: 18+ years	Apparently healthy individuals	JIS, 2009
38	Amidu, 2013_Ghana	Association between metabolic syndrome and sexual dysfunction among men with clinically diagnosed diabetes.	Cross-sectional	Non probabilistic	Consecutive sampling	Hospital-based	Multicenter	Prospetively	Ghana	Africa	West Africa	Lower-middle income economies	Nov/2010-Mar/2011	Adults: 18+ years	Type 2 diabetes patients	WHO,1998
41	Annani-Akollor, 2020_Ghana	Population-derived cut-off for HbA1c could enhance the identification of metabolic syndrome among non-diabetic population.	Cross-sectional	Non probabilistic	Consecutive sampling	Hospital-based	Monocenter	Prospetively	Ghana	Africa	West Africa	Lower-middle income economies	Aug/2015-Nov/2016	Adults: 18+ years	Apparently healthy individuals	IDF, 2005
40	Annani-Akollor, 2019_Ghana	Prevalence of metabolic syndrome and the comparison of fasting plasma glucose and HbA1c as the glycemic criterion for MetS definition in non-diabetic population in Ghana.	Cross-sectional	Non probabilistic	Consecutive sampling	Hospital-based	Monocenter	Prospetively	Ghana	Africa	West Africa	Lower-middle income economies	Unclear/ Not reported	Adults: 18+ years	Apparently healthy individuals	JIS, 2009
42	Aouam, 2021_Tunisia	[Metabolic syndrome among people with HIV in central Tunisia: Prevalence and associated factors].	Cross-sectional	Non probabilistic	Consecutive sampling	Hospital-based	Monocenter	Prospetively	Tunisia	Eastern Mediterranean	Northern Africa	Lower-middle income economies	Unclear/ Not reported	Unclear/Not reported	HIV infected patients	IDF, 2005
43	Appiah, 2020_Ghana	Prevalence and lifestyle-associated risk factors of metabolic syndrome among commercial motor vehicle drivers in a metropolitan city in Ghana.	Cross-sectional	Non probabilistic	Convenience sampling	Community-based	Monocenter	Prospetively	Ghana	Africa	West Africa	Lower-middle income economies	Unclear/ Not reported	Adults: 18+ years	Apparently healthy individuals	Revised NCEP-ATP III, 2005
44	Arthur, 2012_Ghana	Prediction of metabolic syndrome among postmenopausal Ghanaian women using obesity and atherogenic markers.	Cross-sectional	Non probabilistic	Consecutive sampling	Hospital-based	Multicenter	Prospetively	Ghana	Africa	West Africa	Lower-middle income economies	2011	Adults: 18+ years	Postmenopausal women	JIS, 2009
45	Arthur, 2013_Ghana	The prevalence of metabolic syndrome and its predominant components among pre-and postmenopausal Ghanaian women.	Cross-sectional	Non probabilistic	Consecutive sampling	Hospital-based	Multicenter	Prospetively	Ghana	Africa	West Africa	Lower-middle income economies	2011	Unclear/Not reported	Apparently healthy individuals	JIS, 2009
46	Arthur, 2013_Ghana	The prevalence of metabolic syndrome and its predominant components among pre-and postmenopausal Ghanaian women.	Cross-sectional	Non probabilistic	Consecutive sampling	Hospital-based	Multicenter	Prospetively	Ghana	Africa	West Africa	Lower-middle income economies	2011	Unclear/Not reported	Postmenopausal women	JIS, 2009
47	Asaye, 2018_Ethiopia	Metabolic syndrome and associated factors among psychiatric patients in Jimma University Specialized Hospital, South West Ethiopia.	Cross-sectional	Non probabilistic	Consecutive sampling	Hospital-based	Monocenter	Prospetively	Ethiopia	Africa	Eastern Africa	Low-income economies	2015	Adults: 18+ years	Psychiatric patients	IDF, 2005
48	Assaad-Khalil, 2015_Egypt	Optimal waist circumference cutoff points for the determination of abdominal obesity and detection of cardiovascular risk factors among adult Egyptian population.	Cross-sectional	Probabilistic	Multistage sampling	Community-based	Multicenter	Prospetively	Egypt	Eastern Mediterranean	Northern Africa	Lower-middle income economies	2009-2011	Adults: 18+ years	Apparently healthy individuals	IDF, 2005
49	Assah, 2011_Cameroon	Urbanization, physical activity, and metabolic health in sub-Saharan Africa.	Cross-sectional	Non probabilistic	Consecutive sampling	Community-based	Multicenter	Prospetively	Cameroon	Africa	Central Africa	Lower-middle income economies	Unclear/ Not reported	Adults: 18+ years	Apparently healthy individuals	Revised NCEP-ATP III, 2005
50	Ayina Ayina, 2014_Cameroon	Osteoprotegerin is not a determinant of metabolic syndrome in sub-Saharan Africans after age adjustment.	Cross-sectional	Non probabilistic	Consecutive sampling	Community-based	Multicenter	Prospetively	Cameroon	Africa	Central Africa	Lower-middle income economies	2010	Adults: 18+ years	Apparently healthy individuals	JIS, 2009
51	Ayodele, 2012_Nigeria	Prevalence and clinical correlates of metabolic syndrome in Nigerians living with human immunodeficiency virus/acquired immunodeficiency syndrome.	Cross-sectional	Non probabilistic	Consecutive sampling	Hospital-based	Monocenter	Prospetively	Nigeria	Africa	West Africa	Lower-middle income economies	Unclear/ Not reported	Adults: 18+ years	HIV infected patients	JIS, 2009
52	Ayogu, 2019_Nigeria	Components and Risk Factors of Metabolic Syndrome among Rural Nigerian Workers.	Cross-sectional	Non probabilistic	Consecutive sampling	Community-based	Monocenter	Prospetively	Nigeria	Africa	West Africa	Lower-middle income economies	Unclear/ Not reported	Adults: 18+ years	Apparently healthy individuals	IDF, 2005
53	Babiker, 2018_Sudan	Risk factors of metabolic syndrome among adult Sudanese sickle cell anemia patients.	Cross-sectional	Non probabilistic	Consecutive sampling	Hospital-based	Monocenter	Prospetively	Sudan	Eastern Mediterranean	Northern Africa	Low-income economies	Aug/2017-Nov/2017	Adults: 18+ years	Apparently healthy individuals	NCEP-ATP III, 2001
54	Babiker, 2018_Sudan	Risk factors of metabolic syndrome among adult Sudanese sickle cell anemia patients.	Cross-sectional	Non probabilistic	Consecutive sampling	Hospital-based	Monocenter	Prospetively	Sudan	Eastern Mediterranean	Northern Africa	Low-income economies	Aug/2017-Nov/2017	Adults: 18+ years	patients with sickle cell disease	NCEP-ATP III, 2001
55	Bachir Cherif, 2016_Algeria	[The characteristics of arterial hypertension in postmenopausal women in the area of Blida (Algeria)].	Cross-sectional	Non probabilistic	Consecutive sampling	Hospital-based	Monocenter	Prospetively	Algeria	Africa	Northern Africa	Lower-middle income economies	Jun/2013-Jun/2015	Adults: 18+ years	Hypertensive patients	NCEP-ATP III, 2001
56	Bakry, 2014_Egypt	Androgenetic alopecia, metabolic syndrome, and insulin resistance: Is there any association? A case-control study.	Case control	Non probabilistic	Consecutive sampling	Hospital-based	Multicenter	Prospetively	Egypt	Eastern Mediterranean	Northern Africa	Lower-middle income economies	Mar/2011-Mar/2012	Adults: 18+ years	Apparently healthy individuals	NCEP-ATP III, 2001
57	Bakry, 2014_Egypt	Androgenetic alopecia, metabolic syndrome, and insulin resistance: Is there any association? A case-control study.	Case control	Non probabilistic	Consecutive sampling	Hospital-based	Multicenter	Prospetively	Egypt	Eastern Mediterranean	Northern Africa	Lower-middle income economies	Mar/2011-Mar/2012	Adults: 18+ years	Patients with dermatological diseases	NCEP-ATP III, 2001
58	Balti, 2013_Cameroon	Metabolic syndrome and fatal outcomes in the post-stroke event: a 5-year cohort study in Cameroon.	Cross-sectional	Non probabilistic	Consecutive sampling	Hospital-based	Monocenter	Prospetively	Cameroon	Africa	Central Africa	Lower-middle income economies	2006	Unclear/Not reported	Patients with cardiovascular diseases	JIS, 2009
59	Belarbia, 2015_Tunisia	Metabolic syndrome and chronic kidney disease.	Cross-sectional	Non probabilistic	Consecutive sampling	Hospital-based	Monocenter	Prospetively	Tunisia	Eastern Mediterranean	Northern Africa	Lower-middle income economies	2009	All ages	Patients with chronic kidney diseases	NCEP-ATP III, 2001
60	Belfki, 2013_Tunisia	Prevalence and determinants of the metabolic syndrome among Tunisian adults: results of the Transition and Health Impact in North Africa (TAHINA) project.	Cross-sectional	Non probabilistic	Consecutive sampling	Community-based	Multicenter	Prospetively	Tunisia	Eastern Mediterranean	Northern Africa	Lower-middle income economies	Apr/2004-Sep/2005	Adults: 18+ years	Apparently healthy individuals	NCEP-ATP III, 2001
61	Bello-Rodriguez, 2013_Ghana	The relationship between metabolic syndrome and target organ damage in Ghanaian with stage-2 hypertension.	Cross-sectional	Non probabilistic	Consecutive sampling	Hospital-based	Monocenter	Prospetively	Ghana	Africa	West Africa	Lower-middle income economies	Feb/2009-Jan/2010	Adults: 18+ years	Hypertensive patients	Revised NCEP-ATP III, 2005
63	Ben Ali, 2014_Tunisia	Menopause and metabolic syndrome in tunisian women.	Cross-sectional	Probabilistic	Stratified sampling	Community-based	Multicenter	Prospetively	Tunisia	Eastern Mediterranean	Northern Africa	Lower-middle income economies	Apr/2004-Sep/2005	Adults: 18+ years	Apparently healthy individuals	NCEP-ATP III, 2001
64	Ben Ali, 2014_Tunisia	Menopause and metabolic syndrome in tunisian women.	Cross-sectional	Probabilistic	Stratified sampling	Community-based	Multicenter	Prospetively	Tunisia	Eastern Mediterranean	Northern Africa	Lower-middle income economies	Apr/2004-Sep/2005	Adults: 18+ years	Postmenopausal women	NCEP-ATP III, 2001
62	Ben Ali, 2012_Tunisia	Adiponectin and metabolic syndrome in a Tunisian population.	Cross-sectional	Non probabilistic	Consecutive sampling	Hospital-based	Monocenter	Prospetively	Tunisia	Eastern Mediterranean	Northern Africa	Lower-middle income economies	Jan/2005-Jul/2007	Adults: 18+ years	Apparently healthy individuals	NCEP-ATP III, 2001
65	Ben Jazia, 2015_Tunisia	[Prevalence of metabolic syndrome in a population of apneic patients: a prospective study with cardiovascular risk estimation].	Cross-sectional	Non probabilistic	Consecutive sampling	Hospital-based	Monocenter	Prospetively	Tunisia	Eastern Mediterranean	Northern Africa	Lower-middle income economies	2013	Adults: 18+ years	Patients with respiratory tract diseases	IDF, 2005
66	Benmohammed, 2015_Algeria	Anthropometric markers for detection of the metabolic syndrome in adolescents.	Cross-sectional	Probabilistic	Multistage sampling	Community-based	Multicenter	Prospetively	Algeria	Africa	Northern Africa	Lower-middle income economies	Mar/200-Apr/2007	Children : Birth-18 years	Apparently healthy individuals	IDF, 2005
69	Ben-Yacov, 2020_Uganda	Prevalence and sex-specific patterns of metabolic syndrome in rural Uganda.	Cross-sectional	Probabilistic	Simple random sampling	Community-based	Multicenter	Prospetively	Uganda	Africa	Eastern Africa	Low-income economies	Unclear/ Not reported	Adults: 18+ years	Apparently healthy individuals	NCEP-ATP III, 2001
70	Berhane, 2012_Ethiopia	Prevalence of lipodystrophy and metabolic syndrome among HIV positive individuals on Highly Active Anti-Retroviral treatment in Jimma, South West Ethiopia.	Cross-sectional	Probabilistic	Stratified sampling	Hospital-based	Monocenter	Prospetively	Ethiopia	Africa	Eastern Africa	Low-income economies	Sep/2010-Dec/2010	Adults: 18+ years	HIV infected patients	Revised NCEP-ATP III, 2005
71	Biadgo, 2018_Ethiopia	The Prevalence of Metabolic Syndrome and Its Components among Type 2 Diabetes Mellitus Patients at a Tertiary Hospital, Northwest Ethiopia.	Cross-sectional	Probabilistic	Systematic sampling	Hospital-based	Monocenter	Prospetively	Ethiopia	Africa	Eastern Africa	Low-income economies	Jun/2015-Jul/2015	Unclear/Not reported	Type 2 diabetes patients	Revised NCEP-ATP III, 2005
72	Birarra, 2018_Ethiopia	Metabolic syndrome among type 2 diabetic patients in Ethiopia: a cross-sectional study.	Cross-sectional	Non probabilistic	Consecutive sampling	Hospital-based	Monocenter	Prospetively	Ethiopia	Africa	Eastern Africa	Low-income economies	Mar/2017-May/2017	Adults: 18+ years	Type 2 diabetes patients	NCEP-ATP III, 2001
73	Bizuayehu Wube, 2019_Ethiopia	A Comparative Prevalence Of Metabolic Syndrome Among Type 2 Diabetes Mellitus Patients In Hawassa University Comprehensive Specialized Hospital Using Four Different Diagnostic Criteria.	Cross-sectional	Probabilistic	Simple random sampling	Hospital-based	Monocenter	Prospetively	Ethiopia	Africa	Eastern Africa	Low-income economies	Feb/2017-May/2017	Adults: 18+ years	Type 2 diabetes patients	NCEP-ATP III, 2001
74	Bjerregaard-Andersen, 2013_Guinea-Bissau	Risk of metabolic syndrome and diabetes among young twins and singletons in Guinea-Bissau.	Cross-sectional	Non probabilistic	Consecutive sampling	Community-based	Multicenter	Prospetively	Guinea-Bissau	Africa	West Africa	Low-income economies	Oct/2009-Aug/2011	All ages	Apparently healthy individuals	IDF, 2005
76	Borgo, 2018_Angola	Lipid disorders among Black Africans non-users of lipid-lowering medication.	Cross-sectional	Non probabilistic	Consecutive sampling	Community-based	Monocenter	Prospetively	Angola	Africa	Central Africa	Lower-middle income economies	2009-2010	Adults: 18+ years	Apparently healthy individuals	NCEP-ATP III, 2001
77	Boronat, 2018_Ethiopia	Prevalence of the metabolic syndrome in the island of Gran Canaria: comparison of three major diagnostic proposals.	Cross-sectional	Non probabilistic	Consecutive sampling	Hospital-based	Monocenter	Prospetively	Ethiopia	Africa	Eastern Africa	Low-income economies	2008	Adults: 18+ years	HIV infected patients	JIS, 2009
78	Bosho, 2018_Ethiopia	Prevalence and predictors of metabolic syndrome among people living with human immunodeficiency virus (PLWHIV).	Cross-sectional	Non probabilistic	Consecutive sampling	Hospital-based	Monocenter	Prospetively	Ethiopia	Africa	Eastern Africa	Low-income economies	2016	Adults: 18+ years	HIV infected patients	JIS, 2009
79	Bouguerra, 2006_Tunisia	Prevalence of metabolic abnormalities in the Tunisian adults: a population based study.	Cross-sectional	Non probabilistic	Consecutive sampling	Community-based	Multicenter	Prospetively	Tunisia	Eastern Mediterranean	Northern Africa	Lower-middle income economies	1996-1997	Adults: 18+ years	Apparently healthy individuals	NCEP-ATP III, 2001
80	Boumaiza, 2010_Tunisia	Apolipoprotein B and non-high-density lipoprotein cholesterol are better risk markers for coronary artery disease than low-density lipoprotein cholesterol in hypertriglyceridemic metabolic syndrome patients.	Cross-sectional	Non probabilistic	Consecutive sampling	Hospital-based	Monocenter	Prospetively	Tunisia	Eastern Mediterranean	Northern Africa	Lower-middle income economies	Unclear/ Not reported	Unclear/Not reported	Patients with coronary artery disease	IDF, 2005

82	Bovet, 2009_Seychelles	Divergent fifteen-year trends in traditional and cardiometabolic risk factors of cardiovascular diseases in the Seychelles.	Cross-sectional	Non probabilistic	Consecutive sampling	Community-based	Multicenter	Retrospectively	Seychelles	Africa	Eastern Africa	High-income economies	2004	Adults: 18+ years	Apparently healthy individuals	Revised NCEP-ATP III, 2005
81	Bovet, 2006_Seychelles	Prevalence of cardiovascular risk factors in a middle-income country and estimated cost of a treatment strategy.	Cross-sectional	Non probabilistic	Consecutive sampling	Community-based	Multicenter	Prospetively	Seychelles	Africa	Eastern Africa	High-income economies	2004	Adults: 18+ years	Apparently healthy individuals	NCEP-ATP III, 2001
83	Bune, 2019_Ethiopia	The global magnitude of metabolic syndrome among antiretroviral therapy (ART) exposed and ART-naive adult HIV-infected patients in gedio-zone, southern Ethiopia: Comparative cross-sectional study, using the Adult Treatment Panel III criteria.	Cross-sectional	Probabilistic	Simple random sampling	Hospital-based	Multicenter	Prospetively	Ethiopia	Africa	Eastern Africa	Low-income economies	Dec/2017-Jan/2019	Adults: 18+ years	HIV infected patients	Revised NCEP-ATP III, 2005
85	Chanda, 2010_Zambia	Predictive value of Metabolic Syndrome components in detecting the syndrome in patients with type 2 Diabetes Mellitus.	Cross-sectional	Non probabilistic	Consecutive sampling	Hospital-based	Monocenter	Prospetively	Zambia	Africa	Eastern Africa	Lower-middle income economies	Unclear/ Not reported	Adults: 18+ years	Type 2 diabetes patients	NCEP-ATP III, 2001
86	Chivese, 2019_South Africa	High prevalence of cardiovascular risk factors and insulin resistance 6 years after hyperglycemia first detected in pregnancy in Cape Town, South Africa.	Cross-sectional	Non probabilistic	Consecutive sampling	Hospital-based	Monocenter	Prospetively	South Africa	Africa	Southern Africa	Upper-middle-income economies	Aug/2010-Mar/2017	Adults: 18+ years	Type 2 diabetes patients	NCEP-ATP III, 2001
88	Cissé, 2021_Burkina Faso	Using the first nationwide survey on non-communicable disease risk factors and different definitions to evaluate the prevalence of metabolic syndrome in Burkina Faso.	Cross-sectional	Probabilistic	Stratified sampling	Community-based	Nationally representative	Prospetively	Burkina Faso	Africa	West Africa	Low-income economies	Sep/2013-Nov/2013	Adults: 18+ years	Apparently healthy individuals	JIS, 2009
89	Cole, 2017_Ghana	Sleep duration is associated with increased risk for cardiovascular outcomes: a pilot study in a sample of community dwelling adults in Ghana.	Cross-sectional	Probabilistic	Simple random sampling	Community-based	Multicenter	Prospetively	Ghana	Africa	West Africa	Lower-middle income economies	Unclear/ Not reported	Adults: 18+ years	Apparently healthy individuals	JIS, 2009
90	Dada, 2016_Nigeria	Metabolic Syndrome and Framingham Risk Score: Observation from Screening of Low-Income Semi-Urban African Women.	Cross-sectional	Non probabilistic	Consecutive sampling	Community-based	Monocenter	Prospetively	Nigeria	Africa	West Africa	Lower-middle income economies	Unclear/ Not reported	Adults: 18+ years	Apparently healthy individuals	IDF, 2005
91	Distiller, 2010_South Africa	The effect of features of the metabolic syndrome on atherosclerotic risk in relatively long-surviving patients with type 1 diabetes.	Cross-sectional	Non probabilistic	Consecutive sampling	Hospital-based	Monocenter	Prospetively	South Africa	Africa	Southern Africa	Upper-middle-income economies	Unclear/ Not reported	Adults: 18+ years	Patients with type 1 diabetes	IDF, 2005
92	Dos Prazeres Tavares, 2016_Angola	Prevalence of metabolic syndrome in non-diabetic, pregnant Angolan women according to four diagnostic criteria and its effects on adverse perinatal outcomes.	Cross-sectional	Non probabilistic	Consecutive sampling	Hospital-based	Monocenter	Prospetively	Angola	Africa	Central Africa	Lower-middle income economies	Dec/2014-Feb/2015	Unclear/Not reported	Pregnant women	JIS, 2009
93	Doualla-Bija, 2018_Cameroon	Prevalence and characteristics of metabolic syndrome in gout patients in a hospital setting in sub-Saharan Africa.	Cross-sectional	Non probabilistic	Consecutive sampling	Hospital-based	Monocenter	Prospetively	Cameroon	Africa	Central Africa	Lower-middle income economies	Dec/2016-May/2017	Adults: 18+ years	Patients with Chronic musculoskeletal diseases	IDF, 2005
94	Duki, 2016_South Africa	Relationship of Body Anthropometry with Cardiovascular Risk Factors in a Random Community Sample: The Phoenix Lifestyle Project.	Cross-sectional	Probabilistic	Simple random sampling	Community-based	Monocenter	Prospetively	South Africa	Africa	Southern Africa	Upper-middle-income economies	Unclear/ Not reported	Adults: 18+ years	Apparently healthy individuals	WHO,1998
95	El Ati, 2020_Tunisia	Metabolic syndrome, malnutrition, and its associations with cardiovascular and all-cause mortality in hemodialysis patients: Follow-up for three years.	Cross-sectional	Non probabilistic	Consecutive sampling	Hospital-based	Monocenter	Prospetively	Tunisia	Eastern Mediterranean	Northern Africa	Lower-middle income economies	Jan/2013-Dec/2016	Adults: 18+ years	Patients with chronic kidney diseases	NCEP-ATP III, 2001
96	El Brini, 2014_Morocco	Prevalence of metabolic syndrome and its components based on a harmonious definition among adults in Morocco.	Cross-sectional	Non probabilistic	Consecutive sampling	Hospital-based	Monocenter	Retrospectively	Morocco	Eastern Mediterranean	Northern Africa	Lower-middle income economies	Oct/2010-May/2012	Adults: 18+ years	Apparently healthy individuals	JIS, 2009
97	El Maghraoui, 2014_Morocco	Osteoporosis, vertebral fractures and metabolic syndrome in postmenopausal women.	Cross-sectional	Non probabilistic	Consecutive sampling	Hospital-based	Monocenter	Prospetively	Morocco	Eastern Mediterranean	Northern Africa	Lower-middle income economies	jun/2012-mar/2013	Adults: 18+ years	Postmenopausal women	NCEP-ATP III, 2001
98	El Ray, 2013_Egypt	Insulin resistance: a major factor associated with significant liver fibrosis in Egyptian patients with genotype 4 chronic hepatitis C.	Cross-sectional	Non probabilistic	Consecutive sampling	Hospital-based	Monocenter	Prospetively	Egypt	Eastern Mediterranean	Northern Africa	Lower-middle income economies	Unclear/ Not reported	Adults: 18+ years	Patients with chronic diseases	Revised NCEP-ATP III, 2005
99	El Sayed, 2016_Egypt	Association of metabolic syndrome with female pattern hair loss in women: A case-control study.	Cross-sectional	Non probabilistic	Consecutive sampling	Hospital-based	Monocenter	Prospetively	Egypt	Eastern Mediterranean	Northern Africa	Lower-middle income economies	Unclear/ Not reported	Adults: 18+ years	Patients with dermatological diseases	NCEP-ATP III, 2001
100	El-Karaksy, 2015_Egypt	The value of different insulin resistance indices in assessment of non-alcoholic fatty liver disease in overweight/obese children.	Cross-sectional	Non probabilistic	Consecutive sampling	Hospital-based	Monocenter	Prospetively	Egypt	Eastern Mediterranean	Northern Africa	Lower-middle income economies	Jun/2008-May/2009	Children : Birth-18 years	Patients with obesity	NCEP-ATP III, 2001
101	Elkehili, 2012_Libya	Prevalence of the metabolic syndrome in renal transplant recipients.	Cross-sectional	Non probabilistic	Consecutive sampling	Hospital-based	Monocenter	Prospetively	Libya	Eastern Mediterranean	Northern Africa	Upper-middle-income economies	Unclear/ Not reported	Unclear/Not reported	Patients with chronic kidney diseases	NCEP-ATP III, 2001
102	El-Koofy, 2012_Egypt	The association of metabolic syndrome, insulin resistance and non-alcoholic fatty liver disease in overweight/obese children.	Cross-sectional	Non probabilistic	Consecutive sampling	Hospital-based	Monocenter	Prospetively	Egypt	Eastern Mediterranean	Northern Africa	Lower-middle income economies	Unclear/ Not reported	Children : Birth-18 years	Patients with obesity	NCEP-ATP III, 2001
103	Elsehely, 2017_Egypt	A Cutoff for Age at Menarche Predicting Metabolic Syndrome in Egyptian Overweight/Obese Premenopausal Women.	Cross-sectional	Non probabilistic	Consecutive sampling	Hospital-based	Monocenter	Prospetively	Egypt	Eastern Mediterranean	Northern Africa	Lower-middle income economies	Unclear/ Not reported	Adults: 18+ years	Patients with obesity	NCEP-ATP III, 2001
104	Emma-Okon, 2014_Nigeria	The Metabolic Syndrome in an Elite African Community.	Cross-sectional	Non probabilistic	Consecutive sampling	Community-based	Monocenter	Prospetively	Nigeria	Africa	West Africa	Lower-middle income economies	Unclear/ Not reported	Adults: 18+ years	Apparently healthy individuals	IDF, 2005
105	Ephraim, 2020_Ghana	Predicting type 2 diabetes mellitus among fishermen in Cape Coast: a comparison between the FINDRISC score and the metabolic syndrome.	Cross-sectional	Non probabilistic	Consecutive sampling	Community-based	Multicenter	Prospetively	Ghana	Africa	West Africa	Lower-middle income economies	2019	Unclear/Not reported	Apparently healthy individuals	IDF, 2005
106	Erasmus, 2012_South Africa	High prevalence of diabetes mellitus and metabolic syndrome in a South African coloured population: baseline data of a study in Bellville, Cape Town.	Cross-sectional	Probabilistic	Simple random sampling	Community-based	Multicenter	Prospetively	South Africa	Africa	Southern Africa	Upper-middle-income economies	Jan/2008-Mar/2009	Adults: 18+ years	Apparently healthy individuals	JIS, 2009
107	Ezeala, 2019_Nigeria	Prevalence and Risk Factors for Metabolic Syndrome among Hypertensive Patients Attending the General Outpatient Clinic of a Tertiary Hospital in North Central Nigeria.	Cross-sectional	Non probabilistic	Consecutive sampling	Hospital-based	Monocenter	Retrospectively	Nigeria	Africa	West Africa	Lower-middle income economies	Sep/2014-Oct/2014	Unclear/Not reported	Hypertensive patients	NCEP-ATP III, 2001
108	Ezzaher, 2011_Tunisia	Metabolic syndrome in Tunisian bipolar I patients.	Cross-sectional	Non probabilistic	Consecutive sampling	Hospital-based	Monocenter	Prospetively	Tunisia	Eastern Mediterranean	Northern Africa	Lower-middle income economies	Unclear/ Not reported	Adults: 18+ years	Psychiatric patients	NCEP-ATP III, 2001
109	Faasen, 2014_South Africa	Undiagnosed metabolic syndrome and other adverse effects among clozapine users of Xhosa descent.	Cross-sectional	Non probabilistic	Convenience sampling	Community-based	Monocenter	Prospetively	South Africa	Africa	Southern Africa	Upper-middle-income economies	Unclear/ Not reported	Unclear/Not reported	Psychiatric patients	IDF, 2005
110	Fahmi, 2020_Egypt	Metabolic syndrome components and disease disability in egyptian multiple sclerosis patients.	Cross-sectional	Non probabilistic	Consecutive sampling	Hospital-based	Monocenter	Prospetively	Egypt	Eastern Mediterranean	Northern Africa	Lower-middle income economies	Dec/2018-Jun/2019	Adults: 18+ years	Patients with Chronic musculoskeletal diseases	NCEP-ATP III, 2001
111	Fanta, 2021_Ethiopia	Prevalence and Impact of Metabolic Syndrome on Short-Term Prognosis in Patients with Acute Coronary Syndrome: Prospective Cohort Study.	Cross-sectional	Non probabilistic	Consecutive sampling	Hospital-based	Multicenter	Prospetively	Ethiopia	Africa	Eastern Africa	Low-income economies	Mar/2018-Nov/2018	Adults: 18+ years	Patients with cardiovascular diseases	JIS, 2009
115	Fezeu, 2007_Cameroon	Metabolic syndrome in a sub-Saharan African setting: central obesity may be the key determinant.	Cross-sectional	Non probabilistic	Consecutive sampling	Community-based	Multicenter	Prospetively	Cameroon	Africa	Central Africa	Lower-middle income economies	Unclear/ Not reported	Adults: 18+ years	Apparently healthy individuals	WHO,1998
116	Fouad, 2016_Egypt	Prevalence of obesity and risk of chronic kidney disease among young adults in Egypt.	Cross-sectional	Non probabilistic	Consecutive sampling	Community-based	Monocenter	Prospetively	Egypt	Eastern Mediterranean	Northern Africa	Lower-middle income economies	Jan/2014-Apr/2015	Adults: 18+ years	Apparently healthy individuals	Revised NCEP-ATP III, 2005
117	Fourie, 2010_South Africa	Lipid abnormalities in a never-treated HIV-1 subtype C-infected African population.	Case control	Probabilistic	Simple random sampling	Hospital/community based	Multicenter	Prospetively	South Africa	Africa	Southern Africa	Upper-middle-income economies	Unclear/ Not reported	Adults: 18+ years	Apparently healthy individuals	IDF, 2005
118	Fourie, 2010_South Africa	Lipid abnormalities in a never-treated HIV-1 subtype C-infected African population.	Case control	Probabilistic	Simple random sampling	Hospital/community based	Multicenter	Prospetively	South Africa	Africa	Southern Africa	Upper-middle-income economies	Unclear/ Not reported	Adults: 18+ years	HIV infected patients	IDF, 2005
119	Frigati, 2019_South Africa	Insulin Resistance in South African Youth Living with Perinatally Acquired HIV Receiving Antiretroviral Therapy.	Cross-sectional	Non probabilistic	Consecutive sampling	Hospital-based	Multicenter	Prospetively	South Africa	Africa	Southern Africa	Upper-middle-income economies	Jul/2013-Mar/2015	Children : Birth-18 years	Apparently healthy individuals	IDF, 2005
120	Frigati, 2019_South Africa	Insulin Resistance in South African Youth Living with Perinatally Acquired HIV Receiving Antiretroviral Therapy.	Cross-sectional	Non probabilistic	Consecutive sampling	Hospital-based	Multicenter	Prospetively	South Africa	Africa	Southern Africa	Upper-middle-income economies	Jul/2013-Mar/2015	Children : Birth-18 years	HIV infected patients	IDF, 2005
121	Gannar, 2015_Tunisia	Social class and metabolic syndrome in populations from Tunisia and Spain.	Cross-sectional	Probabilistic	Simple random sampling	Hospital/community based	Multicenter	Prospetively	Tunisia	Eastern Mediterranean	Northern Africa	Lower-middle income economies	Dec/2013-May/2014	Adults: 18+ years	Apparently healthy individuals	JIS, 2009
122	Garrido, 2009_Botswana	Metabolic syndrome and obesity among workers at Kanye Seventh-Day Adventist Hospital, Botswana.	Cross-sectional	Non probabilistic	Consecutive sampling	Hospital-based	Monocenter	Prospetively	Botswana	Africa	Southern Africa	Upper-middle-income economies	Dec/2007-Feb/2008	Adults: 18+ years	Apparently healthy individuals	NCEP-ATP III, 2001
123	Gebreegziabiher, 2021_Ethiopia	Magnitude and Associated Factors of Metabolic Syndrome Among Adult Urban Dwellers of Northern Ethiopia.	Cross-sectional	Probabilistic	Simple random sampling	Community-based	Monocenter	Prospetively	Ethiopia	Africa	Eastern Africa	Low-income economies	Jul/2019-Sep/2019	Adults: 18+ years	Apparently healthy individuals	IDF, 2005
124	Gebremariam, 2018_Ethiopia	Non-communicable disease risk factor profile among public employees in a regional city in northern Ethiopia.	Cross-sectional	Non probabilistic	Consecutive sampling	Hospital/community based	Multicenter	Prospetively	Ethiopia	Africa	Eastern Africa	Low-income economies	Oct/2015-Feb/2016	Adults: 18+ years	Apparently healthy individuals	JIS, 2009
125	Gebremeskel, 2019_Ethiopia	Magnitude of metabolic syndrome and its associated factors among patients with type 2 diabetes mellitus in Ayder Comprehensive Specialized Hospital, Tigray, Ethiopia: a cross sectional study.	Cross-sectional	Non probabilistic	Consecutive sampling	Hospital-based	Monocenter	Prospetively	Ethiopia	Africa	Eastern Africa	Low-income economies	Feb/2018-Jun/2018	Adults: 18+ years	Type 2 diabetes patients	IDF, 2005
126	Gebreyes, 2018_Ethiopia	Prevalence of high bloodpressure, hyperglycemia, dyslipidemia, metabolic syndrome and their determinants in Ethiopia: Evidences from the National NCDs STEPS Survey, 2015.	Cross-sectional	Probabilistic	Stratified sampling	Hospital/community based	Nationally representative	Prospetively	Ethiopia	Africa	Eastern Africa	Low-income economies	Apr/2015-Jun/2015	All ages	Apparently healthy individuals	IDF, 2005
128	Gebrie, 2020_Ethiopia	The burden of metabolic syndrome in patients living with HIV/AIDS receiving care at referral hospitals of Northwest Ethiopia: A hospital-based cross-sectional study, 2019.	Cross-sectional	Probabilistic	Simple random sampling	Hospital-based	Multicenter	Prospetively	Ethiopia	Africa	Eastern Africa	Low-income economies	Feb/2019-Apr/2019	Adults: 18+ years	HIV infected patients	IDF, 2005
129	George, 2013_South Africa	The association of 25 hydroxyvitamin D and parathyroid hormone with metabolic syndrome in two ethnic groups in South Africa.	Cross-sectional	Non probabilistic	Consecutive sampling	Community-based	Monocenter	Prospetively	South Africa	Africa	Southern Africa	Upper-middle-income economies	Unclear/ Not reported	Adults: 18+ years	Apparently healthy individuals	JIS, 2009
131	Ghazali, 2010_Nigeria	Waist circumference, waist to hip ratio, and body mass index in the diagnosis of metabolic syndrome in Nigerian subjects.	Cross-sectional	Non probabilistic	Consecutive sampling	Hospital/community based	Multicenter	Prospetively	Nigeria	Africa	West Africa	Lower-middle income economies	Unclear/ Not reported	Adults: 18+ years	Apparently healthy individuals	NCEP-ATP III, 2001
132	Gombet, 2012_Republic of the Congo	Aging, female sex, migration, elevated HDL-C, and inflammation are associated with prevalence of metabolic syndrome among African bank employees.	Cross-sectional	Non probabilistic	Consecutive sampling	Community-based	Multicenter	Prospetively	Republic of the Congo	Africa	Central Africa	Lower-middle income economies	2008	Unclear/Not reported	Apparently healthy individuals	IDF, 2005
133	Goolam Mahyoodeen, 2019_South Africa	High burden of the metabolic syndrome and its component disorders in South Africans with psoriasis.	Cross-sectional	Non probabilistic	Consecutive sampling	Hospital-based	Monocenter	Prospetively	South Africa	Africa	Southern Africa	Upper-middle-income economies	Unclear/ Not reported	Adults: 18+ years	Apparently healthy individuals	JIS, 2009
134	Goolam Mahyoodeen, 2019_South Africa	High burden of the metabolic syndrome and its component disorders in South Africans with psoriasis.	Cross-sectional	Non probabilistic	Consecutive sampling	Hospital-based	Monocenter	Prospetively	South Africa	Africa	Southern Africa	Upper-middle-income economies	Unclear/ Not reported	Adults: 18+ years	Patients with dermatological diseases	JIS, 2009
135	Govender, 2020_South Africa	A multicentre cross-sectional descriptive study evaluating the cardiovascular risk profile of preoperatively identified patients with hypertension.	Cross-sectional	Non probabilistic	Consecutive sampling	Hospital-based	Multicenter	Prospetively	South Africa	Africa	Southern Africa	Upper-middle-income economies	Unclear/ Not reported	Adults: 18+ years	Hypertensive patients	Revised NCEP-ATP III, 2005
136	Guira, 2016_Burkina Faso	Features of Metabolic Syndrome and Its Associated Factors during Highly Active Antiretroviral Therapy in Ouagadougou (Burkina Faso).	Cross-sectional	Non probabilistic	Consecutive sampling	Hospital-based	Monocenter	Prospetively	Burkina Faso	Africa	West Africa	Low-income economies	2011	Adults: 18+ years	HIV infected patients	IDF, 2005
137	Gyakobo, 2012_Ghana	Prevalence of the metabolic syndrome in a rural population in Ghana.	Cross-sectional	Non probabilistic	Consecutive sampling	Community-based	Multicenter	Prospetively	Ghana	Africa	West Africa	Lower-middle income economies	2007	Adults: 18+ years	Apparently healthy individuals	IDF, 2005
140	Hamlaoui, 2018_Algeria	Relationship of metabolic syndrome and its components with thyroid dysfunction in Algerian patients.	Cross-sectional	Non probabilistic	Consecutive sampling	Hospital-based	Monocenter	Prospetively	Algeria	Africa	Northern Africa	Lower-middle income economies	Jan/2014-Sep/2015	Adults: 18+ years	Patients with thyroid disease	Revised NCEP-ATP III, 2005
141	Hammam, 2018_Egypt	Metabolic syndrome in systemic lupus erythematosus patients : Relationship to disease activity and neuropsychiatric lupus.	Cross-sectional	Non probabilistic	Consecutive sampling	Hospital-based	Monocenter	Prospetively	Egypt	Eastern Mediterranean	Northern Africa	Lower-middle income economies	2015-2016	Adults: 18+ years	Patients with dermatological diseases	NCEP-ATP III, 2001
142	Hamooya , 2021_Zambia	Metabolic syndrome in Zambian adults with human immunodeficiency virus on antiretroviral therapy: Prevalence and associated factors.	Cross-sectional	Probabilistic	Systematic sampling	Hospital-based	Multicenter	Prospetively	Zambia	Africa	Eastern Africa	Lower-middle income economies	Apr/2019-May/2020	Adults: 18+ years	HIV infected patients	JIS, 2009
144	Hannoun, 2021_Morocco	[Study of the metabolic syndrome and physical activity in a population from Marrakesh, in Morocco].	Cross-sectional	Non probabilistic	Consecutive sampling	Hospital-based	Monocenter	Prospetively	Morocco	Eastern Mediterranean	Northern Africa	Lower-middle income economies	Mars/2018-Juil/2018	Adults: 18+ years	Apparently healthy individuals	NCEP-ATP III, 2001
145	Harrabi , 2009_Tunisia	Prevalence of the metabolic syndrome among urban schoolchildren in Sousse, Tunisia.	Cross-sectional	Non probabilistic	Consecutive sampling	Community-based	Monocenter	Prospetively	Tunisia	Eastern Mediterranean	Northern Africa	Lower-middle income economies	Unclear/ Not reported	Children : Birth-18 years	Apparently healthy individuals	NCEP-ATP III, 2001
147	Harzallah, 2006_Tunisia	The metabolic syndrome in an Arab population: a first look at the new International Diabetes Federation criteria.	Cross-sectional	Probabilistic	Simple random sampling	Hospital-based	Monocenter	Retrospectively	Tunisia	Eastern Mediterranean	Northern Africa	Lower-middle income economies	1985-1994	Adults: 18+ years	Apparently healthy individuals	IDF, 2005
148	Hasanain, 2017_Egypt	Erectile dysfunction in patients with nonalcoholic fatty liver disease.	Cross-sectional	Non probabilistic	Consecutive sampling	Hospital-based	Monocenter	Prospetively	Egypt	Eastern Mediterranean	Northern Africa	Lower-middle income economies	Apr/2013-Feb/2015	All ages	Patients with nonalcoholic fatty liver disease	NCEP-ATP III, 2001
149	Hasni , 2019_Tunisia	Biochemical Data and Metabolic Profiles of Male Exclusive Narghile Smokers (ENSs) Compared With Apparently Healthy Nonsmokers (AHNSs).	Cross-sectional	Non probabilistic	Consecutive sampling	Hospital-based	Monocenter	Prospetively	Tunisia	Eastern Mediterranean	Northern Africa	Lower-middle income economies	Dec/2015-Feb/2016	Adults: 18+ years	Apparently healthy individuals	IDF, 2005
150	Hasni , 2019_Tunisia	Biochemical Data and Metabolic Profiles of Male Exclusive Narghile Smokers (ENSs) Compared With Apparently Healthy Nonsmokers (AHNSs).	Cross-sectional	Non probabilistic	Consecutive sampling	Hospital-based	Monocenter	Prospetively	Tunisia	Eastern Mediterranean	Northern Africa	Lower-middle income economies	Dec/2015-Feb/2016	Adults: 18+ years	Exclusive narghile smokers	IDF, 2005
151	Hassan, 2015_Egypt	Is Neck Circumference an Indicator for Metabolic Complication of Childhood Obesity?	Cross-sectional	Non probabilistic	Consecutive sampling	Hospital-based	Monocenter	Prospetively	Egypt	Eastern Mediterranean	Northern Africa	Lower-middle income economies	Apr/2013-Jan/2014	Children : Birth-18 years	Apparently healthy individuals	NCEP-ATP III, 2001
152	Hassan, 2015_Egypt	Is Neck Circumference an Indicator for Metabolic Complication of Childhood Obesity?	Cross-sectional	Non probabilistic	Consecutive sampling	Hospital-based	Monocenter	Prospetively	Egypt	Eastern Mediterranean	Northern Africa	Lower-middle income economies	Apr/2013-Jan/2014	Children : Birth-18 years	Patients with obesity	NCEP-ATP III, 2001
153	Hassan, 2019_Egypt	Metabolic syndrome in a sample of Egyptian adolescent girls and its association with apolipoprotein E.	Cross-sectional	Non probabilistic	Consecutive sampling	Hospital-based	Monocenter	Prospetively	Egypt	Eastern Mediterranean	Northern Africa	Lower-middle income economies	Feb/2013-Mar/2016	Children : Birth-18 years	Apparently healthy individuals	IDF, 2005
154	Hassan, 2019_Egypt	Metabolic syndrome in a sample of Egyptian adolescent girls and its association with apolipoprotein E.	Cross-sectional	Non probabilistic	Consecutive sampling	Hospital-based	Monocenter	Prospetively	Egypt	Eastern Mediterranean	Northern Africa	Lower-middle income economies	Feb/2013-Mar/2016	Children : Birth-18 years	Patients with obesity	IDF, 2005
155	Hirigo , 2016_Ethiopia	Influences of gender in metabolic syndrome and its components among people living with HIV virus using antiretroviral treatment in Hawassa, southern Ethiopia.	Cross-sectional	Non probabilistic	Consecutive sampling	Hospital-based	Monocenter	Prospetively	Ethiopia	Africa	Eastern Africa	Low-income economies	2013	All ages	HIV infected patients	IDF, 2005
157	Hoebel, 2014_South Africa	Optimizing waist circumference cut-points for the metabolic syndrome in a South African cohort at 3-year follow-up: the SABPA prospective cohort.	Cohort (Baseline data)	Non probabilistic	Consecutive sampling	Community-based	Monocenter	Prospetively	South Africa	Africa	Southern Africa	Upper-middle-income economies	Feb/2011-May/2011	Adults: 18+ years	Apparently healthy individuals	JIS, 2009
156	Hoebel , 2012_South Africa	Determining cut-off values for neck circumference as a measure of the metabolic syndrome amongst a South African cohort: the SABPA study.	Cross-sectional	Non probabilistic	Consecutive sampling	Hospital-based	Monocenter	Prospetively	South Africa	Africa	Southern Africa	Upper-middle-income economies	Feb/2008-May/2009	Adults: 18+ years	Apparently healthy individuals	JIS, 2009
158	Houti , 2016_Algeria	Prevalence of Metabolic Syndrome and its Related Risk Factors in the City of Oran, Algeria: the ISOR Study.	Cross-sectional	Probabilistic	Simple random sampling	Hospital-based	Monocenter	Prospetively	Algeria	Africa	Northern Africa	Lower-middle income economies	2007-2009	Adults: 18+ years	Apparently healthy individuals	NCEP-ATP III, 2001
159	Isezuo, 2005_Nigeria	Is high density lipoprotein cholesterol useful in diagnosis of metabolic syndrome in native Africans with type 2 diabetes?	Cross-sectional	Non probabilistic	Consecutive sampling	Hospital-based	Monocenter	Prospetively	Nigeria	Africa	West Africa	Lower-middle income economies	Unclear/ Not reported	Adults: 18+ years	Type 2 diabetes patients	WHO,1998
160	Ishaku, 2021_Nigeria	Metabolic syndrome following hypertensive disorders in pregnancy in a low-resource setting: A cohort study.	Cohort (Baseline data)	Non probabilistic	Consecutive sampling	Hospital-based	Multicenter	Prospetively	Nigeria	Africa	West Africa	Lower-middle income economies	Aug/2017-Apr/2018	Adults: 18+ years	Apparently healthy individuals	IDF, 2005
161	Ishaku, 2021_Nigeria	Metabolic syndrome following hypertensive disorders in pregnancy in a low-resource setting: A cohort study.	Cohort (Baseline data)	Non probabilistic	Consecutive sampling	Hospital-based	Multicenter	Prospetively	Nigeria	Africa	West Africa	Lower-middle income economies	Aug/2017-Apr/2018	Adults: 18+ years	Hypertensive patients	IDF, 2005
164	Jamoussi , 2012_Tunisia	Metabolic syndrome in Tunisian obese children and adolescents.	Cross-sectional	Non probabilistic	Consecutive sampling	Hospital-based	Monocenter	Prospetively	Tunisia	Eastern Mediterranean	Northern Africa	Lower-middle income economies	Dec/2007-Oct/2008	Children : Birth-18 years	Patients with obesity	IDF, 2005
165	Jean-Luc Gradidge, 2016_South Africa	Metabolic and Body Composition Risk Factors Associated with Metabolic Syndrome in a Cohort of Women with a High Prevalence of Cardiometabolic Disease.	Cross-sectional	Non probabilistic	Consecutive sampling	Hospital-based	Monocenter	Prospetively	South Africa	Africa	Southern Africa	Upper-middle-income economies	Unclear/ Not reported	Adults: 18+ years	Apparently healthy individuals	JIS, 2009

166	Jennings, 2009_South Africa	The atypical presentation of the metabolic syndrome components in black African women: the relationship with insulin resistance and the influence of regional adipose tissue distribution.	Cross-sectional	Non probabilistic	Consecutive sampling	Community-based	Multicenter	Prospectively	South Africa	Africa	Southern Africa	Upper-middle-income economies	Unclear/ Not reported	Adults: 18+ years	Apparently healthy individuals	IDF, 2005
167	Jennings, 2009_South Africa	The atypical presentation of the metabolic syndrome components in black African women: the relationship with insulin resistance and the influence of regional adipose tissue distribution.	Cross-sectional	Non probabilistic	Consecutive sampling	Community-based	Multicenter	Prospectively	South Africa	Africa	Southern Africa	Upper-middle-income economies	Unclear/ Not reported	Adults: 18+ years	Patients with obesity	IDF, 2005
168	Jmal, 2019_Tunisia	Prevalence of metabolic syndrome in Tunisian overweight and obese children.	Cross-sectional	Non probabilistic	Consecutive sampling	Hospital-based	Monocenter	Retrospectively	Tunisia	Eastern Mediterranean	Northern Africa	Lower-middle income economies	Unclear/ Not reported	Children : Birth-18 years	Apparently healthy individuals	IDF, 2005
169	Jmal, 2019_Tunisia	Prevalence of metabolic syndrome in Tunisian overweight and obese children.	Cross-sectional	Non probabilistic	Consecutive sampling	Hospital-based	Monocenter	Retrospectively	Tunisia	Eastern Mediterranean	Northern Africa	Lower-middle income economies	Unclear/ Not reported	Children : Birth-18 years	Patients with obesity	IDF, 2005
170	Kacem, 2019_Tunisia	Evaluation of the risk of metabolic syndrome among shift workers in Tunisia.	Cross-sectional	Non probabilistic	Consecutive sampling	Community-based	Monocenter	Prospectively	Tunisia	Eastern Mediterranean	Northern Africa	Lower-middle income economies	Unclear/ Not reported	Unclear/Not reported	Apparently healthy individuals	IDF, 2005
171	Kaduka, 2012_Kenya	Prevalence of metabolic syndrome among an urban population in Kenya.	Cross-sectional	Probabilistic	Cluster sampling	Community-based	Multicenter	Prospectively	Kenya	Africa	Eastern Africa	Lower-middle income economies	2008	Adults: 18+ years	Apparently healthy individuals	JIS, 2009
172	Kalk , 2008_South Africa	The metabolic syndrome, insulin resistance, and its surrogates in African and white subjects with type 2 diabetes in South Africa.	Cross-sectional	Non probabilistic	Consecutive sampling	Hospital-based	Monocenter	Prospectively	South Africa	Africa	Southern Africa	Upper-middle-income economies	1994-2002	All ages	Type 2 diabetes patients	IDF, 2005
173	Katchunga, 2010_Democratic Republic of the Congo	[Hypertension, insulin resistance and chronic kidney disease in type 2 diabetes patients from South Kivu, DR Congo].	Cross-sectional	Non probabilistic	Consecutive sampling	Hospital-based	Monocenter	Prospectively	Democratic Republic of the Congo	Africa	Central Africa	Low-income economies	2005-2007	Adults: 18+ years	Type 2 diabetes patients	JIS, 2009
174	Katoto, 2018_Democratic Republic of the Congo	Prevalence and risk factors of metabolic syndrome in HIV-infected adults at three urban clinics in a post-conflict setting, eastern Democratic Republic of the Congo.	Cross-sectional	Non probabilistic	Consecutive sampling	Hospital-based	Multicenter	Prospectively	Democratic Republic of the Congo	Africa	Central Africa	Low-income economies	2016	Adults: 18+ years	HIV infected patients	IDF, 2005
175	Kelliny, 2008_Seychelles	Metabolic syndrome according to different definitions in a rapidly developing country of the African region.	Cross-sectional	Non probabilistic	Consecutive sampling	Community-based	Multicenter	Retrospectively	Seychelles	Africa	Eastern Africa	High-income economies	2004	Adults: 18+ years	Apparently healthy individuals	IDF, 2005
176	Kengne, 2012_Cameroon	Metabolic syndrome in type 2 diabetes: comparative prevalence according to two sets of diagnostic criteria in sub-Saharan Africans.	Cross-sectional	Non probabilistic	Consecutive sampling	Hospital-based	Monocenter	Prospectively	Cameroon	Africa	Central Africa	Lower-middle income economies	2006-2008	Adults: 18+ years	Type 2 diabetes patients	IDF, 2005
177	Ker, 2007_South Africa	Frequency of the metabolic syndrome in screened South African corporate executives.	Cross-sectional	Non probabilistic	Consecutive sampling	Community-based	Multicenter	Prospectively	South Africa	Africa	Southern Africa	Upper-middle-income economies	Unclear/ Not reported	Adults: 18+ years	Apparently healthy individuals	NCEP-ATP III, 2001
178	Kerie, 2019_Ethiopia	Metabolic syndrome among residents of Mizan-Aman town, South West Ethiopia, 2017: A cross sectional study.	Cross-sectional	Non probabilistic	Consecutive sampling	Community-based	Multicenter	Prospectively	Ethiopia	Africa	Eastern Africa	Low-income economies	2017	Adults: 18+ years	Apparently healthy individuals	NCEP-ATP III, 2001
179	Khalfa, 2017_Algeria	Prevalence of metabolic syndrome and its association with lifestyle and cardiovascular biomarkers among postmenopausal women in western Algeria.	Cross-sectional	Non probabilistic	Consecutive sampling	Hospital-based	Monocenter	Prospectively	Algeria	Africa	Northern Africa	Lower-middle income economies	2015	Unclear/Not reported	Postmenopausal women	NCEP-ATP III, 2001
180	Kiama, 2018_Kenya	Prevalence and factors associated with metabolic syndrome in an urban population of adults living with HIV in Nairobi, Kenya.	Cross-sectional	Probabilistic	Systematic sampling	Hospital-based	Monocenter	Prospectively	Kenya	Africa	Eastern Africa	Lower-middle income economies	May/2016-Jun/2016	Adults: 18+ years	HIV infected patients	JIS, 2009
181	Kingery, 2016_Tanzania	Short-term and long-term cardiovascular risk, metabolic syndrome and HIV in Tanzania.	Cross-sectional	Non probabilistic	Consecutive sampling	Hospital-based	Monocenter	Prospectively	Tanzania	Africa	Eastern Africa	Lower-middle income economies	Oct/2012-Apr/2013	Adults: 18+ years	Apparently healthy individuals	IDF, 2005
182	Kingery, 2016_Tanzania	Short-term and long-term cardiovascular risk, metabolic syndrome and HIV in Tanzania.	Cross-sectional	Non probabilistic	Consecutive sampling	Hospital-based	Monocenter	Prospectively	Tanzania	Africa	Eastern Africa	Lower-middle income economies	Oct/2012-Apr/2013	Adults: 18+ years	HIV infected patients	IDF, 2005
183	Kingue, 2017_Cameroon	Prevalence of selected cardiometabolic risk factors among adults in urban and semi-urban hospitals in four sub-Saharan African countries.	Cross-sectional	Non probabilistic	Consecutive sampling	Hospital-based	Multicenter	Prospectively	Cameroon	Africa	Central Africa	Lower-middle income economies	Dec/2011-Feb/2013	Adults: 18+ years	Apparently healthy individuals	IDF, 2005
184	Kingue, 2017_Democratic Republic of the Congo	Prevalence of selected cardiometabolic risk factors among adults in urban and semi-urban hospitals in four sub-Saharan African countries.	Cross-sectional	Non probabilistic	Consecutive sampling	Hospital-based	Multicenter	Prospectively	Democratic Republic of the Congo	Africa	Central Africa	Low-income economies	Dec/2011-Feb/2013	Adults: 18+ years	Apparently healthy individuals	IDF, 2005
185	Kingue, 2017_Madagascar	Prevalence of selected cardiometabolic risk factors among adults in urban and semi-urban hospitals in four sub-Saharan African countries.	Cross-sectional	Non probabilistic	Consecutive sampling	Hospital-based	Multicenter	Prospectively	Madagascar	Africa	Eastern Africa	Low-income economies	Dec/2011-Feb/2013	Adults: 18+ years	Apparently healthy individuals	IDF, 2005
186	Kingue, 2017_Nigeria	Prevalence of selected cardiometabolic risk factors among adults in urban and semi-urban hospitals in four sub-Saharan African countries.	Cross-sectional	Non probabilistic	Consecutive sampling	Hospital-based	Multicenter	Prospectively	Nigeria	Africa	West Africa	Lower-middle income economies	Dec/2011-Feb/2013	Adults: 18+ years	Apparently healthy individuals	IDF, 2005
187	Kruger, 2017_South Africa	The prevalence of the metabolic syndrome in a farm worker community in the Boland district, South Africa.	Cross-sectional	Non probabilistic	Consecutive sampling	Community-based	Monocenter	Prospectively	South Africa	Africa	Southern Africa	Upper-middle-income economies	Apr/2015-Jun/2015	Adults: 18+ years	Apparently healthy individuals	IDF, 2005
188	Kwobah, 2021_Kenya	Prevalence and correlates of metabolic syndrome and its components in adults with psychotic disorders in Eldoret, Kenya.	Cross-sectional	Non probabilistic	Consecutive sampling	Hospital-based	Multicenter	Prospectively	Kenya	Africa	Eastern Africa	Lower-middle income economies	Jul/2018-Mar/2019	Adults: 18+ years	Apparently healthy individuals	NCEP-ATP III, 2001
189	Kwobah, 2021_Kenya	Prevalence and correlates of metabolic syndrome and its components in adults with psychotic disorders in Eldoret, Kenya.	Cross-sectional	Non probabilistic	Consecutive sampling	Hospital-based	Multicenter	Prospectively	Kenya	Africa	Eastern Africa	Lower-middle income economies	Jul/2018-Mar/2019	Adults: 18+ years	Psychiatric patients	NCEP-ATP III, 2001
190	Labhardt, 2017_Lesotho	Metabolic syndrome in patients on first-line antiretroviral therapy containing zidovudine or tenofovir in rural Lesotho, Southern Africa.	Cross-sectional	Non probabilistic	Consecutive sampling	Hospital-based	Multicenter	Prospectively	Lesotho	Africa	Southern Africa	Lower-middle income economies	May/2014-Jun/2014	Adults: 18+ years	HIV infected patients	IDF, 2005
191	Lajeunesse-Trempe, 2018_Kenya	Anthropometric measures and their association with risk factors for cardio-metabolic diseases in Kenyan adults.	Cross-sectional	Probabilistic	Simple random sampling	Community-based	Multicenter	Prospectively	Kenya	Africa	Eastern Africa	Lower-middle income economies	Aug/2005-Jan/2006	Adults: 18+ years	Apparently healthy individuals	JIS, 2009
192	Lawani, 2009_Nigeria	Prevalence of Metabolic Syndrome in Schizophrenics on Antipsychotics in a Nigerian Psychiatric Hospital.	Cross-sectional	Non probabilistic	Consecutive sampling	Hospital-based	Monocenter	Prospectively	Nigeria	Africa	West Africa	Lower-middle income economies	Apr/2008-Jun/2008	Adults: 18+ years	Psychiatric patients	NCEP-ATP III, 2001
193	Lepira, 2010_Democratic Republic of the Congo	Metabolic syndrome using national cholesterol education program criteria in black patients with arterial hypertension.	Cross-sectional	Non probabilistic	Consecutive sampling	Hospital-based	Monocenter	Prospectively	Democratic Republic of the Congo	Africa	Central Africa	Low-income economies	Unclear/ Not reported	Unclear/Not reported	Hypertensive patients	NCEP-ATP III, 2001
194	Longo-Mbenza, 2011_Democratic Republic of the Congo	The metabolic syndrome in a Congolese population and its implications for metabolic syndrome definitions.	Cross-sectional	Probabilistic	Simple random sampling	Hospital-based	Monocenter	Prospectively	Democratic Republic of the Congo	Africa	Central Africa	Low-income economies	Mar/2003-Dec/2003	Adults: 18+ years	Type 2 diabetes patients	IDF, 2005
195	Maaroganye, 2013_South Africa	The prevalence of metabolic syndrome and its associated factors in long-term patients in a specialist psychiatric hospital in South Africa.	Cross-sectional	Non probabilistic	Convenience sampling	Hospital-based	Monocenter	Prospectively	South Africa	Africa	Southern Africa	Upper-middle-income economies	Nov/2009-Aug/2011	Adults: 18+ years	Psychiatric patients	NCEP-ATP III, 2001
196	Magalhães, 2014_Angola	Prevalence of the metabolic syndrome and determination of optimal cut-off values of waist circumference in university employees from Angola.	Cross-sectional	Non probabilistic	Consecutive sampling	Community-based	Monocenter	Prospectively	Angola	Africa	Central Africa	Lower-middle income economies	2009-2010	Adults: 18+ years	Apparently healthy individuals	JIS, 2009
197	Maistry, 2018_South Africa	Candidate gene polymorphisms related to lipid metabolism in Asian Indians living in Durban, South Africa.	Cross-sectional	Probabilistic	Simple random sampling	Hospital-based	Monocenter	Prospectively	South Africa	Africa	Southern Africa	Upper-middle-income economies	Jan/2007-Dec/2008	All ages	Apparently healthy individuals	JIS, 2009
198	Maoujoud, 2011_Morocco	[Prevalence of metabolic syndrome in chronic haemodialysis patients in Morocco].	Cross-sectional	Non probabilistic	Convenience sampling	Hospital-based	Monocenter	Prospectively	Morocco	Eastern Mediterranean	Northern Africa	Lower-middle income economies	mar/2009	Adults: 18+ years	Patients with chronic kidney diseases	NCEP-ATP III, 2001
199	Marbou, 2019_Cameroon	Prevalence of Metabolic Syndrome and Its Components in Bamboutos Division's Adults, West Region of Cameroon.	Cross-sectional	Probabilistic	Simple random sampling	Hospital-based	Multicenter	Prospectively	Cameroon	Africa	Central Africa	Lower-middle income economies	May/2016-May/2018	Adults: 18+ years	Apparently healthy individuals	JIS, 2009
200	Marceline, 2014_Burkina Faso	[Diagnosis and prevalence of metabolic syndrome in diabetics followed in a context of limited resources: the case of Burkina Faso].	Cross-sectional	Non probabilistic	Consecutive sampling	Hospital-based	Monocenter	Prospectively	Burkina Faso	Africa	West Africa	Low-income economies	Unclear/ Not reported	Unclear/Not reported	Type 2 diabetes patients	JIS, 2009
201	Masyuko, 2020_Kenya	Metabolic syndrome and 10-year cardiovascular risk among HIV-positive and HIV-negative adults: A cross-sectional study.	Cross-sectional	Non probabilistic	Consecutive sampling	Hospital-based	Monocenter	Prospectively	Kenya	Africa	Eastern Africa	Lower-middle income economies	Sep/2017-May/2018	All ages	Apparently healthy individuals	JIS, 2009
202	Masyuko, 2020_Kenya	Metabolic syndrome and 10-year cardiovascular risk among HIV-positive and HIV-negative adults: A cross-sectional study.	Cross-sectional	Non probabilistic	Consecutive sampling	Hospital-based	Monocenter	Prospectively	Kenya	Africa	Eastern Africa	Lower-middle income economies	Sep/2017-May/2018	All ages	HIV infected patients	JIS, 2009
203	Matsha, 2009_South Africa	Metabolic syndrome in 10-16-year-old learners from the Western Cape, South Africa: Comparison of the NCEP ATP III and IDF criteria.	Cross-sectional	Probabilistic	Multistage sampling	Community-based	Multicenter	Prospectively	South Africa	Africa	Southern Africa	Upper-middle-income economies	Unclear/ Not reported	Children : Birth-18 years	Apparently healthy individuals	NCEP-ATP III, 2001
204	Matsha, 2019_South Africa	Visceral and subcutaneous adipose tissue association with metabolic syndrome and its components in a South African population.	Cross-sectional	Non probabilistic	Consecutive sampling	Hospital-based	Monocenter	Prospectively	South Africa	Africa	Southern Africa	Upper-middle-income economies	Unclear/ Not reported	Adults: 18+ years	Apparently healthy individuals	JIS, 2009
205	Mbugua, 2017_Kenya	Metabolic syndrome and its components among university students in Kenya.	Cross-sectional	Probabilistic	Stratified sampling	Community-based	Monocenter	Prospectively	Kenya	Africa	Eastern Africa	Lower-middle income economies	Unclear/ Not reported	Adults: 18+ years	Apparently healthy individuals	JIS, 2009
206	Mbunkah, 2014_Cameroon	Prevalence of metabolic syndrome in human immunodeficiency virus - infected patients from the South-West region of Cameroon, using the adult treatment panel III criteria.	Cross-sectional	Non probabilistic	Consecutive sampling	Hospital-based	Multicenter	Prospectively	Cameroon	Africa	Central Africa	Lower-middle income economies	Nov/2010-Jul/2011	Adults: 18+ years	Apparently healthy individuals	NCEP-ATP III, 2001
207	Mbunkah, 2014_Cameroon	Prevalence of metabolic syndrome in human immunodeficiency virus - infected patients from the South-West region of Cameroon, using the adult treatment panel III criteria.	Cross-sectional	Non probabilistic	Consecutive sampling	Hospital-based	Multicenter	Prospectively	Cameroon	Africa	Central Africa	Lower-middle income economies	Nov/2010-Jul/2011	Adults: 18+ years	HIV infected patients	NCEP-ATP III, 2001
208	Mebazaa, 2011_Tunisia	Metabolic syndrome in Tunisian psoriatic patients: prevalence and determinants.	Case control	Non probabilistic	Consecutive sampling	Hospital-based	Monocenter	Prospectively	Tunisia	Eastern Mediterranean	Northern Africa	Lower-middle income economies	Aug/2008-Mar/2010	Unclear/Not reported	Apparently healthy individuals	NCEP-ATP III, 2001
209	Mebazaa, 2011_Tunisia	Metabolic syndrome in Tunisian psoriatic patients: prevalence and determinants.	Case control	Non probabilistic	Consecutive sampling	Hospital-based	Monocenter	Prospectively	Tunisia	Eastern Mediterranean	Northern Africa	Lower-middle income economies	Aug/2008-Mar/2010	Unclear/Not reported	Patients with dermatological diseases	NCEP-ATP III, 2001
210	Mentoor, 2018_South Africa	Metabolic syndrome and body shape predict differences in health parameters in farm working women.	Cross-sectional	Non probabilistic	Consecutive sampling	Community-based	Monocenter	Prospectively	South Africa	Africa	Southern Africa	Upper-middle-income economies	Mar/2015-Jul/2015	Adults: 18+ years	Apparently healthy individuals	IDF, 2005
211	Meziane, 2016_Morocco	Metabolic syndrome in Moroccan patients with psoriasis.	Case control	Non probabilistic	Consecutive sampling	Hospital-based	Monocenter	Prospectively	Morocco	Eastern Mediterranean	Northern Africa	Lower-middle income economies	Nov/2011-Nov/2012	All ages	Patients with dermatological diseases	IDF, 2005
212	Millogo, 2014_Burkina Faso	[Metabolic syndrome in hypertensive patients in the cardiology service Yalgado Ouedraogo of Ouagadougou, Burkina Faso].	Cross-sectional	Non probabilistic	Consecutive sampling	Hospital-based	Monocenter	Retrospectively	Burkina Faso	Africa	West Africa	Low-income economies	Jan/2011-Dec/2012	All ages	Hypertensive patients	IDF, 2005
213	Mogre, 2014_Ghana	Prevalence, components and associated demographic and lifestyle factors of the metabolic syndrome in type 2 diabetes mellitus.	Cross-sectional	Non probabilistic	Consecutive sampling	Hospital-based	Monocenter	Prospectively	Ghana	Africa	West Africa	Lower-middle income economies	Unclear/ Not reported	Adults: 18+ years	Type 2 diabetes patients	IDF, 2005
214	Mohamed, 2017_Egypt	RELATION BETWEEN SCHISTOSOME PAST INFECTION AND METABOLIC SYNDROME.	Cross-sectional	Non probabilistic	Consecutive sampling	Hospital-based	Monocenter	Prospectively	Egypt	Eastern Mediterranean	Northern Africa	Lower-middle income economies	Jun/2012-Apr/2015	Adults: 18+ years	Apparently healthy individuals	IDF, 2005
215	Mohammed el, 2008_Morocco	Prevalence of parameter indicators of obesity and its relationship with metabolic syndrome in urban Moroccan women.	Cross-sectional	Non probabilistic	Consecutive sampling	Hospital-based	Monocenter	Prospectively	Morocco	Eastern Mediterranean	Northern Africa	Lower-middle income economies	Unclear/ Not reported	Adults: 18+ years	Apparently healthy individuals	NCEP-ATP III, 2001
217	Motala, 2011_South Africa	The prevalence of metabolic syndrome and determination of the optimal waist circumference cutoff points in a rural South african community.	Cross-sectional	Probabilistic	Simple random sampling	Community-based	Multicenter	Prospectively	South Africa	Africa	Southern Africa	Upper-middle-income economies	Unclear/ Not reported	All ages	Apparently healthy individuals	JIS, 2009
218	Motuma, 2020_Ethiopia	Metabolic Syndrome Among Working Adults in Eastern Ethiopia.	Cross-sectional	Non probabilistic	Consecutive sampling	Community-based	Multicenter	Prospectively	Ethiopia	Africa	Eastern Africa	Low-income economies	Dec/2018-Feb/2019	Adults: 18+ years	Apparently healthy individuals	IDF, 2005
219	Moustakim, 2021_Morocco	Association of Metabolic Syndrome and Chronic Kidney Disease in Moroccan Adult Population.	Cross-sectional	Non probabilistic	Consecutive sampling	Community-based	Multicenter	Prospectively	Morocco	Eastern Mediterranean	Northern Africa	Lower-middle income economies	Unclear/ Not reported	Adults: 18+ years	Apparently healthy individuals	NCEP-ATP III, 2001
220	Muchanga Sifa, 2014_Democratic Republic of the Congo	Prevalence and predictors of metabolic syndrome among Congolese pre- and postmenopausal women.	Cross-sectional	Non probabilistic	Consecutive sampling	Hospital-based	Monocenter	Prospectively	Democratic Republic of the Congo	Africa	Central Africa	Low-income economies	Jan/2010-Jun/2010	Adults: 18+ years	Apparently healthy individuals	NCEP-ATP III, 2001
221	Muhammad, 2017_Nigeria	Metabolic syndrome among HIV infected patients: A comparative cross sectional study in northwestern Nigeria.	Case control	Non probabilistic	Consecutive sampling	Hospital-based	Monocenter	Prospectively	Nigeria	Africa	West Africa	Lower-middle income economies	Unclear/ Not reported	Adults: 18+ years	HIV infected patients	NCEP-ATP III, 2001
222	Mustafa, 2021_Egypt	Metabolic syndrome in androgenetic alopecia patients; Is serum regulated on activation, normal T-cell expressed and secreted the missing link?	Case control	Non probabilistic	Consecutive sampling	Hospital-based	Monocenter	Prospectively	Egypt	Eastern Mediterranean	Northern Africa	Lower-middle income economies	Unclear/ Not reported	Adults: 18+ years	Apparently healthy individuals	NCEP-ATP III, 2001
223	Mustafa, 2021_Egypt	Metabolic syndrome in androgenetic alopecia patients; Is serum regulated on activation, normal T-cell expressed and secreted the missing link?	Case control	Non probabilistic	Consecutive sampling	Hospital-based	Monocenter	Prospectively	Egypt	Eastern Mediterranean	Northern Africa	Lower-middle income economies	Unclear/ Not reported	Adults: 18+ years	Patients with dermatological diseases	NCEP-ATP III, 2001
224	Muyanja, 2016_Uganda	High Prevalence of Metabolic Syndrome and Cardiovascular Disease Risk Among People with HIV on Stable ART in Southwestern Uganda.	Cross-sectional	Non probabilistic	Consecutive sampling	Hospital-based	Monocenter	Prospectively	Uganda	Africa	Eastern Africa	Low-income economies	Unclear/ Not reported	Adults: 18+ years	HIV infected patients	Revised NCEP-ATP III, 2005
225	Nalado, 2015_Nigeria	PREVALENCE OF METABOLIC SYNDROME AMONG APPARENTLY HEALTHY ADULTS IN A RURAL COMMUNITY, IN NORTH-WESTERN NIGERIA.	Cross-sectional	Probabilistic	Multistage sampling	Community-based	Monocenter	Prospectively	Nigeria	Africa	West Africa	Lower-middle income economies	Unclear/ Not reported	Unclear/Not reported	Apparently healthy individuals	NCEP-ATP III, 2001
226	Ngatchou, 2013_Cameroon	Increased burden and severity of metabolic syndrome and arterial stiffness in treatment-naïve HIV+ patients from Cameroon.	Cross-sectional	Non probabilistic	Consecutive sampling	Hospital-based	Monocenter	Prospectively	Cameroon	Africa	Central Africa	Lower-middle income economies	Sep/2009-May/2010	Unclear/Not reported	Apparently healthy individuals	Revised NCEP-ATP III, 2005
227	Ngatchou, 2013_Cameroon	Increased burden and severity of metabolic syndrome and arterial stiffness in treatment-naïve HIV+ patients from Cameroon.	Cross-sectional	Non probabilistic	Consecutive sampling	Hospital-based	Monocenter	Prospectively	Cameroon	Africa	Central Africa	Lower-middle income economies	Sep/2009-May/2010	Unclear/Not reported	HIV infected patients	Revised NCEP-ATP III, 2005
228	Ngoude, 2021_Cameroon	Relationship between periodontal diseases and newly-diagnosed metabolic syndrome components in a sub-Saharan population: a cross sectional study.	Cross-sectional	Non probabilistic	Consecutive sampling	Hospital-based	Multicenter	Prospectively	Cameroon	Africa	Central Africa	Lower-middle income economies	Jan/2020-May/2020	Adults: 18+ years	Patients with chronic diseases	IDF, 2005
229	N'Guetta, 2016_Côte d'Ivoire	[Prevalence and characteristics of metabolic syndrome among hypertensive patients in Abidjan].	Cross-sectional	Non probabilistic	Consecutive sampling	Hospital-based	Monocenter	Prospectively	Côte d'Ivoire	Africa	West Africa	Lower-middle income economies	Nov/2014-Jul/2015	Adults: 18+ years	Hypertensive patients	IDF, 2005
230	Nguyen, 2017_South Africa	Metabolic Syndrome in People Living with Human Immunodeficiency Virus: An Assessment of the Prevalence and the Agreement between Diagnostic Criteria.	Cross-sectional	Non probabilistic	Consecutive sampling	Hospital-based	Multicenter	Prospectively	South Africa	Africa	Southern Africa	Upper-middle-income economies	Mar/2014-Feb/2015	Adults: 18+ years	HIV infected patients	JIS, 2009
231	Nibouche, 2016_Algeria	[Arterial hypertension at the time of diagnosis of type 2 diabetes in adults].	Cross-sectional	Non probabilistic	Consecutive sampling	Hospital-based	Multicenter	Prospectively	Algeria	Africa	Northern Africa	Lower-middle income economies	Unclear/ Not reported	Adults: 18+ years	Type 2 diabetes patients	NCEP-ATP III, 2001
232	Nibouche-Hattab, 2017_South Africa	Orthostatic hypertension in normotensive type 2 diabetics: What characteristics?	Cross-sectional	Non probabilistic	Consecutive sampling	Hospital-based	Monocenter	Prospectively	South Africa	Africa	Southern Africa	Upper-middle-income economies	Jan/2009-Dec/2013	Adults: 18+ years	Type 2 diabetes patients	JIS, 2009
235	Nsiah, 2015_Ghana	Prevalence of metabolic syndrome in type 2 diabetes mellitus patients.	Cross-sectional	Non probabilistic	Consecutive sampling	Hospital-based	Monocenter	Prospectively	Ghana	Africa	West Africa	Lower-middle income economies	Feb/2013-Apr/2013	Adults: 18+ years	Type 2 diabetes patients	NCEP-ATP III, 2001
236	Ntandou, 2009_Benin	Abdominal obesity explains the positive rural-urban gradient in the prevalence of the metabolic syndrome in Benin, West Africa.	Cross-sectional	Probabilistic	Simple random sampling	Community-based	Multicenter	Prospectively	Benin	Africa	West Africa	Lower-middle income economies	Unclear/ Not reported	Adults: 18+ years	Apparently healthy individuals	IDF, 2005
240	Ntyintyane, 2009_South Africa	Leptin, adiponectin, and high-sensitivity C-reactive protein in relation to the metabolic syndrome in urban South African blacks with and without coronary artery disease.	Cross-sectional	Non probabilistic	Consecutive sampling	Hospital-based	Monocenter	Prospectively	South Africa	Africa	Southern Africa	Upper-middle-income economies	Unclear/ Not reported	Unclear/Not reported	Apparently healthy individuals	IDF, 2005
241	Ntyintyane, 2009_South Africa	Leptin, adiponectin, and high-sensitivity C-reactive protein in relation to the metabolic syndrome in urban South African blacks with and without coronary artery disease.	Cross-sectional	Non probabilistic	Consecutive sampling	Hospital-based	Monocenter	Prospectively	South Africa	Africa	Southern Africa	Upper-middle-income economies	Unclear/ Not reported	Unclear/Not reported	Patients with coronary artery disease	IDF, 2005
237	Ntyintyane, 2006_South Africa	Metabolic syndrome, undiagnosed diabetes mellitus and insulin resistance are highly prevalent in urbanised South African blacks with coronary artery disease.	Cross-sectional	Non probabilistic	Consecutive sampling	Hospital-based	Monocenter	Prospectively	South Africa	Africa	Southern Africa	Upper-middle-income economies	Unclear/ Not reported	Unclear/Not reported	Patients with coronary artery disease	NCEP-ATP III, 2001
238	Ntyintyane, 2008_South Africa	Postprandial lipaemia, metabolic syndrome and LDL particle size in urbanised South African blacks with and without coronary artery disease.	Cross-sectional	Non probabilistic	Consecutive sampling	Hospital-based	Monocenter	Prospectively	South Africa	Africa	Southern Africa	Upper-middle-income economies	Unclear/ Not reported	Unclear/Not reported	Apparently healthy individuals	IDF, 2005
239	Ntyintyane, 2008_South Africa	Postprandial lipaemia, metabolic syndrome and LDL particle size in urbanised South African blacks with and without coronary artery disease.	Cross-sectional	Non probabilistic	Consecutive sampling	Hospital-based	Monocenter	Prospectively	South Africa	Africa	Southern Africa	Upper-middle-income economies	Unclear/ Not reported	Unclear/Not reported	Patients with coronary artery disease	IDF, 2005
242	Nwankwo, 2019_Nigeria	Association between high blood pressure with risk of type 2 diabetes, metabolic syndrome and its predictors: A cross-sectional study.	Cross-sectional	Non probabilistic	Consecutive sampling	Hospital-based	Monocenter	Prospectively	Nigeria	Africa	West Africa	Lower-middle income economies	Sep/2017-Aug/2018	Adults: 18+ years	Patients with chronic diseases	NCEP-ATP III, 2001

244	Obirikorang, 2018_Ghana	Association of Wrist Circumference and Waist-to-Height Ratio with Cardiometabolic Risk Factors among Type II Diabetics in a Ghanaian Population.	Cross-sectional	Non probabilistic	Convenience sampling	Hospital-based	Monocenter	Prospetively	Ghana	Africa	West Africa	Lower-middle income economies	Unclear/ Not reported	Unclear/Not reported	Apparently healthy individuals	AACE, 2003
245	Obirikorang, 2018_Ghana	Association of Wrist Circumference and Waist-to-Height Ratio with Cardiometabolic Risk Factors among Type II Diabetics in a Ghanaian Population.	Cross-sectional	Non probabilistic	Convenience sampling	Hospital-based	Monocenter	Prospetively	Ghana	Africa	West Africa	Lower-middle income economies	Unclear/ Not reported	Unclear/Not reported	Type 2 diabetes patients	IDF, 2005
243	Obirikorang, 2016_Ghana	Prevalence of metabolic syndrome among HIV-infected patients in Ghana: A cross-sectional study.	Cross-sectional	Non probabilistic	Consecutive sampling	Hospital-based	Monocenter	Prospetively	Ghana	Africa	West Africa	Lower-middle income economies	Feb/2013-Dec/2013	Adults: 18+ years	HIV infected patients	NCEP-ATP III, 2001
246	Odum, 2018_Nigeria	Elevated cardiac troponin I, creatine kinase and myoglobin and their relationship with cardiovascular risk factors in patients with type 2 diabetes.	Cross-sectional	Non probabilistic	Consecutive sampling	Hospital-based	Monocenter	Prospetively	Nigeria	Africa	West Africa	Lower-middle income economies	Unclear/ Not reported	Unclear/Not reported	Type 2 diabetes patients	Revised NCEP-ATP III, 2005
249	Ogbera, 2011_Nigeria	Menopausal symptoms and the metabolic syndrome in Nigerian women with type 2 diabetes mellitus.	Cross-sectional	Non probabilistic	Consecutive sampling	Hospital-based	Monocenter	Prospetively	Nigeria	Africa	West Africa	Lower-middle income economies	Unclear/ Not reported	Adults: 18+ years	Type 2 diabetes patients	NCEP-ATP III, 2001
247	Ogbera, 2010_Nigeria	Prevalence and gender distribution of the metabolic syndrome.	Cross-sectional	Non probabilistic	Consecutive sampling	Hospital-based	Monocenter	Prospetively	Nigeria	Africa	West Africa	Lower-middle income economies	Unclear/ Not reported	Unclear/Not reported	Type 2 diabetes patients	JIS, 2009
250	Ogbera, 2011_Nigeria	Relationship between serum testosterone levels and features of the metabolic syndrome defining criteria in patients with type 2 diabetes mellitus.	Cross-sectional	Non probabilistic	Consecutive sampling	Hospital-based	Monocenter	Prospetively	Nigeria	Africa	West Africa	Lower-middle income economies	Dec/2009-May/2010	Adults: 18+ years	Type 2 diabetes patients	JIS, 2009
248	Ogbera, 2010_Nigeria	Hyperuricaemia and the metabolic syndrome in type 2 DM.	Cross-sectional	Non probabilistic	Consecutive sampling	Hospital-based	Multicenter	Prospetively	Nigeria	Africa	West Africa	Lower-middle income economies	Nov/2008-Jan/2009	Adults: 18+ years	Type 2 diabetes patients	JIS, 2009
251	Ogbera, 2012_Nigeria	The metabolic syndrome in thyroid disease: A report from Nigeria.	Cross-sectional	Non probabilistic	Consecutive sampling	Hospital-based	Monocenter	Prospetively	Nigeria	Africa	West Africa	Lower-middle income economies	Unclear/ Not reported	All ages	Patients with thyroid disease	JIS, 2009
252	Ogbu, 2012_Nigeria	Prevalence of metabolic syndrome using weight and weight indices in an apparently healthy Nigerian population.	Cross-sectional	Non probabilistic	Consecutive sampling	Hospital-based	Monocenter	Prospetively	Nigeria	Africa	West Africa	Lower-middle income economies	Mar/2006-Sep/2006	Adults: 18+ years	Apparently healthy individuals	NCEP-ATP III, 2001
253	Ogbu, 2012_Nigeria	The Prevalence of the Metabolic Syndrome among normal weight Nigerians.	Cross-sectional	Non probabilistic	Consecutive sampling	Hospital-based	Monocenter	Prospetively	Nigeria	Africa	West Africa	Lower-middle income economies	Mar/2006-Sep/2006	Adults: 18+ years	Apparently healthy individuals	NCEP-ATP III, 2001
254	Ogedengbe, 2014_Nigeria	Profile of metabolic abnormalities seen in patients with type 2 diabetes mellitus and their first degree relatives with metabolic syndrome seen in Benin City, Edo state Nigeria.	Cross-sectional	Non probabilistic	Convenience sampling	Hospital-based	Monocenter	Prospetively	Nigeria	Africa	West Africa	Lower-middle income economies	Unclear/ Not reported	Adults: 18+ years	Apparently healthy individuals	WHO,1998
255	Ogedengbe, 2014_Nigeria	Profile of metabolic abnormalities seen in patients with type 2 diabetes mellitus and their first degree relatives with metabolic syndrome seen in Benin City, Edo state Nigeria.	Cross-sectional	Non probabilistic	Convenience sampling	Hospital-based	Monocenter	Prospetively	Nigeria	Africa	West Africa	Lower-middle income economies	Unclear/ Not reported	Adults: 18+ years	Type 2 diabetes patients	WHO,1998
256	Oguoma, 2016_Nigeria	Association between metabolic syndrome and 10-year risk of developing cardiovascular disease in a Nigerian population.	Cross-sectional	Probabilistic	Cluster sampling	Community-based	Monocenter	Prospetively	Nigeria	Africa	West Africa	Lower-middle income economies	Unclear/ Not reported	Adults: 18+ years	Apparently healthy individuals	JIS, 2009
257	Ojji, 2012_Nigeria	Prevalence of metabolic syndrome among hypertensive patients in Abuja, Nigeria.	Cross-sectional	Non probabilistic	Consecutive sampling	Hospital-based	Monocenter	Prospetively	Nigeria	Africa	West Africa	Lower-middle income economies	Unclear/ Not reported	Unclear/Not reported	Hypertensive patients	WHO,1998
258	Okpala, 2019_Nigeria	Metabolic Syndrome and Dyslipidemia among Nigerians with Lichen Planus: A Cross-Sectional Study.	Cross-sectional	Probabilistic	Simple random sampling	Hospital-based	Monocenter	Prospetively	Nigeria	Africa	West Africa	Lower-middle income economies	Dec/2014-Feb/2016	Adults: 18+ years	Apparently healthy individuals	NCEP-ATP III, 2001
259	Okpala, 2019_Nigeria	Metabolic Syndrome and Dyslipidemia among Nigerians with Lichen Planus: A Cross-Sectional Study.	Cross-sectional	Probabilistic	Simple random sampling	Hospital-based	Monocenter	Prospetively	Nigeria	Africa	West Africa	Lower-middle income economies	Dec/2014-Feb/2016	Adults: 18+ years	Patients with dermatological diseases	NCEP-ATP III, 2001
260	Okpechi, 2007_South Africa	Microalbuminuria and the metabolic syndrome in non-diabetic black Africans.	Cross-sectional	Non probabilistic	Consecutive sampling	Community-based	Monocenter	Prospetively	South Africa	Africa	Southern Africa	Upper-middle-income economies	May/2005-Jul/2006	Unclear/Not reported	Apparently healthy individuals	NCEP-ATP III, 2001
261	Okube, 2020_Kenya	Association of dietary patterns and practices on metabolic syndrome in adults with central obesity attending a mission hospital in Kenya: a cross-sectional study.	Cross-sectional	Probabilistic	Systematic sampling	Hospital-based	Monocenter	Prospetively	Kenya	Africa	Eastern Africa	Lower-middle income economies	Unclear/ Not reported	Adults: 18+ years	Patients with chronic diseases	JIS, 2009
262	Olokor, 2018_Nigeria	Prevalence of metabolic syndrome among chronic kidney disease patients attending a tertiary hospital in Nigeria – a cross - sectional study.	Cross-sectional	Non probabilistic	Consecutive sampling	Hospital-based	Monocenter	Prospetively	Nigeria	Africa	West Africa	Lower-middle income economies	Unclear/ Not reported	Adults: 18+ years	Patients with chronic kidney diseases	NCEP-ATP III, 2001
263	Omech, 2016_Botswana	Prevalence and determinants of metabolic syndrome: a cross-sectional survey of general medical outpatient clinics using National Cholesterol Education Program-Adult Treatment Panel III criteria in Botswana.	Cross-sectional	Probabilistic	Systematic sampling	Hospital-based	Multicenter	Prospetively	Botswana	Africa	Southern Africa	Upper-middle-income economies	Aug/2014-Oct/2014	Adults: 18+ years	Apparently healthy individuals	NCEP-ATP III, 2001
264	Omuse, 2017_Kenya	Metabolic syndrome and its predictors in an urban population in Kenya: A cross sectional study.	Cross-sectional	Non probabilistic	Consecutive sampling	Community-based	Multicenter	Prospetively	Kenya	Africa	Eastern Africa	Lower-middle income economies	Jan/2015-Oct/2015	Adults: 18+ years	Apparently healthy individuals	JIS, 2009
265	Onesi, 2014_Nigeria	Metabolic syndrome: Performance of five different diagnostic criterias.	Cross-sectional	Non probabilistic	Convenience sampling	Hospital-based	Multicenter	Prospetively	Nigeria	Africa	West Africa	Lower-middle income economies	Unclear/ Not reported	Adults: 18+ years	Apparently healthy individuals	IDF, 2005
266	Onesi, 2014_Nigeria	Metabolic syndrome: Performance of five different diagnostic criterias.	Cross-sectional	Non probabilistic	Convenience sampling	Hospital-based	Multicenter	Prospetively	Nigeria	Africa	West Africa	Lower-middle income economies	Unclear/ Not reported	Adults: 18+ years	Type 2 diabetes patients	WHO,1998
267	Onyekwere, 2011_Nigeria	Non-alcoholic fatty liver disease and the metabolic syndrome in an urban hospital serving an African community.	Cross-sectional	Non probabilistic	Consecutive sampling	Hospital-based	Monocenter	Prospetively	Nigeria	Africa	West Africa	Lower-middle income economies	Oct/2009-Aug/2010	Adults: 18+ years	Apparently healthy individuals	JIS, 2009
268	Onyekwere, 2011_Nigeria	Non-alcoholic fatty liver disease and the metabolic syndrome in an urban hospital serving an African community.	Cross-sectional	Non probabilistic	Consecutive sampling	Hospital-based	Monocenter	Prospetively	Nigeria	Africa	West Africa	Lower-middle income economies	Oct/2009-Aug/2010	Adults: 18+ years	Type 2 diabetes patients	JIS, 2009
269	Onyenekwu, 2017_Nigeria	Relationship between plasma osteocalcin, glycaemic control and components of metabolic syndrome in adult Nigerians with type 2 diabetes mellitus.	Cross-sectional	Non probabilistic	Consecutive sampling	Hospital-based	Multicenter	Prospetively	Nigeria	Africa	West Africa	Lower-middle income economies	Unclear/ Not reported	Adults: 18+ years	Apparently healthy individuals	IDF, 2005
270	Onyenekwu, 2017_Nigeria	Relationship between plasma osteocalcin, glycaemic control and components of metabolic syndrome in adult Nigerians with type 2 diabetes mellitus.	Cross-sectional	Non probabilistic	Consecutive sampling	Hospital-based	Multicenter	Prospetively	Nigeria	Africa	West Africa	Lower-middle income economies	Unclear/ Not reported	Adults: 18+ years	Type 2 diabetes patients	IDF, 2005
271	Onyenekwu, 2017_Nigeria	The prevalence of metabolic syndrome and its components among overweight and obese Nigerian adolescents and young adults.	Cross-sectional	Non probabilistic	Consecutive sampling	Hospital-based	Monocenter	Prospetively	Nigeria	Africa	West Africa	Lower-middle income economies	Aug/2015-Sep/2015	All ages	Type 2 diabetes patients	IDF, 2005
272	Osei-Yeboah, 2017_Ghana	The Prevalence of Metabolic Syndrome and Its Components among People with Type 2 Diabetes in the Ho Municipality, Ghana: A Cross-Sectional Study.	Cross-sectional	Non probabilistic	Convenience sampling	Hospital-based	Monocenter	Prospetively	Ghana	Africa	West Africa	Lower-middle income economies	Feb/2016-Apr/2016	Adults: 18+ years	Type 2 diabetes patients	IDF, 2005
273	Osoti, 2018_Kenya	Metabolic Syndrome Among Antiretroviral Therapy-Naive Versus Experienced HIV-Infected Patients Without Preexisting Cardiometabolic Disorders in Western Kenya.	Cross-sectional	Non probabilistic	Consecutive sampling	Hospital-based	Monocenter	Prospetively	Kenya	Africa	Eastern Africa	Lower-middle income economies	Jul/2014-Sep/2014	Adults: 18+ years	HIV infected patients	IDF, 2005
275	Osuji, 2012_Nigeria	Metabolic syndrome in newly diagnosed type 2 diabetes mellitus using NCEP-ATP III, the Nnewi experience.	Cross-sectional	Non probabilistic	Consecutive sampling	Hospital-based	Monocenter	Prospetively	Nigeria	Africa	West Africa	Lower-middle income economies	Unclear/ Not reported	Adults: 18+ years	Type 2 diabetes patients	NCEP-ATP III, 2001
274	Osuji, 2012_Nigeria	Prevalence and characteristics of the metabolic syndrome among newly diagnosed hypertensive patients.	Cross-sectional	Non probabilistic	Consecutive sampling	Hospital-based	Monocenter	Prospetively	Nigeria	Africa	West Africa	Lower-middle income economies	Unclear/ Not reported	Adults: 18+ years	Hypertensive patients	NCEP-ATP III, 2001
277	Owiredu, 2011_Ghana	The prevalence of metabolic syndrome among active sportsmen/sportswomen and sedentary workers in the Kumasi metropolis.	Cross-sectional	Non probabilistic	Consecutive sampling	Community-based	Monocenter	Prospetively	Ghana	Africa	West Africa	Lower-middle income economies	Mar/2010-Apr/2010	Adults: 18+ years	Apparently healthy individuals	IDF, 2005
278	Owiredu, 2016_Ghana	Co-existence of syndrome X and hypertension among Ghanaians.	Cross-sectional	Non probabilistic	Consecutive sampling	Hospital-based	Monocenter	Prospetively	Ghana	Africa	West Africa	Lower-middle income economies	Apr/2009-Nov/2010	Unclear/Not reported	Apparently healthy individuals	IDF, 2005
279	Owiredu, 2016_Ghana	Co-existence of syndrome X and hypertension among Ghanaians.	Cross-sectional	Non probabilistic	Consecutive sampling	Hospital-based	Monocenter	Prospetively	Ghana	Africa	West Africa	Lower-middle income economies	Apr/2009-Nov/2010	Unclear/Not reported	Hypertensive patients	IDF, 2005
280	Owusu-Ansah, 2018_Ghana	Metabolic Syndrome among Schizophrenic Patients: A Comparative Cross-Sectional Study in the Middle Belt of Ghana.	Cross-sectional	Probabilistic	Simple random sampling	Hospital-based	Monocenter	Prospetively	Ghana	Africa	West Africa	Lower-middle income economies	Apr/2016-Aug/2016	Unclear/Not reported	Psychiatric patients	IDF, 2005
281	Paquette, 2017_Kenya	Association Between Plasma Proprotein Convertase Subtilisin/Kexin Type 9 and the Presence of Metabolic Syndrome in a Predominantly Rural-Based Sub-Saharan African Population.	Cross-sectional	Probabilistic	Simple random sampling	Community-based	Multicenter	Prospetively	Kenya	Africa	Eastern Africa	Lower-middle income economies	Aug/2005-Jan/2006	Adults: 18+ years	Apparently healthy individuals	JIS, 2009
282	Paruk, 2019_South Africa	Prevalence of low serum testosterone levels among men with type 2 diabetes mellitus attending two outpatient diabetes clinics in KwaZulu-Natal Province, South Africa.	Cross-sectional	Non probabilistic	Consecutive sampling	Hospital-based	Multicenter	Prospetively	South Africa	Africa	Southern Africa	Upper-middle-income economies	Jan/2014-Mar/2015	Adults: 18+ years	Type 2 diabetes patients	JIS, 2009
283	Peer, 2016_South Africa	Differential obesity indices identify the metabolic syndrome in Black men and women in Cape Town: the CRIBSA study.	Cross-sectional	Probabilistic	Cluster sampling	Community-based	Multicenter	Prospetively	South Africa	Africa	Southern Africa	Upper-middle-income economies	2008-2009	Adults: 18+ years	Apparently healthy individuals	JIS, 2009
284	Pengpid, 2020_Morocco	Prevalence and correlates of the metabolic syndrome in a cross-sectional community-based sample of 18-100 year-olds in Morocco: Results of the first national STEPS survey in 2017.	Cross-sectional	Probabilistic	Multistage sampling	Community-based	Nationally representative	Prospetively	Morocco	Eastern Mediterranean	Northern Africa	Lower-middle income economies	2017	Adults: 18+ years	Apparently healthy individuals	JIS, 2009
285	Pessinaba, 2013_Senegal	[Prevalence survey of cardiovascular risk factors in the general population in St. Louis (Senegal)].	Cross-sectional	Non probabilistic	Consecutive sampling	Community-based	Monocenter	Prospetively	Senegal	Africa	West Africa	Lower-middle income economies	Unclear/ Not reported	All ages	Apparently healthy individuals	Revised NCEP-ATP III, 2005
286	Phalane, 2018_South Africa	The metabolic syndrome and renal function in an African cohort infected with human immunodeficiency virus.	Cohort (Baseline data)	Non probabilistic	Consecutive sampling	Community-based	Multicenter	Prospetively	South Africa	Africa	Southern Africa	Upper-middle-income economies	Unclear/ Not reported	Adults: 18+ years	Apparently healthy individuals	IDF, 2005
287	Phalane, 2018_South Africa	The metabolic syndrome and renal function in an African cohort infected with human immunodeficiency virus.	Cohort (Baseline data)	Non probabilistic	Consecutive sampling	Community-based	Multicenter	Prospetively	South Africa	Africa	Southern Africa	Upper-middle-income economies	Unclear/ Not reported	Adults: 18+ years	HIV infected patients	IDF, 2005
288	Quaye, 2019_Ghana	Comparative Abilities of Body Mass Index, Waist Circumference, Abdominal Volume Index, Body Adiposity Index, and Conicity Index as Predictive Screening Tools for Metabolic Syndrome among Apparently Healthy Ghanaian Adults.	Cross-sectional	Non probabilistic	Consecutive sampling	Community-based	Monocenter	Prospetively	Ghana	Africa	West Africa	Lower-middle income economies	Sep/2017-Jan/2018	Adults: 18+ years	Apparently healthy individuals	JIS, 2009
289	Raharinavalona, 2020_Madagascar	[Prevalences of metabolic syndrome and cardiovascular risk factors in type 2 diabetics hospitalized in the Department of Endocrinology, Antananarivo].	Cross-sectional	Non probabilistic	Consecutive sampling	Hospital-based	Monocenter	Prospetively	Madagascar	Africa	Eastern Africa	Low-income economies	Nov/2016-May/2017	Adults: 18+ years	Type 2 diabetes patients	JIS, 2009
290	Regaieg, 2018_Tunisia	Metabolic syndrome and physical activity measured by pedometer among adolescents.	Cross-sectional	Non probabilistic	Consecutive sampling	Community-based	Monocenter	Prospetively	Tunisia	Eastern Mediterranean	Northern Africa	Lower-middle income economies	Dec/2012-Oct/2013	Children : Birth-18 years	Apparently healthy individuals	IDF, 2005
291	Rejeb, 2010_Tunisia	Metabolic syndrome is a risk factor for coronary artery disease in a tunisian population.	Cross-sectional	Non probabilistic	Consecutive sampling	Hospital-based	Monocenter	Prospetively	Tunisia	Eastern Mediterranean	Northern Africa	Lower-middle income economies	Mar/2007-Jun/2007	Adults: 18+ years	Patients with coronary artery disease	IDF, 2005
292	Rguibi, 2004_Morocco	Metabolic syndrome among Moroccan Sahraoui adult Women.	Cross-sectional	Probabilistic	Simple random sampling	Hospital-based	Monocenter	Prospetively	Morocco	Eastern Mediterranean	Northern Africa	Lower-middle income economies	Oct/2001-Apr/2002	All ages	Apparently healthy individuals	NCEP-ATP III, 2001
293	Rguibi, 2007_Morocco	High blood pressure in urban Moroccan Sahraoui women.	Cross-sectional	Probabilistic	Simple random sampling	Hospital-based	Multicenter	Prospetively	Morocco	Eastern Mediterranean	Northern Africa	Lower-middle income economies	Oct/2001-Apr/2002	Adults: 18+ years	Apparently healthy individuals	NCEP-ATP III, 2001
294	Rguibi, 2007_Morocco	High blood pressure in urban Moroccan Sahraoui women.	Cross-sectional	Probabilistic	Simple random sampling	Hospital-based	Multicenter	Prospetively	Morocco	Eastern Mediterranean	Northern Africa	Lower-middle income economies	Oct/2001-Apr/2002	Adults: 18+ years	Hypertensive patients	NCEP-ATP III, 2001
295	Rostom, 2013_Morocco	Metabolic syndrome in rheumatoid arthritis: case control study.	Case control	Non probabilistic	Consecutive sampling	Hospital-based	Monocenter	Prospetively	Morocco	Eastern Mediterranean	Northern Africa	Lower-middle income economies	May/2010-Jun/2011	Unclear/Not reported	Apparently healthy individuals	IDF, 2005
296	Rostom, 2013_Morocco	Metabolic syndrome in rheumatoid arthritis: case control study.	Case control	Non probabilistic	Consecutive sampling	Hospital-based	Monocenter	Prospetively	Morocco	Eastern Mediterranean	Northern Africa	Lower-middle income economies	May/2010-Jun/2011	Unclear/Not reported	Patients with rheumatoid arthritis	IDF, 2005
297	Rwegerera, 2021_Botswana	Metabolic Control and Determinants Among HIV-Infected Type 2 Diabetes Mellitus Patients Attending a Tertiary Clinic in Botswana.	Case control	Non probabilistic	Consecutive sampling	Hospital-based	Multicenter	Prospetively	Botswana	Africa	Southern Africa	Upper-middle-income economies	Jun/2019-Oct/2019	Unclear/Not reported	Apparently healthy individuals	IDF, 2005
298	Rwegerera, 2021_Botswana	Metabolic Control and Determinants Among HIV-Infected Type 2 Diabetes Mellitus Patients Attending a Tertiary Clinic in Botswana.	Case control	Non probabilistic	Consecutive sampling	Hospital-based	Multicenter	Prospetively	Botswana	Africa	Southern Africa	Upper-middle-income economies	Jun/2019-Oct/2019	Unclear/Not reported	HIV infected patients	IDF, 2005
299	Sabir, 2016_Nigeria	Oxidative stress among subjects with metabolic syndrome in Sokoto, North.Western Nigeria.	Cross-sectional	Probabilistic	Cluster sampling	Hospital-based	Multicenter	Prospetively	Nigeria	Africa	West Africa	Lower-middle income economies	Unclear/ Not reported	Adults: 18+ years	Apparently healthy individuals	NCEP-ATP III, 2001
300	Sabir, 2016_Nigeria	Metabolic syndrome in urban city of North-Western Nigeria: prevalence and determinants.	Cross-sectional	Probabilistic	Multistage sampling	Hospital-based	Multicenter	Prospetively	Nigeria	Africa	West Africa	Lower-middle income economies	Unclear/ Not reported	Adults: 18+ years	Apparently healthy individuals	NCEP-ATP III, 2001
301	Salawu, 2015_Nigeria	Prevalence of metabolic syndrome and its component traits among students in a Nigerian university.	Cross-sectional	Non probabilistic	Consecutive sampling	Hospital-based	Monocenter	Prospetively	Nigeria	Africa	West Africa	Lower-middle income economies	Unclear/ Not reported	Adults: 18+ years	Apparently healthy individuals	JIS, 2009
302	Salem, 2021_Egypt	Association between Toxoplasma gondii infection and metabolic syndrome in obese adolescents: A possible immune-metabolic link.	Cross-sectional	Non probabilistic	Consecutive sampling	Hospital-based	Monocenter	Prospetively	Egypt	Eastern Mediterranean	Northern Africa	Lower-middle income economies	May/2019-Oct:2019	Children : Birth-18 years	Patients with obesity	IDF, 2005
303	Saloojee, 2018_South Africa	Metabolic syndrome in antipsychotic naive African patients with severe mental illness in usual care.	Cohort (Baseline data)	Non probabilistic	Consecutive sampling	Hospital-based	Monocenter	Prospetively	South Africa	Africa	Southern Africa	Upper-middle-income economies	Jan/2012-Dec/2015	Adults: 18+ years	Apparently healthy individuals	JIS, 2009
304	Saloojee, 2018_South Africa	Metabolic syndrome in antipsychotic naive African patients with severe mental illness in usual care.	Cohort (Baseline data)	Non probabilistic	Consecutive sampling	Hospital-based	Monocenter	Prospetively	South Africa	Africa	Southern Africa	Upper-middle-income economies	Jan/2012-Dec/2015	Adults: 18+ years	Psychiatric patients	JIS, 2009
306	Sanad, 2011_Egypt	Evaluation of microalbuminuria in obese children and its relation to metabolic syndrome.	Cross-sectional	Non probabilistic	Consecutive sampling	Hospital-based	Monocenter	Prospetively	Egypt	Eastern Mediterranean	Northern Africa	Lower-middle income economies	Jan/2008-Dec/2010	Children : Birth-18 years	Patients with obesity	NCEP-ATP III, 2001
307	Sawadogo, 2014_Burkina Faso	[Metabolic syndrome and cardiovascular risk patients under antiretrovirals in a day hospital at Bobo-Dioulasso (Burkina Faso)].	Cross-sectional	Non probabilistic	Consecutive sampling	Hospital-based	Monocenter	Prospetively	Burkina Faso	Africa	West Africa	Low-income economies	2011	Adults: 18+ years	HIV infected patients	NCEP-ATP III, 2001
308	Schutte, 2007_South Africa	Metabolic syndrome risk in black South African women compared to Caucasian women.	Cross-sectional	Non probabilistic	Consecutive sampling	Community-based	Monocenter	Prospetively	South Africa	Africa	Southern Africa	Upper-middle-income economies	Mar/2003-Oct/2004	Adults: 18+ years	Apparently healthy individuals	IDF, 2005
309	Schutte, 2009_South Africa	Classifying Africans with the metabolic syndrome.	Cross-sectional	Non probabilistic	Consecutive sampling	Community-based	Multicenter	Prospetively	South Africa	Africa	Southern Africa	Upper-middle-income economies	Unclear/ Not reported	Adults: 18+ years	Apparently healthy individuals	AACE, 2003
310	Sekgala, 2018_South Africa	The risk of metabolic syndrome as a result of lifestyle among Ellisras rural young adults.	Cross-sectional	Non probabilistic	Consecutive sampling	Community-based	Monocenter	Prospetively	South Africa	Africa	Southern Africa	Upper-middle-income economies	Unclear/ Not reported	Adults: 18+ years	Apparently healthy individuals	JIS, 2009
311	Setroame, 2020_Ghana	Prevalence of Metabolic Syndrome and Nonalcoholic Fatty Liver Disease among Premenopausal and Postmenopausal Women in Ho Municipality: A Cross-Sectional Study.	Cross-sectional	Non probabilistic	Consecutive sampling	Hospital-based	Multicenter	Prospetively	Ghana	Africa	West Africa	Lower-middle income economies	Nov/2018-Jan/2019	Adults: 18+ years	Apparently healthy individuals	NCEP-ATP III, 2001
312	Setroame, 2020_Ghana	Prevalence of Metabolic Syndrome and Nonalcoholic Fatty Liver Disease among Premenopausal and Postmenopausal Women in Ho Municipality: A Cross-Sectional Study.	Cross-sectional	Non probabilistic	Consecutive sampling	Hospital-based	Multicenter	Prospetively	Ghana	Africa	West Africa	Lower-middle income economies	Nov/2018-Jan/2019	Adults: 18+ years	Postmenopausal women	NCEP-ATP III, 2001
313	Shaheen, 2012_Egypt	Assessment of serum leptin, insulin resistance and metabolic syndrome in patients with skin tags.	Case control	Probabilistic	Simple random sampling	Hospital-based	Monocenter	Prospetively	Egypt	Eastern Mediterranean	Northern Africa	Lower-middle income economies	Jan/2010-May/2011	Unclear/Not reported	Apparently healthy individuals	Revised NCEP-ATP III, 2005
314	Shaheen, 2012_Egypt	Assessment of serum leptin, insulin resistance and metabolic syndrome in patients with skin tags.	Case control	Probabilistic	Simple random sampling	Hospital-based	Monocenter	Prospetively	Egypt	Eastern Mediterranean	Northern Africa	Lower-middle income economies	Jan/2010-May/2011	Unclear/Not reported	Patients with dermatological diseases	Revised NCEP-ATP III, 2005
315	Siminialayi, 2008_Nigeria	Metabolic Syndrome in a Rural Nigerian Community: Is Central Obesity always the key Determinant?	Cross-sectional	Non probabilistic	Consecutive sampling	Community-based	Monocenter	Prospetively	Nigeria	Africa	West Africa	Lower-middle income economies	2008	Adults: 18+ years	Apparently healthy individuals	NCEP-ATP III, 2001
316	Slimani, 2017_Algeria	Prevalence of metabolic syndrome in Algerian rheumatoid arthritis patients. Correlation with disease activity and functional status.	Cross-sectional	Non probabilistic	Consecutive sampling	Hospital-based	Multicenter	Prospetively	Algeria	Africa	Northern Africa	Lower-middle income economies	Dec/2010-Apr/2011	Adults: 18+ years	Patients with rheumatoid arthritis	NCEP-ATP III, 2001
317	Soliman, 2019_Egypt	The prevalence and the clinical profile of metabolic syndrome in children and adolescents with Type 1 diabetes.	Cross-sectional	Non probabilistic	Consecutive sampling	Hospital-based	Monocenter	Prospetively	Egypt	Eastern Mediterranean	Northern Africa	Lower-middle income economies	Jul/2017-Jun/2018	Children : Birth-18 years	Patients with type 1 diabetes	IDF, 2005
318	Solomon, 2019_Ethiopia	Disease burden and associated risk factors for metabolic syndrome among adults in Ethiopia.	Cross-sectional	Non probabilistic	Consecutive sampling	Hospital-based	Monocenter	Prospetively	Ethiopia	Africa	Eastern Africa	Low-income economies	Sep/2017-Oct/2017	Adults: 18+ years	Patients with chronic diseases	Revised NCEP-ATP III, 2005

319	Sossa, 2016_Benin	Performances comparées du HDL-cholestérol et du ratio cholestérol total/HDL pour le dépistage du syndrome métabolique chez des adultes du Sud-Bénin (Afrique de l'Ouest).	Cross-sectional	Probabilistic	Cluster sampling	Community-based	Monocenter	Prospetively	Benin	Africa	West Africa	Lower-middle income economies	Unclear/ Not reported	Adults: 18+ years	Apparently healthy individuals	JIS, 2009
320	Tachebele, 2014_Ethiopia	Metabolic syndrome among hypertensive patients at University of Gondar Hospital, North West Ethiopia: a cross sectional study.	Cross-sectional	Probabilistic	Simple random sampling	Hospital-based	Monocenter	Prospetively	Ethiopia	Africa	Eastern Africa	Low-income economies	Jul/2013-Aug/2013	Adults: 18+ years	Hypertensive patients	Revised NCEP-ATP III, 2005
321	Tadewos, 2017_Ethiopia	Risk factors of metabolic syndrome among hypertensive patients at Hawassa University Comprehensive Specialized Hospital, Southern Ethiopia.	Cross-sectional	Probabilistic	Simple random sampling	Hospital-based	Monocenter	Prospetively	Ethiopia	Africa	Eastern Africa	Low-income economies	Sep/2015-Jun/2016	Adults: 18+ years	Hypertensive patients	NCEP-ATP III, 2001
322	Tesfaye, 2014_Ethiopia	Burden of metabolic syndrome among HIV-infected patients in Southern Ethiopia.	Cross-sectional	Probabilistic	Simple random sampling	Hospital-based	Monocenter	Prospetively	Ethiopia	Africa	Eastern Africa	Low-income economies	Feb/2013-Apr/2013	Adults: 18+ years	HIV infected patients	IDF, 2005
323	Teshome, 2020_Ethiopia	Prevalence and Associated Factors of Metabolic Syndrome Among Patients with Severe Mental Illness at Hawassa, Southern-Ethiopia.	Cross-sectional	Probabilistic	Systematic sampling	Hospital-based	Monocenter	Prospetively	Ethiopia	Africa	Eastern Africa	Low-income economies	Jan/2019-Jun/2019	Adults: 18+ years	Psychiatric patients	IDF, 2005
324	Titty, 2009_Ghana	Incidence and Major Metabolic Risk Factors of Metabolic Syndrome in Type 2 Diabetic Out-Patients Visiting Tamale Teaching Hospital in Ghana.	Cross-sectional	Non probabilistic	Consecutive sampling	Hospital-based	Monocenter	Prospetively	Ghana	Africa	West Africa	Lower-middle income economies	Jun/2006-May/2007	Adults: 18+ years	Type 2 diabetes patients	NCEP-ATP III, 2001
325	Titty, 2010_Ghana	Glycaemic control, dyslipidaemia and metabolic syndrome among recently diagnosed diabetes mellitus patients in Tamale Teaching Hospital, Ghana.	Cross-sectional	Non probabilistic	Consecutive sampling	Hospital-based	Monocenter	Prospetively	Ghana	Africa	West Africa	Lower-middle income economies	Sep/2006-Aug/2007	All ages	Type 2 diabetes patients	NCEP-ATP III, 2001
326	Tladi, 2021_Botswana	Prevalence of the Metabolic Syndrome Among Batswana Adults in Urban and Semi-Urban Gaborone.	Cross-sectional	Probabilistic	Cluster sampling	Hospital-based	Monocenter	Prospetively	Botswana	Africa	Southern Africa	Upper-middle-income economies	Unclear/ Not reported	Adults: 18+ years	Apparently healthy individuals	IDF, 2005
327	Tran, 2011_Ethiopia	Prevalence of Metabolic Syndrome among Working Adults in Ethiopia.	Cross-sectional	Probabilistic	Multistage sampling	Community-based	Multicenter	Prospetively	Ethiopia	Africa	Eastern Africa	Low-income economies	Dec/2009-Jan/2010	All ages	Apparently healthy individuals	IDF, 2005
328	Udenze, 2013_Nigeria	The prevalence of metabolic syndrome in persons with type 2 diabetes at the Lagos University Tteaching Hospital, Lagos, Nigeria.	Cross-sectional	Non probabilistic	Consecutive sampling	Hospital-based	Monocenter	Prospetively	Nigeria	Africa	West Africa	Lower-middle income economies	Unclear/ Not reported	Adults: 18+ years	Type 2 diabetes patients	WHO,1998
329	Ulasi, 2010_Nigeria	A community-based study of hypertension and cardio-metabolic syndrome in semi-urban and rural communities in Nigeria.	Cross-sectional	Probabilistic	Simple random sampling	Community-based	Monocenter	Prospetively	Nigeria	Africa	West Africa	Lower-middle income economies	Unclear/ Not reported	Adults: 18+ years	Apparently healthy individuals	IDF, 2005
330	Unadike, 2009_Nigeria	Prevalence of the metabolic syndrome among patients with type 2 diabetes mellitus in Uyo, Nigeria.	Cross-sectional	Non probabilistic	Consecutive sampling	Hospital-based	Monocenter	Prospetively	Nigeria	Africa	West Africa	Lower-middle income economies	Jan/2008-Aug/2008	All ages	Type 2 diabetes patients	NCEP-ATP III, 2001
331	Uwanuruochi, 2013_Nigeria	Cardiovascular risk factors in adult staff of Federal Medical Centre, Umuahia: a comparison with other Nigerian studies.	Cross-sectional	Non probabilistic	Consecutive sampling	Hospital-based	Monocenter	Prospetively	Nigeria	Africa	West Africa	Lower-middle income economies	Oct/2010	Adults: 18+ years	Apparently healthy individuals	IDF, 2005
334	Wahab, 2008_Nigeria	Frequency and determinants of the metabolic syndrome in apparently healthy adult Nigerians.	Cross-sectional	Non probabilistic	Consecutive sampling	Hospital-based	Monocenter	Prospetively	Nigeria	Africa	West Africa	Lower-middle income economies	May/2006-Jun/2006	Adults: 18+ years	Apparently healthy individuals	NCEP-ATP III, 2001
337	Workalemahu, 2013_Ethiopia	Physical activity and metabolic syndrome among Ethiopian adults.	Cross-sectional	Probabilistic	Multistage sampling	Community-based	Monocenter	Prospetively	Ethiopia	Africa	Eastern Africa	Low-income economies	Dec/2009-Jan/2010	Adults: 18+ years	Apparently healthy individuals	IDF, 2005
338	Woyesa, 2017_Ethiopia	Hyperuricemia and metabolic syndrome in type 2 diabetes mellitus patients at Hawassa university comprehensive specialized hospital, South West Ethiopia.	Cross-sectional	Non probabilistic	Consecutive sampling	Hospital-based	Monocenter	Prospetively	Ethiopia	Africa	Eastern Africa	Low-income economies	Feb/2017-May/2017	Unclear/Not reported	Type 2 diabetes patients	NCEP-ATP III, 2001
339	Yasir, 2016_Sudan	Metabolic syndrome and its association with obesity and lifestyle factors in Sudanese population.	Cross-sectional	Probabilistic	Simple random sampling	Community-based	Monocenter	Prospetively	Sudan	Eastern Mediterranean	Northern Africa	Low-income economies	2010-2012	Adults: 18+ years	Apparently healthy individuals	NCEP-ATP III, 2001
340	Yeboah, 2017_Ghana	Metabolic syndrome and parental history of cardiovascular disease in young adults in urban Ghana.	Cross-sectional	Non probabilistic	Consecutive sampling	Community-based	Monocenter	Prospetively	Ghana	Africa	West Africa	Lower-middle income economies	Nov/2015-May/2016	Adults: 18+ years	Apparently healthy individuals	JIS, 2009
341	Yerima, 2017_Nigeria	Knee osteoarthritis and associated cardio-metabolic clusters in a tertiary hospital in Nigeria.	Cross-sectional	Non probabilistic	Consecutive sampling	Hospital-based	Monocenter	Prospetively	Nigeria	Africa	West Africa	Lower-middle income economies	Mar/2015-Oct/2015	Adults: 18+ years	Patients with rheumatoid arthritis	IDF, 2005
342	Young, 2016_Nigeria	Insulin resistance, metabolic syndrome, and lipids in African women.	Cross-sectional	Non probabilistic	Consecutive sampling	Community-based	Monocenter	Prospetively	Nigeria	Africa	West Africa	Lower-middle income economies	Unclear/ Not reported	Adults: 18+ years	Apparently healthy individuals	JIS, 2009
343	Zaki, 2015_Egypt	Indicators of the metabolic syndrome in obese adolescents.	Cross-sectional	Non probabilistic	Consecutive sampling	Community-based	Monocenter	Prospetively	Egypt	Eastern Mediterranean	Northern Africa	Lower-middle income economies	Jun/2011-Jul/2012	Adults: 18+ years	Patients with obesity	IDF, 2005
344	Zeba, 2012_Burkina Faso	The double burden of malnutrition and cardiometabolic risk widens the gender and socio-economic health gap: a study among adults in Burkina Faso (West Africa).	Cross-sectional	Probabilistic	Simple random sampling	Community-based	Monocenter	Prospetively	Burkina Faso	Africa	West Africa	Low-income economies	2010	Adults: 18+ years	Apparently healthy individuals	WHO,1998
345	Zerga, 2020_Ethiopia	Metabolic syndrome and lifestyle factors among type 2 diabetes mellitus patients in Dessie Referral Hospital, Amhara region, Ethiopia.	Cross-sectional	Non probabilistic	Consecutive sampling	Hospital-based	Monocenter	Prospetively	Ethiopia	Africa	Eastern Africa	Low-income economies	Feb/2017-Mar/2017	Adults: 18+ years	Type 2 diabetes patients	JIS, 2009
1	Abagre, 2022_Ghana	Determinants of metabolic syndrome among patients attending diabetes clinics in two sub-urban hospitals: Bono Region, Ghana.	Cross-sectional	Non probabilistic	Consecutive sampling	Hospital-based	Multicenter	Prospetively	Ghana	Africa	West Africa	Lower-middle income economies	Unclear/ Not reported	Adults: 18+ years	Type 2 diabetes patients	JIS, 2009
4	Abebe, 2021_Ethiopia	Magnitude of metabolic syndrome in Gondar town, Northwest Ethiopia: A community-based cross-sectional study.	Cross-sectional	Non probabilistic	Consecutive sampling	Community-based	Multicenter	Prospetively	Ethiopia	Africa	Eastern Africa	Low-income economies	2018-2019	Adults: 18+ years	Apparently healthy individuals	IDF, 2005
8	Achila, 2022_Eritrea	Metabolic syndrome, associated factors and optimal waist circumference cut points: findings from a cross-sectional community-based study in the elderly population in Asmara, Eritrea.	Cross-sectional	Non probabilistic	Consecutive sampling	Community-based	Multicenter	Prospetively	Eritrea	Africa	Eastern Africa	Low-income economies	2018	Adults: 18+ years	Apparently healthy individuals	JIS, 2009
26	Akinyemiju, 2022_Nigeria	Metabolic Syndrome and Risk of Breast Cancer by Molecular Subtype: Analysis of the MEND Study.	Cross-sectional	Non probabilistic	Consecutive sampling	Hospital-based	Multicenter	Prospetively	Nigeria	Africa	West Africa	Lower-middle income economies	2015-2019	Adults: 18+ years	Apparently healthy individuals	JIS, 2009
27	Akinyemiju, 2022_Nigeria	Metabolic Syndrome and Risk of Breast Cancer by Molecular Subtype: Analysis of the MEND Study.	Cross-sectional	Non probabilistic	Consecutive sampling	Hospital-based	Multicenter	Prospetively	Nigeria	Africa	West Africa	Lower-middle income economies	2015-2019	Adults: 18+ years	Patients with chronic diseases	JIS, 2009
67	Bennouar, 2021_Algeria	Association and interaction between vitamin D level and metabolic syndrome for non-alcoholic fatty liver disease.	Cross-sectional	Non probabilistic	Consecutive sampling	Hospital-based	Monocenter	Prospetively	Algeria	Africa	Northern Africa	Lower-middle income economies	Jan/2019-Jan/2020	Adults: 18+ years	Apparently healthy individuals	NCEP-ATP III, 2001
68	Bennouar, 2021_Algeria	Association and interaction between vitamin D level and metabolic syndrome for non-alcoholic fatty liver disease.	Cross-sectional	Non probabilistic	Consecutive sampling	Hospital-based	Monocenter	Prospetively	Algeria	Africa	Northern Africa	Lower-middle income economies	Jan/2019-Jan/2020	Adults: 18+ years	Patients with nonalcoholic fatty liver disease	NCEP-ATP III, 2001
75	Bojang, 2021_Gambia	Prevalence of Metabolic Syndrome and its Components in Kanifing Municipality, The Gambia.	Cross-sectional	Non probabilistic	Consecutive sampling	Hospital-based	Monocenter	Prospetively	Gambia	Africa	West Africa	Low-income economies	Nov/2019-Feb/2020	Adults: 18+ years	Apparently healthy individuals	JIS, 2009
84	Challa, 2021_Ethiopia	Prevalence of metabolic syndrome among patients with schizophrenia in Ethiopia.	Cross-sectional	Non probabilistic	Consecutive sampling	Hospital-based	Monocenter	Prospetively	Ethiopia	Africa	Eastern Africa	Low-income economies	Jan/2015-Nov/2016	Unclear/Not reported	Psychiatric patients	Revised NCEP-ATP III, 2005
87	Christian, 2021_Ghana	Metabolic syndrome among individuals living with hypertension in Accra, Ghana.	Cross-sectional	Probabilistic	Simple random sampling	Community-based	Multicenter	Prospetively	Ghana	Africa	West Africa	Lower-middle income economies	2010-2013	Unclear/Not reported	Hypertensive patients	IDF, 2005
112	Farhane, 2021_Morocco	COVID-19 pandemic: Effects of national lockdown on the state of health of patients with type 2 diabetes mellitus in a Moroccan population.	Cross-sectional	Non probabilistic	Consecutive sampling	Hospital-based	Monocenter	Prospetively	Morocco	Eastern Mediterranean	Northern Africa	Lower-middle income economies	Nov/2019-Dec/2020	Adults: 18+ years	Type 2 diabetes patients	IDF, 2005
113	Fentie, 2021_Ethiopia	Metabolic syndrome and associated factors among severely ill psychiatric and non-psychiatric patients: a comparative cross-sectional study in Eastern Ethiopia.	Cross-sectional	Non probabilistic	Consecutive sampling	Hospital-based	Monocenter	Prospetively	Ethiopia	Africa	Eastern Africa	Low-income economies	2021	Adults: 18+ years	Apparently healthy individuals	Revised NCEP-ATP III, 2005
114	Fentie, 2021_Ethiopia	Metabolic syndrome and associated factors among severely ill psychiatric and non-psychiatric patients: a comparative cross-sectional study in Eastern Ethiopia.	Cross-sectional	Non probabilistic	Consecutive sampling	Hospital-based	Monocenter	Prospetively	Ethiopia	Africa	Eastern Africa	Low-income economies	2021	Adults: 18+ years	Psychiatric patients	Revised NCEP-ATP III, 2005
127	Gebreyesus, 2022_Ethiopia	High atherogenic risk concomitant with elevated HbA1c among persons with type 2 diabetes mellitus in North Ethiopia.	Cross-sectional	Non probabilistic	Consecutive sampling	Hospital-based	Multicenter	Prospetively	Ethiopia	Africa	Eastern Africa	Low-income economies	2019	Adults: 18+ years	Type 2 diabetes patients	NCEP-ATP III, 2001
130	Geto, 2021_Ethiopia	Cardiometabolic syndrome and associated factors among Ethiopian public servants, Addis Ababa, Ethiopia.	Cross-sectional	Non probabilistic	Consecutive sampling	Community-based	Multicenter	Prospetively	Ethiopia	Africa	Eastern Africa	Low-income economies	Mar/2018-Jun/2018	Adults: 18+ years	Apparently healthy individuals	IDF, 2005
138	Hamed, 2022_Egypt	Androgenetic Alopecia and Metabolic Syndrome: Is Alarin a Missing Link?	Case control	Non probabilistic	Consecutive sampling	Hospital-based	Monocenter	Prospetively	Egypt	Eastern Mediterranean	Northern Africa	Lower-middle income economies	Unclear/ Not reported	Adults: 18+ years	Apparently healthy individuals	IDF, 2005
139	Hamed, 2022_Egypt	Androgenetic Alopecia and Metabolic Syndrome: Is Alarin a Missing Link?	Case control	Non probabilistic	Consecutive sampling	Hospital-based	Monocenter	Prospetively	Egypt	Eastern Mediterranean	Northern Africa	Lower-middle income economies	Unclear/ Not reported	Adults: 18+ years	Patients with dermatological diseases	IDF, 2005
143	Hanley, 2021_South Africa	Obesity in young South African women living with HIV: A cross-sectional analysis of risk factors for cardiovascular disease.	Cross-sectional	Non probabilistic	Consecutive sampling	Hospital-based	Multicenter	Prospetively	South Africa	Africa	Southern Africa	Upper-middle-income economies	Nov/2018-May/2019	Adults: 18+ years	HIV infected patients	JIS, 2009
146	Harraqui, 2022_Morocco	Frequency of Metabolic Syndrome and Study of Anthropometric, Clinical and Biological Characteristics in Peri- and Postmenopausal Women in the City of Ksar El Kebir (Northern Morocco).	Cross-sectional	Non probabilistic	Consecutive sampling	Hospital-based	Monocenter	Prospetively	Morocco	Eastern Mediterranean	Northern Africa	Lower-middle income economies	Jan/2019-Feb/2020	Adults: 18+ years	Postmenopausal women	NCEP-ATP III, 2001
162	Jaballah, 2021_Tunisia	The Relationship Between Menopause and Metabolic Syndrome: Experimental and Bioinformatics Analysis.	Cross-sectional	Non probabilistic	Consecutive sampling	Hospital-based	Monocenter	Prospetively	Tunisia	Eastern Mediterranean	Northern Africa	Lower-middle income economies	Jan/2019-Dec/2019	Adults: 18+ years	Apparently healthy individuals	NCEP-ATP III, 2001
163	Jaballah, 2021_Tunisia	The Relationship Between Menopause and Metabolic Syndrome: Experimental and Bioinformatics Analysis.	Cross-sectional	Non probabilistic	Consecutive sampling	Hospital-based	Monocenter	Prospetively	Tunisia	Eastern Mediterranean	Northern Africa	Lower-middle income economies	Jan/2019-Dec/2019	Adults: 18+ years	Postmenopausal women	NCEP-ATP III, 2001
216	Mokwena, 2022_South Africa	Pre-morbid cardiometabolic risks among South Africans living in informal settlements.	Cross-sectional	Non probabilistic	Consecutive sampling	Community-based	Monocenter	Prospetively	South Africa	Africa	Southern Africa	Upper-middle-income economies	Oct/2016-Apr/2017	Adults: 18+ years	Apparently healthy individuals	JIS, 2009
233	Nicolaou, 2022_South Africa	Cardiometabolic outcomes of women exposed to hyperglycaemia first detected in pregnancy at 3-6 years post-partum in an urban South African setting.	Cross-sectional	Non probabilistic	Consecutive sampling	Hospital-based	Monocenter	Prospetively	South Africa	Africa	Southern Africa	Upper-middle-income economies	2019	Unclear/Not reported	Apparently healthy individuals	JIS, 2009
234	Nicolaou, 2022_South Africa	Cardiometabolic outcomes of women exposed to hyperglycaemia first detected in pregnancy at 3-6 years post-partum in an urban South African setting.	Cross-sectional	Non probabilistic	Consecutive sampling	Hospital-based	Monocenter	Prospetively	South Africa	Africa	Southern Africa	Upper-middle-income economies	2019	Unclear/Not reported	Type 2 diabetes patients	JIS, 2009
276	Osunkwo, 2022_Nigeria	Prevalence and Predictors of Metabolic Syndrome among Adults in North-Central, Nigeria.	Cross-sectional	Non probabilistic	Consecutive sampling	Community-based	Multicenter	Prospetively	Nigeria	Africa	West Africa	Lower-middle income economies	Unclear/ Not reported	Unclear/Not reported	Apparently healthy individuals	NCEP-ATP III, 2001
305	Samaan, 2022_Egypt	The Impact of Metabolic Syndrome on Quality of Life Among Individuals With Knee Osteoarthritis Living in Egypt.	Cross-sectional	Non probabilistic	Consecutive sampling	Hospital-based	Monocenter	Prospetively	Egypt	Eastern Mediterranean	Northern Africa	Lower-middle income economies	Unclear/ Not reported	Adults: 18+ years	Patients with Chronic musculoskeletal diseases	JIS, 2009
332	van der Heijden, 2021_Ghana	The Magnitude and Directions of the Associations between Early Life Factors and Metabolic Syndrome Differ across Geographical Locations among Migrant and Non-Migrant Ghanaians-The RODAM Study.	Cross-sectional	Non probabilistic	Consecutive sampling	Community-based	Multicenter	Prospetively	Ghana	Africa	West Africa	Lower-middle income economies	2012-2015	Adults: 18+ years	Apparently healthy individuals	JIS, 2009
333	Veldsman, 2022_South Africa	The relationship between physical activity, body fatness and metabolic syndrome in urban South African school teachers: The sympathetic activity and ambulatory blood pressure in Africans study.	Cross-sectional	Non probabilistic	Consecutive sampling	Hospital-based	Monocenter	Prospetively	South Africa	Africa	Southern Africa	Upper-middle-income economies	2008-2012	Adults: 18+ years	Apparently healthy individuals	JIS, 2009
335	Woldeyes, 2022_Ethiopia	Prevalence of Clinical Cardiovascular Disease Risk Factors Among HIV Infected Patients on Anti-Retroviral Treatment in a Tertiary Hospital in Ethiopia.	Cross-sectional	Non probabilistic	Consecutive sampling	Hospital-based	Monocenter	Prospetively	Ethiopia	Africa	Eastern Africa	Low-income economies	Jan/2020-Feb/2020	Adults: 18+ years	HIV infected patients	NCEP-ATP III, 2001
336	Woldu, 2022_Ethiopia	Biomarkers and Prevalence of Cardiometabolic Syndrome Among People Living With HIV/AIDS, Addis Ababa, Ethiopia: A Hospital-Based Study.	Cross-sectional	Non probabilistic	Consecutive sampling	Hospital-based	Monocenter	Prospetively	Ethiopia	Africa	Eastern Africa	Low-income economies	Jan/2019-Feb/2021	Adults: 18+ years	HIV infected patients	IDF, 2005
