# Supplementary material for: Prevalence of the metabolic syndrome in African populations: A systematic review and meta-analysis
Source: PLoS One. 2023 Jul 27;18(7):e0289155. doi: 10.1371/journal.pone.0289155 (PMC10374159; doi:10.1371/journal.pone.0289155)
Supplement: S6 Table — (PDF) [file pone.0289155.s008.pdf]

| Unique_ID | Author, Year (Year of publication)_Countries | Title | Was the study's target population a close representation of the national population in relation to MS prevalence or CFR in pregnant? | Was the sampling frame a true or close representation of the target population? | Was some form of random selection used to select the sample, OR was acensus undertaken? | Were data collected directly from the subjects (as opposed to a proxy)? | Was an acceptable inclusion criteria definition used in the study? | Did the author calculate and respect the expected sample size? | Was the MS definition shown to have reliability and validity? | Was the same mode of data collection used for all subjects? | Was the length of the study period > or = 1 year? | Were the numerator(s) and denominator(s) for the MS prevalence/CFR appropriate? | Score | Risk of bias | Population categories | MS definition |
|---|---|---|---|---|---|---|---|---|---|---|---|---|---|---|---|---|
| 1 | Abagre, 2022_Ghana | Determinants of metabolic syndrome among patients attending diabetes clinics in two sub-urban hospitals: Bono Region, Ghana. | No | Yes | No | Yes | Yes | Yes | Yes | Yes | No | Yes | 7 | Low risk of bias | Type 2 diabetes patients | JIS, 2009 |
| 2 | Abd Elaziz, 2015_Egypt | Prevalence of metabolic syndrome and cardiovascular risk factors among voluntary screened middle-aged and elderly Egyptians. | No | Yes | No | Yes | Yes | Yes | Yes | Yes | Unclear | Yes | 7 | Low risk of bias | Apparently healthy individuals | Revised NCEP-ATP III, 2005 |
| 3 | Abda, 2016_Ethiopia | Metabolic syndrome and associated factors among outpatients of Jimma University Teaching Hospital. | No | Yes | No | Yes | Yes | No | Yes | Yes | Unclear | Yes | 6 | Moderate risk of bias | Apparently healthy individuals | NCEP-ATP III, 2001 |
| 4 | Abebe, 2021_Ethiopia | Magnitude of metabolic syndrome in Gondar town, Northwest Ethiopia: A community-based cross-sectional study. | No | Yes | No | Yes | Yes | Yes | Yes | Yes | Yes | Yes | 8 | Low risk of bias | Apparently healthy individuals | IDF, 2005 |
| 5 | Abourazzak, 2014_Morocco | Prevalence of metabolic syndrome in patients with rheumatoid arthritis in Morocco: a cross-sectional study of 179 cases. | No | Yes | No | Yes | Yes | No | Yes | Yes | Yes | Yes | 7 | Low risk of bias | Apparently healthy individuals | IDF, 2005 |
| 6 | Abourazzak, 2014_Morocco | Prevalence of metabolic syndrome in patients with rheumatoid arthritis in Morocco: a cross-sectional study of 179 cases. | No | Yes | No | Yes | Yes | No | Yes | Yes | Yes | Yes | 7 | Low risk of bias | Patients with rheumatoid arthritis | IDF, 2005 |
| 7 | Abourazzak, 2015_Morocco | Does metabolic syndrome or its individual components affect pain and function in knee osteoarthritis women? | No | Yes | No | Yes | Yes | No | Yes | Yes | Unclear | Yes | 6 | Moderate risk of bias | Patients with rheumatoid arthritis | Revised NCEP-ATP III, 2005 |
| 8 | Achila, 2022_Eritrea | Metabolic syndrome, associated factors and optimal waist circumference cut points: findings from a cross-sectional community-based study in the elderly population in Asmara, Eritrea. | No | Yes | No | Yes | Yes | Yes | Yes | Yes | No | Yes | 7 | Low risk of bias | Apparently healthy individuals | JIS, 2009 |
| 9 | Adediran, 2012_Nigeria | Impact of urbanization and gender on frequency of metabolic syndrome among native Abuja settlers in Nigeria. | No | Yes | No | Yes | Yes | No | Yes | Yes | Unclear | Yes | 6 | Moderate risk of bias | Apparently healthy individuals | IDF, 2005 |
| 10 | Adedoyin, 2013_Nigeria | Relationship between socioeconomic status and metabolic syndrome among Nigerian adults. | No | Yes | No | Yes | Yes | No | Yes | Yes | Unclear | Yes | 6 | Moderate risk of bias | Apparently healthy individuals | IDF, 2005 |
| 11 | Adegoke, 2010_Nigeria | Prevalence of metabolic syndrome in a rural community in Nigeria. | No | Yes | No | Yes | Yes | No | Yes | Yes | Unclear | Yes | 6 | Moderate risk of bias | Apparently healthy individuals | NCEP-ATP III, 2001 |
| 12 | Adejumo, 2019_Nigeria | Anthropometric parameter that best predict metabolic syndrome in South west Nigeria. | No | Yes | No | Yes | Yes | Yes | Yes | Yes | Unclear | Yes | 7 | Low risk of bias | Apparently healthy individuals | JIS, 2009 |
| 13 | Adeleye, 2014_Nigeria | Metabolic Syndrome in Patients attending the Staff Clinic of a Nigerian Tertiary Hospital. | No | Yes | No | Yes | Yes | Yes | Yes | Yes | Unclear | Yes | 7 | Low risk of bias | Apparently healthy individuals | IDF, 2005 |
| 14 | Adeoye, 2015_Nigeria | Excess Metabolic Syndrome Risks Among Women Health Workers Compared With Men. | No | Yes | No | Yes | Yes | No | Yes | Yes | Unclear | Yes | 6 | Moderate risk of bias | Apparently healthy individuals | IDF, 2005 |
| 15 | Adeyeye, 2012_Nigeria | Understanding asthma and the metabolic syndrome - a Nigerian report. | No | Yes | No | Yes | Yes | No | Yes | Yes | Unclear | Yes | 6 | Moderate risk of bias | Patients with respiratory tract diseases | JIS, 2009 |
| 16 | Agaba, 2019_Uganda | Prevalence and Associated Factors of Metabolic Syndrome among Patients with Severe Mental Illness Attending a Tertiary Hospital in Southwest Uganda. | No | Yes | No | Yes | Yes | No | Yes | Yes | No | Yes | 6 | Moderate risk of bias | Psychiatric patients | NCEP-ATP III, 2001 |
| 17 | Agu, 2019_Nigeria | Prevalence and associated risk factors of peripheral artery disease in virologically suppressed HIV-infected individuals on antiretroviral therapy in Kwara state, Nigeria: a cross sectional study. | No | Yes | No | Yes | Yes | Yes | Yes | Yes | Unclear | Yes | 7 | Low risk of bias | Apparently healthy individuals | IDF, 2005 |
| 18 | Agu, 2019_Nigeria | Prevalence and associated risk factors of peripheral artery disease in virologically suppressed HIV-infected individuals on antiretroviral therapy in Kwara state, Nigeria: a cross sectional study. | No | Yes | No | Yes | Yes | Yes | Yes | Yes | Unclear | Yes | 7 | Low risk of bias | HIV infected patients | IDF, 2005 |
| 19 | Agyemang-Yeboah, 2019_Ghana | Evaluation of Metabolic Syndrome and Its Associated Risk Factors in Type 2 Diabetes: A Descriptive Cross-Sectional Study at the Komfo Anokye Teaching Hospital, Kumasi, Ghana. | No | Yes | No | Yes | Yes | Yes | Yes | Yes | Unclear | Yes | 7 | Low risk of bias | Type 2 diabetes patients | Revised NCEP-ATP III, 2005 |
| 20 | Ahmed, 2015_Sudan | Metabolic Syndrome among Undergraduate Students Attending Medical Clinics for Obligatory Medical Screening. | No | Yes | No | Yes | Yes | No | Yes | Yes | Unclear | Yes | 6 | Moderate risk of bias | Apparently healthy individuals | NCEP-ATP III, 2001 |
| 21 | Ajayi, 2014_Nigeria | Metabolic syndrome: prevalence and association with electrocardiographic abnormalities in Nigerian hypertensive patients. | No | Yes | No | Yes | Yes | No | Yes | Yes | Unclear | Yes | 6 | Moderate risk of bias | Hypertensive patients | Revised NCEP-ATP III, 2005 |
| 22 | Ajemu, 2021_Ethiopia | Magnitude, components and predictors of metabolic syndrome in Northern Ethiopia: Evidences from regional NCDs STEPS survey, 2016. | No | Yes | No | Yes | Yes | No | Yes | Yes | Unclear | Yes | 6 | Moderate risk of bias | Apparently healthy individuals | NCEP-ATP III, 2001 |
| 23 | Akande, 2007_Nigeria | Serum uric acid level as an independent component of the metabolic syndrome in type 2 diabetic blacks. | No | Yes | No | Yes | Yes | No | Yes | Yes | Unclear | Yes | 6 | Moderate risk of bias | Type 2 diabetes patients | WHO,1998 |
| 24 | Akintunde, 2011_Nigeria | Metabolic syndrome: comparison of occurrence using three definitions in hypertensive patients. | No | Yes | No | Yes | Yes | No | Yes | Yes | Unclear | Yes | 6 | Moderate risk of bias | Hypertensive patients | IDF, 2005 |
| 25 | Akintunde, 2017_Nigeria | Metabolic syndrome and occupation: Any association? Prevalence among auto technicians and school teachers in South West Nigeria. | No | Yes | No | Yes | Yes | No | Yes | Yes | Unclear | Yes | 6 | Moderate risk of bias | Apparently healthy individuals | JIS, 2009 |
| 26 | Akinyemiju, 2022_Nigeria | Metabolic Syndrome and Risk of Breast Cancer by Molecular Subtype: Analysis of the MEND Study. | No | Yes | No | Yes | Yes | No | Yes | Yes | Yes | Yes | 7 | Low risk of bias | Apparently healthy individuals | JIS, 2009 |
| 27 | Akinyemiju, 2022_Nigeria | Metabolic Syndrome and Risk of Breast Cancer by Molecular Subtype: Analysis of the MEND Study. | No | Yes | No | Yes | Yes | No | Yes | Yes | Yes | Yes | 7 | Low risk of bias | Patients with chronic diseases | JIS, 2009 |
| 28 | Akpalu, 2011_Ghana | The metabolic syndrome among patients with cardiovascular disease in Accra, Ghana. | No | Yes | No | Yes | Yes | Yes | Yes | Yes | Unclear | Yes | 7 | Low risk of bias | Apparently healthy individuals | NCEP-ATP III, 2001 |
| 29 | Akpalu, 2011_Ghana | The metabolic syndrome among patients with cardiovascular disease in Accra, Ghana. | No | Yes | No | Yes | Yes | Yes | Yes | Yes | Unclear | Yes | 7 | Low risk of bias | Patients with cardiovascular diseases | NCEP-ATP III, 2001 |
| 30 | Alebiosu, 2004_Nigeria | Metabolic syndrome in subjects with type-2 diabetes mellitus. | No | Yes | No | Yes | Yes | No | Yes | Yes | Yes | Yes | 7 | Low risk of bias | Type 2 diabetes patients | WHO,1998 |
| 31 | Ali, 2021_Egypt | Prevalence and Predictors of Metabolic Syndrome among Patients with Bronchial Asthma: A Cross Sectional Study. | No | Yes | No | Yes | Yes | Yes | Yes | Yes | Unclear | Yes | 7 | Low risk of bias | Patients with respiratory tract diseases | IDF, 2005 |
| 32 | Allal-Elasmi, 2010_Tunisia | The metabolic syndrome: prevalence, main characteristics and association with socio-economic status in adults living in Great Tunis. | No | Yes | No | Yes | Yes | No | Yes | Yes | Unclear | Yes | 6 | Moderate risk of bias | Apparently healthy individuals | Revised NCEP-ATP III, 2005 |
| 33 | Allal-Elasmi, 2012_Tunisia | Prehypertension among adults in Great Tunis region (Tunisia): A population-based study. | No | Yes | No | Yes | Yes | No | Yes | Yes | Unclear | Yes | 6 | Moderate risk of bias | Apparently healthy individuals | NCEP-ATP III, 2001 |
| 34 | Allal-Elasmi, 2012_Tunisia | Prehypertension among adults in Great Tunis region (Tunisia): A population-based study. | No | Yes | No | Yes | Yes | No | Yes | Yes | Unclear | Yes | 6 | Moderate risk of bias | Hypertensive patients | NCEP-ATP III, 2001 |
| 35 | Almobarak, 2020_Sudan | The prevalence of diabetes and metabolic syndrome and associated risk factors in Sudanese individuals with gallstones: a cross sectional survey. | No | Yes | No | Yes | Yes | Yes | Yes | Yes | Unclear | Yes | 7 | Low risk of bias | Patients with dermatological diseases | NCEP-ATP III, 2001 |
| 36 | Alshkri, 2008_Libya | Metabolic Syndrome among Type-2 Diabetic Patients in Benghazi-Libya: A pilot study. | No | Yes | No | Yes | Yes | No | Yes | Yes | Unclear | Yes | 6 | Moderate risk of bias | Type 2 diabetes patients | Revised NCEP-ATP III, 2005 |
| 37 | Aly El-Gabry, 2018_Egypt | Antipsychotic Polypharmacy and Its Relation to Metabolic Syndrome in Patients With Schizophrenia: An Egyptian Study. | No | Yes | No | Yes | Yes | Yes | Yes | Yes | Yes | Yes | 8 | Low risk of bias | Psychiatric patients | IDF, 2005 |
| 38 | Amidu, 2013_Ghana | Association between metabolic syndrome and sexual dysfunction among men with clinically diagnosed diabetes. | No | Yes | No | Yes | Yes | No | Yes | Yes | No | Yes | 6 | Moderate risk of bias | Type 2 diabetes patients | WHO,1998 |
| 39 | Amidu, 2019_Ghana | Comparative Abilities of Fasting Plasma Glucose and Haemoglobin A1c in Predicting Metabolic Syndrome among Apparently Healthy Normoglycemic Ghanaian Adults. | No | Yes | No | Yes | Yes | Yes | Yes | Yes | No | Yes | 7 | Low risk of bias | Apparently healthy individuals | JIS, 2009 |
| 40 | Annani-Akollor, 2019_Ghana | Prevalence of metabolic syndrome and the comparison of fasting plasma glucose and HbA1c as the glycemic criterion for MetS definition in non-diabetic population in Ghana. | No | Yes | No | Yes | Yes | Yes | Yes | Yes | Unclear | Yes | 7 | Low risk of bias | Apparently healthy individuals | JIS, 2009 |
| 41 | Annani-Akollor, 2020_Ghana | Population-derived cut-off for HbA1c could enhance the identification of metabolic syndrome among non-diabetic population. | No | Yes | No | Yes | Yes | Yes | Yes | Yes | No | Yes | 7 | Low risk of bias | Apparently healthy individuals | IDF, 2005 |
| 42 | Aouam, 2021_Tunisia | [Metabolic syndrome among people with HIV in central Tunisia: Prevalence and associated factors]. | No | Yes | No | Yes | Yes | No | Yes | Yes | Unclear | Yes | 6 | Moderate risk of bias | HIV infected patients | IDF, 2005 |
| 43 | Appiah, 2020_Ghana | Prevalence and lifestyle-associated risk factors of metabolic syndrome among commercial motor vehicle drivers in a metropolitan city in Ghana. | No | Yes | No | Yes | Yes | No | Yes | Yes | Unclear | Yes | 6 | Moderate risk of bias | Apparently healthy individuals | Revised NCEP-ATP III, 2005 |
| 44 | Arthur, 2012_Ghana | Prediction of metabolic syndrome among postmenopausal Ghanaian women using obesity and atherogenic markers. | No | Yes | No | Yes | Yes | Yes | Yes | Yes | Unclear | Yes | 7 | Low risk of bias | Postmenopausal women | JIS, 2009 |
| 45 | Arthur, 2013_Ghana | The prevalence of metabolic syndrome and its predominant components among pre-and postmenopausal Ghanaian women. | No | Yes | No | Yes | Yes | Yes | Yes | Yes | Unclear | Yes | 7 | Low risk of bias | Apparently healthy individuals | JIS, 2009 |
| 46 | Arthur, 2013_Ghana | The prevalence of metabolic syndrome and its predominant components among pre-and postmenopausal Ghanaian women. | No | Yes | No | Yes | Yes | Yes | Yes | Yes | Unclear | Yes | 7 | Low risk of bias | Postmenopausal women | JIS, 2009 |
| 47 | Asaye, 2018_Ethiopia | Metabolic syndrome and associated factors among psychiatric patients in Jimma University Specialized Hospital, South West Ethiopia. | No | Yes | No | Yes | Yes | Yes | Yes | Yes | Unclear | Yes | 7 | Low risk of bias | Psychiatric patients | IDF, 2005 |
| 48 | Assaad-Khalil, 2015_Egypt | Optimal waist circumference cutoff points for the determination of abdominal obesity and detection of cardiovascular risk factors among adult Egyptian population. | No | Yes | Yes | Yes | Yes | Yes | Yes | Yes | Yes | Yes | 9 | Low risk of bias | Apparently healthy individuals | IDF, 2005 |
| 49 | Assah, 2011_Cameroon | Urbanization, physical activity, and metabolic health in sub-Saharan Africa. | No | Yes | No | Yes | Yes | No | Yes | Yes | Unclear | Yes | 6 | Moderate risk of bias | Apparently healthy individuals | Revised NCEP-ATP III, 2005 |
| 50 | Ayina Ayina, 2014_Cameroon | Osteoprotegerin is not a determinant of metabolic syndrome in sub-Saharan Africans after age adjustment. | No | Yes | No | Yes | Yes | No | Yes | Yes | Unclear | Yes | 6 | Moderate risk of bias | Apparently healthy individuals | JIS, 2009 |
| 51 | Ayodele, 2012_Nigeria | Prevalence and clinical correlates of metabolic syndrome in Nigerians living with human immunodeficiency virus/acquired immunodeficiency syndrome. | No | Yes | No | Yes | Yes | No | Yes | Yes | Unclear | Yes | 6 | Moderate risk of bias | HIV infected patients | JIS, 2009 |
| 52 | Ayogu, 2019_Nigeria | Components and Risk Factors of Metabolic Syndrome among Rural Nigerian Workers. | No | Yes | No | Yes | Yes | No | Yes | Yes | Unclear | Yes | 6 | Moderate risk of bias | Apparently healthy individuals | IDF, 2005 |
| 53 | Babiker, 2018_Sudan | Risk factors of metabolic syndrome among adult Sudanese sickle cell anemia patients. | No | Yes | No | Yes | Yes | No | Yes | Yes | No | Yes | 6 | Moderate risk of bias | Apparently healthy individuals | NCEP-ATP III, 2001 |
| 54 | Babiker, 2018_Sudan | Risk factors of metabolic syndrome among adult Sudanese sickle cell anemia patients. | No | Yes | No | Yes | Yes | No | Yes | Yes | No | Yes | 6 | Moderate risk of bias | patients with sickle cell disease | NCEP-ATP III, 2001 |
| 55 | Bachir Cherif, 2016_Algeria | [The characteristics of arterial hypertension in postmenopausal women in the area of Blida (Algeria)]. | No | Yes | No | Yes | Yes | No | Yes | Yes | Yes | Yes | 7 | Low risk of bias | Hypertensive patients | NCEP-ATP III, 2001 |
| 56 | Bakry, 2014_Egypt | Androgenetic alopecia, metabolic syndrome, and insulin resistance: Is there any association? A case-control study. | No | Yes | No | Yes | Yes | No | Yes | Yes | Yes | Yes | 7 | Low risk of bias | Apparently healthy individuals | NCEP-ATP III, 2001 |
| 57 | Bakry, 2014_Egypt | Androgenetic alopecia, metabolic syndrome, and insulin resistance: Is there any association? A case-control study. | No | Yes | No | Yes | Yes | No | Yes | Yes | Yes | Yes | 7 | Low risk of bias | Patients with dermatological diseases | NCEP-ATP III, 2001 |

| | | | | | | | | | | | | | | | | |
|---|---|---|---|---|---|---|---|---|---|---|---|---|---|---|---|---|
| 58 | Balti, 2013_Cameroon | Metabolic syndrome and fatal outcomes in the post-stroke event: a 5-year cohort study in Cameroon. | No | Yes | No | Yes | Yes | No | Yes | Yes | Unclear | Yes | 6 | Moderate risk of bias | Patients with cardiovascular diseases | JIS, 2009 |
| 59 | Belarbia, 2015_Tunisia | Metabolic syndrome and chronic kidney disease. | No | Yes | No | Yes | Yes | No | Yes | Yes | Unclear | Yes | 6 | Moderate risk of bias | Patients with chronic kidney diseases | NCEP-ATP III, 2001 |
| 60 | Belfki, 2013_Tunisia | Prevalence and determinants of the metabolic syndrome among Tunisian adults: results of the Transition and Health Impact in North Africa (TAHINA) project. | No | Yes | No | Yes | Yes | Yes | Yes | Yes | No | Yes | 7 | Low risk of bias | Apparently healthy individuals | NCEP-ATP III, 2001 |
| 61 | Bello-Rodriguez, 2013_Ghana | The relationship between metabolic syndrome and target organ damage in Ghanaian with stage-2 hypertension. | No | Yes | No | Yes | Yes | Yes | Yes | Yes | No | Yes | 7 | Low risk of bias | Hypertensive patients | Revised NCEP-ATP III, 2005 |
| 62 | Ben Ali, 2012_Tunisia | Adiponectin and metabolic syndrome in a Tunisian population. | No | Yes | No | Yes | Yes | No | Yes | Yes | Yes | Yes | 7 | Low risk of bias | Apparently healthy individuals | NCEP-ATP III, 2001 |
| 63 | Ben Ali, 2014_Tunisia | Menopause and metabolic syndrome in tunisian women. | No | Yes | Yes | Yes | Yes | No | Yes | Yes | No | Yes | 7 | Low risk of bias | Apparently healthy individuals | NCEP-ATP III, 2001 |
| 64 | Ben Ali, 2014_Tunisia | Menopause and metabolic syndrome in tunisian women. | No | Yes | Yes | Yes | Yes | No | Yes | Yes | No | Yes | 7 | Low risk of bias | Postmenopausal women | NCEP-ATP III, 2001 |
| 65 | Ben Jazia, 2015_Tunisia | [Prevalence of metabolic syndrome in a population of apneic patients: a prospective study with cardiovascular risk estimation]. | No | Yes | No | Yes | Yes | No | Yes | Yes | Unclear | Yes | 6 | Moderate risk of bias | Patients with respiratory tract diseases | IDF, 2005 |
| 66 | Benmohammed, 2015_Algeria | Anthropometric markers for detection of the metabolic syndrome in adolescents. | No | Yes | Yes | Yes | Yes | No | Yes | Yes | No | Yes | 7 | Low risk of bias | Apparently healthy individuals | IDF, 2005 |
| 67 | Bennouar, 2021_Algeria | Association and interaction between vitamin D level and metabolic syndrome for non-alcoholic fatty liver disease. | No | Yes | No | Yes | Yes | Yes | Yes | Yes | Yes | Yes | 8 | Low risk of bias | Apparently healthy individuals | NCEP-ATP III, 2001 |
| 68 | Bennouar, 2021_Algeria | Association and interaction between vitamin D level and metabolic syndrome for non-alcoholic fatty liver disease. | No | Yes | No | Yes | Yes | Yes | Yes | Yes | Yes | Yes | 8 | Low risk of bias | Patients with nonalcoholic fatty liver disease | NCEP-ATP III, 2001 |
| 69 | Ben-Yacov, 2020_Uganda | Prevalence and sex-specific patterns of metabolic syndrome in rural Uganda. | No | Yes | Yes | Yes | Yes | No | Yes | Yes | Unclear | Yes | 7 | Low risk of bias | Apparently healthy individuals | NCEP-ATP III, 2001 |
| 70 | Berhane, 2012_Ethiopia | Prevalence of lipodystrophy and metabolic syndrome among HIV positive individuals on Highly Active Anti-Retroviral treatment in Jimma, South West Ethiopia. | No | Yes | Yes | Yes | Yes | Yes | Yes | Yes | No | Yes | 8 | Low risk of bias | HIV infected patients | Revised NCEP-ATP III, 2005 |
| 71 | Biadgo, 2018_Ethiopia | The Prevalence of Metabolic Syndrome and Its Components among Type 2 Diabetes Mellitus Patients at a Tertiary Hospital, Northwest Ethiopia. | No | Yes | Yes | Yes | Yes | Yes | Yes | Yes | No | Yes | 8 | Low risk of bias | Type 2 diabetes patients | Revised NCEP-ATP III, 2005 |
| 72 | Birarra, 2018_Ethiopia | Metabolic syndrome among type 2 diabetic patients in Ethiopia: a cross-sectional study. | No | Yes | No | Yes | Yes | Yes | Yes | Yes | No | Yes | 7 | Low risk of bias | Type 2 diabetes patients | NCEP-ATP III, 2001 |
| 73 | Bizuayehu Wube, 2019_Ethiopia | A Comparative Prevalence Of Metabolic Syndrome Among Type 2 Diabetes Mellitus Patients In Hawassa University Comprehensive Specialized Hospital Using Four Different Diagnostic Criteria. | No | Yes | Yes | Yes | Yes | Yes | Yes | Yes | No | Yes | 8 | Low risk of bias | Type 2 diabetes patients | NCEP-ATP III, 2001 |
| 74 | Bjerregaard-Andersen, 2013_Guin | Risk of metabolic syndrome and diabetes among young twins and singletons in Guinea-Bissau. | No | Yes | No | Yes | Yes | No | Yes | Yes | Yes | Yes | 7 | Low risk of bias | Apparently healthy individuals | IDF, 2005 |
| 75 | Bojang, 2021_Gambia | Prevalence of Metabolic Syndrome and its Components in Kanifing Municipality, The Gambia. | No | Yes | No | Yes | Yes | No | Yes | Yes | No | Yes | 6 | Moderate risk of bias | Apparently healthy individuals | JIS, 2009 |
| 76 | Borgo, 2018_Angola | Lipid disorders among Black Africans non-users of lipid-lowering medication. | No | Yes | No | Yes | Yes | No | Yes | Yes | Unclear | Yes | 6 | Moderate risk of bias | Apparently healthy individuals | NCEP-ATP III, 2001 |
| 77 | Boronat, 2018_Ethiopia | Prevalence of the metabolic syndrome in the island of Gran Canaria: comparison of three major diagnostic proposals. | No | Yes | No | Yes | Yes | Yes | Yes | Yes | Unclear | Yes | 7 | Low risk of bias | HIV infected patients | JIS, 2009 |
| 78 | Bosho, 2018_Ethiopia | Prevalence and predictors of metabolic syndrome among people living with human immunodeficiency virus (PLWHIV). | No | Yes | No | Yes | Yes | Yes | Yes | Yes | Unclear | Yes | 7 | Low risk of bias | HIV infected patients | JIS, 2009 |
| 79 | Bouguerra, 2006_Tunisia | Prevalence of metabolic abnormalities in the Tunisian adults: a population based study. | No | Yes | No | Yes | Yes | No | Yes | Yes | Unclear | Yes | 6 | Moderate risk of bias | Apparently healthy individuals | NCEP-ATP III, 2001 |
| 80 | Boumaiza, 2010_Tunisia | Apolipoprotein B and non-high-density lipoprotein cholesterol are better risk markers for coronary artery disease than low-density lipoprotein cholesterol in hypertriglyceridemic metabolic syndrome patients. | No | Yes | No | Yes | Yes | No | Yes | Yes | Unclear | Yes | 6 | Moderate risk of bias | Patients with coronary artery disease | IDF, 2005 |
| 81 | Bovet, 2006_Seychelles | Prevalence of cardiovascular risk factors in a middle-income country and estimated cost of a treatment strategy. | No | Yes | No | Yes | Yes | No | Yes | Yes | Unclear | Yes | 6 | Moderate risk of bias | Apparently healthy individuals | NCEP-ATP III, 2001 |
| 82 | Bovet, 2009_Seychelles | Divergent fifteen-year trends in traditional and cardiometabolic risk factors of cardiovascular diseases in the Seychelles. | No | Yes | No | Yes | Yes | No | Yes | Yes | Unclear | Yes | 6 | Moderate risk of bias | Apparently healthy individuals | Revised NCEP-ATP III, 2005 |
| 83 | Bune, 2019_Ethiopia | The global magnitude of metabolic syndrome among antiretroviral therapy (ART) exposed and ART-naive adult HIV-infected patients in gedio-zone, southern Ethiopia: Comparative cross-sectional study, using the Adult Treatment Panel III criteria. | No | Yes | Yes | Yes | Yes | Yes | Yes | Yes | Yes | No | 8 | Low risk of bias | HIV infected patients | Revised NCEP-ATP III, 2005 |
| 84 | Challa, 2021_Ethiopia | Prevalence of metabolic syndrome among patients with schizophrenia in Ethiopia. | No | Yes | No | Yes | Yes | No | Yes | Yes | Yes | Yes | 7 | Low risk of bias | Psychiatric patients | Revised NCEP-ATP III, 2005 |
| 85 | Chanda, 2010_Zambia | Predictive value of Metabolic Syndrome components in detecting the syndrome in patients with type 2 Diabetes Mellitus. | No | Yes | No | Yes | Yes | No | Yes | Yes | Unclear | Yes | 6 | Moderate risk of bias | Type 2 diabetes patients | NCEP-ATP III, 2001 |
| 86 | Chivese, 2019_South Africa | High prevalence of cardiovascular risk factors and insulin resistance 6 years after hyperglycemia first detected in pregnancy in Cape Town, South Africa. | No | Yes | No | Yes | Yes | No | Yes | Yes | Yes | Yes | 7 | Low risk of bias | Type 2 diabetes patients | NCEP-ATP III, 2001 |
| 87 | Christian, 2021_Ghana | Metabolic syndrome among individuals living with hypertension in Accra, Ghana. | No | Yes | Yes | Yes | Yes | No | Yes | Yes | Yes | Yes | 8 | Low risk of bias | Hypertensive patients | IDF, 2005 |
| 88 | Cissé, 2021_Burkina Faso | Using the first nationwide survey on non-communicable disease risk factors and different definitions to evaluate the prevalence of metabolic syndrome in Burkina Faso. | Yes | Yes | Yes | Yes | Yes | Yes | Yes | Yes | No | Yes | 9 | Low risk of bias | Apparently healthy individuals | JIS, 2009 |
| 89 | Cole, 2017_Ghana | Sleep duration is associated with increased risk for cardiovascular outcomes: a pilot study in a sample of community dwelling adults in Ghana. | No | Yes | Yes | Yes | Yes | Yes | Yes | Yes | Unclear | Yes | 8 | Low risk of bias | Apparently healthy individuals | JIS, 2009 |
| 90 | Dada, 2016_Nigeria | Metabolic Syndrome and Framingham Risk Score: Observation from Screening of Low-Income Semi-Urban African Women. | No | Yes | No | Yes | Yes | No | Yes | Yes | Unclear | Yes | 6 | Moderate risk of bias | Apparently healthy individuals | IDF, 2005 |
| 91 | Distiller, 2010_South Africa | The effect of features of the metabolic syndrome on atherosclerotic risk in relatively long-surviving patients with type 1 diabetes. | No | Yes | No | Yes | Yes | No | Yes | Yes | Unclear | Yes | 6 | Moderate risk of bias | Patients with type 1 diabetes | IDF, 2005 |
| 92 | Dos Prazeres Tavares, 2016_Ang | Prevalence of metabolic syndrome in non-diabetic, pregnant Angolan women according to four diagnostic criteria and its effects on adverse perinatal outcomes. | No | Yes | No | Yes | Yes | No | Yes | Yes | No | Yes | 6 | Moderate risk of bias | Pregnant women | JIS, 2009 |
| 93 | Doualla-Bija, 2018_Cameroon | Prevalence and characteristics of metabolic syndrome in gout patients in a hospital setting in sub-Saharan Africa. | No | Yes | No | Yes | Yes | Yes | Yes | Yes | No | Yes | 7 | Low risk of bias | Patients with Chronic musculoskeletal diseases | IDF, 2005 |
| 94 | Duki, 2016_South Africa | Relationship of Body Anthropometry with Cardiovascular Risk Factors in a Random Community Sample: The Phoenix Lifestyle Project. | No | Yes | Yes | Yes | Yes | Yes | Yes | Yes | Unclear | Yes | 8 | Low risk of bias | Apparently healthy individuals | WHO,1998 |
| 95 | El Ati, 2020_Tunisia | Metabolic syndrome, malnutrition, and its associations with cardiovascular and all-cause mortality in hemodialysis patients: Follow-up for three years. | No | Yes | No | Yes | Yes | No | Yes | Yes | Yes | Yes | 7 | Low risk of bias | Patients with chronic kidney diseases | NCEP-ATP III, 2001 |
| 96 | El Brini, 2014_Morocco | Prevalence of metabolic syndrome and its components based on a harmonious definition among adults in Morocco. | No | Yes | No | Yes | Yes | Yes | Yes | Yes | Yes | Yes | 8 | Low risk of bias | Apparently healthy individuals | JIS, 2009 |
| 97 | El Maghraoui, 2014_Morocco | Osteoporosis, vertebral fractures and metabolic syndrome in postmenopausal women. | No | Yes | No | Yes | Yes | Yes | Yes | Yes | No | Yes | 7 | Low risk of bias | Postmenopausal women | NCEP-ATP III, 2001 |
| 98 | El Ray, 2013_Egypt | Insulin resistance: a major factor associated with significant liver fibrosis in Egyptian patients with genotype 4 chronic hepatitis C. | No | Yes | No | Yes | Yes | No | Yes | Yes | Unclear | Yes | 6 | Moderate risk of bias | Patients with chronic diseases | Revised NCEP-ATP III, 2005 |
| 99 | El Sayed, 2016_Egypt | Association of metabolic syndrome with female pattern hair loss in women: A case-control study. | No | Yes | No | Yes | Yes | No | Yes | Yes | Unclear | Yes | 6 | Moderate risk of bias | Patients with dermatological diseases | NCEP-ATP III, 2001 |
| 100 | El-Karaksy, 2015_Egypt | The value of different insulin resistance indices in assessment of non-alcoholic fatty liver disease in overweight/obese children. | No | Yes | No | Yes | Yes | No | Yes | Yes | Yes | Yes | 7 | Low risk of bias | Patients with obesity | NCEP-ATP III, 2001 |
| 101 | Elkehili, 2012_Libya | Prevalence of the metabolic syndrome in renal transplant recipients. | No | Yes | No | Yes | Yes | Unclear | Yes | Yes | Unclear | Yes | 6 | Moderate risk of bias | Patients with chronic kidney diseases | NCEP-ATP III, 2001 |
| 102 | El-Koofy, 2012_Egypt | The association of metabolic syndrome, insulin resistance and non-alcoholic fatty liver disease in overweight/obese children. | No | Yes | No | Yes | Yes | No | Yes | Yes | Unclear | Yes | 6 | Moderate risk of bias | Patients with obesity | NCEP-ATP III, 2001 |
| 103 | Elsehely, 2017_Egypt | A Cutoff for Age at Menarche Predicting Metabolic Syndrome in Egyptian Overweight/Obese Premenopausal Women. | No | Yes | No | Yes | Yes | No | Yes | Yes | Unclear | Yes | 6 | Moderate risk of bias | Patients with obesity | NCEP-ATP III, 2001 |
| 104 | Emma-Okon, 2014_Nigeria | The Metabolic Syndrome in an Elite African Community. | No | Yes | No | Yes | Yes | No | Yes | Yes | Unclear | Yes | 6 | Moderate risk of bias | Apparently healthy individuals | IDF, 2005 |
| 105 | Ephraim, 2020_Ghana | Predicting type 2 diabetes mellitus among fishermen in Cape Coast: a comparison between the FINDRISC score and the metabolic syndrome. | No | Yes | No | Yes | Yes | Yes | Yes | Yes | Unclear | Yes | 7 | Low risk of bias | Apparently healthy individuals | IDF, 2005 |
| 106 | Erasmus, 2012_South Africa | High prevalence of diabetes mellitus and metabolic syndrome in a South African coloured population: baseline data of a study in Bellville, Cape Town. | No | Yes | Yes | Yes | Yes | No | Yes | Yes | Yes | Yes | 8 | Low risk of bias | Apparently healthy individuals | JIS, 2009 |
| 107 | Ezeala, 2019_Nigeria | Prevalence and Risk Factors for Metabolic Syndrome among Hypertensive Patients Attending the General Outpatient Clinic of a Tertiary Hospital in North Central Nigeria. | No | Yes | No | Yes | Yes | No | Yes | Yes | No | Yes | 6 | Moderate risk of bias | Hypertensive patients | NCEP-ATP III, 2001 |
| 108 | Ezzaher, 2011_Tunisia | Metabolic syndrome in Tunisian bipolar I patients. | No | Yes | No | Yes | Yes | No | Yes | Yes | Unclear | Yes | 6 | Moderate risk of bias | Psychiatric patients | NCEP-ATP III, 2001 |
| 109 | Faasen, 2014_South Africa | Undiagnosed metabolic syndrome and other adverse effects among clozapine users of Xhosa descent. | No | Yes | No | Yes | Yes | No | Yes | Yes | Unclear | Yes | 6 | Moderate risk of bias | Psychiatric patients | IDF, 2005 |
| 110 | Fahmi, 2020_Egypt | Metabolic syndrome components and disease disability in egyptian multiple sclerosis patients. | No | Yes | No | Yes | Yes | No | Yes | Yes | No | Yes | 6 | Moderate risk of bias | Patients with Chronic musculoskeletal diseases | NCEP-ATP III, 2001 |
| 111 | Fanta, 2021_Ethiopia | Prevalence and Impact of Metabolic Syndrome on Short-Term Prognosis in Patients with Acute Coronary Syndrome: Prospective Cohort Study. | No | Yes | No | Yes | Yes | No | Yes | Yes | No | Yes | 6 | Moderate risk of bias | Patients with cardiovascular diseases | JIS, 2009 |
| 112 | Farhane, 2021_Morocco | COVID-19 pandemic: Effects of national lockdown on the state of health of patients with type 2 diabetes mellitus in a Moroccan population. | No | Yes | No | Yes | Yes | No | Yes | Yes | No | Yes | 6 | Moderate risk of bias | Type 2 diabetes patients | IDF, 2005 |
| 113 | Fentie, 2021_Ethiopia | Metabolic syndrome and associated factors among severely ill psychiatric and non-psychiatric patients: a comparative cross-sectional study in Eastern Ethiopia. | No | Yes | No | Yes | Yes | Yes | Yes | Yes | No | Yes | 7 | Low risk of bias | Apparently healthy individuals | Revised NCEP-ATP III, 2005 |
| 114 | Fentie, 2021_Ethiopia | Metabolic syndrome and associated factors among severely ill psychiatric and non-psychiatric patients: a comparative cross-sectional study in Eastern Ethiopia. | No | Yes | No | Yes | Yes | Yes | Yes | Yes | No | Yes | 7 | Low risk of bias | Psychiatric patients | Revised NCEP-ATP III, 2005 |
| 115 | Fezeu, 2007_Cameroon | Metabolic syndrome in a sub-Saharan African setting: central obesity may be the key determinant. | No | Yes | No | Yes | Yes | No | Yes | Yes | Unclear | Yes | 6 | Moderate risk of bias | Apparently healthy individuals | WHO,1998 |
| 116 | Fouad, 2016_Egypt | Prevalence of obesity and risk of chronic kidney disease among young adults in Egypt. | No | Yes | No | Yes | Yes | No | Yes | Yes | Yes | Yes | 7 | Low risk of bias | Apparently healthy individuals | Revised NCEP-ATP III, 2005 |
| 117 | Fourie, 2010_South Africa | Lipid abnormalities in a never-treated HIV-1 subtype C-infected African population. | No | Yes | Yes | Yes | Yes | No | Yes | Yes | No | Yes | 7 | Low risk of bias | Apparently healthy individuals | IDF, 2005 |

| | | | | | | | | | | | | | | | |
|---|---|---|---|---|---|---|---|---|---|---|---|---|---|---|---|
| 118 | Fourie, 2010_South Africa | Lipid abnormalities in a never-treated HIV-1 subtype C-infected African population. | No | Yes | Yes | Yes | Yes | No | Yes | Yes | No | Yes | 7 | Low risk of bias | HIV infected patients | IDF, 2005 |
| 119 | Frigati, 2019_South Africa | Insulin Resistance in South African Youth Living with Perinatally Acquired HIV Receiving Antiretroviral Therapy. | No | Yes | No | Yes | Yes | No | Yes | Yes | Yes | Yes | 7 | Low risk of bias | Apparently healthy individuals | IDF, 2005 |
| 120 | Frigati, 2019_South Africa | Insulin Resistance in South African Youth Living with Perinatally Acquired HIV Receiving Antiretroviral Therapy. | No | Yes | No | Yes | Yes | No | Yes | Yes | Yes | Yes | 7 | Low risk of bias | HIV infected patients | IDF, 2005 |
| 121 | Gannar, 2015_Tunisia | Social class and metabolic syndrome in populations from Tunisia and Spain. | No | Yes | Yes | Yes | Yes | No | Yes | Yes | Yes | Yes | 8 | Low risk of bias | Apparently healthy individuals | JIS, 2009 |
| 122 | Garrido, 2009_Botswana | Metabolic syndrome and obesity among workers at Kanye Seventh-Day Adventist Hospital, Botswana. | No | Yes | No | Yes | Yes | No | Yes | Yes | No | Yes | 6 | Moderate risk of bias | Apparently healthy individuals | NCEP-ATP III, 2001 |
| 123 | Gebreegziabiher, 2021_Ethiopia | Magnitude and Associated Factors of Metabolic Syndrome Among Adult Urban Dwellers of Northern Ethiopia. | No | Yes | Yes | Yes | Yes | Yes | Yes | Yes | No | Yes | 8 | Low risk of bias | Apparently healthy individuals | IDF, 2005 |
| 124 | Gebremariam, 2018_Ethiopia | Non-communicable disease risk factor profile among public employees in a regional city in northern Ethiopia. | No | Yes | No | Yes | Yes | No | Yes | Yes | No | Yes | 6 | Moderate risk of bias | Apparently healthy individuals | JIS, 2009 |
| 125 | Gebremeskel, 2019_Ethiopia | Magnitude of metabolic syndrome and its associated factors among patients with type 2 diabetes mellitus in Ayder Comprehensive Specialized Hospital, Tigray, Ethiopia: a cross sectional study. | No | Yes | No | Yes | Yes | Yes | Yes | Yes | No | Yes | 7 | Low risk of bias | Type 2 diabetes patients | IDF, 2005 |
| 126 | Gebreyes, 2018_Ethiopia | Prevalence of high bloodpressure, hyperglycemia, dyslipidemia, metabolic syndrome and their determinants in Ethiopia: Evidences from the National NCDs STEPS Survey, 2015. | Yes | Yes | Yes | Yes | Yes | Yes | Yes | Yes | No | Yes | 9 | Low risk of bias | Apparently healthy individuals | IDF, 2005 |
| 127 | Gebreyesus, 2022_Ethiopia | High atherogenic risk concomitant with elevated HbA1c among persons with type 2 diabetes mellitus in North Ethiopia. | No | Yes | No | Yes | Yes | No | Yes | Yes | No | Yes | 6 | Moderate risk of bias | Type 2 diabetes patients | NCEP-ATP III, 2001 |
| 128 | Gebrie, 2020_Ethiopia | The burden of metabolic syndrome in patients living with HIV/AIDS receiving care at referral hospitals of Northwest Ethiopia: A hospital-based cross-sectional study, 2019. | No | Yes | Yes | Yes | Yes | Yes | Yes | Yes | No | Yes | 8 | Low risk of bias | HIV infected patients | IDF, 2005 |
| 129 | George, 2013_South Africa | The association of 25 hydroxyvitamin D and parathyroid hormone with metabolic syndrome in two ethnic groups in South Africa. | No | Yes | No | Yes | Yes | No | Yes | Yes | Unclear | Yes | 6 | Moderate risk of bias | Apparently healthy individuals | JIS, 2009 |
| 130 | Geto, 2021_Ethiopia | Cardiometabolic syndrome and associated factors among Ethiopian public servants, Addis Ababa, Ethiopia. | No | Yes | No | Yes | Yes | No | Yes | Yes | No | Yes | 6 | Moderate risk of bias | Apparently healthy individuals | IDF, 2005 |
| 131 | Ghazali, 2010_Nigeria | Waist circumference, waist to hip ratio, and body mass index in the diagnosis of metabolic syndrome in Nigerian subjects. | No | Yes | No | Yes | Yes | Yes | Yes | Yes | Unclear | Yes | 7 | Low risk of bias | Apparently healthy individuals | NCEP-ATP III, 2001 |
| 132 | Gombet, 2012_Republic of the Co | Aging, female sex, migration, elevated HDL-C, and inflammation are associated with prevalence of metabolic syndrome among African bank employees. | No | Yes | No | Yes | Yes | No | Yes | Yes | Unclear | Yes | 6 | Moderate risk of bias | Apparently healthy individuals | IDF, 2005 |
| 133 | Goolam Mahyoodeen, 2019_South | High burden of the metabolic syndrome and its component disorders in South Africans with psoriasis. | No | Yes | No | Yes | Yes | No | Yes | Yes | Unclear | Yes | 6 | Moderate risk of bias | Apparently healthy individuals | JIS, 2009 |
| 134 | Goolam Mahyoodeen, 2019_South | High burden of the metabolic syndrome and its component disorders in South Africans with psoriasis. | No | Yes | No | Yes | Yes | No | Yes | Yes | Unclear | Yes | 6 | Moderate risk of bias | Patients with dermatological diseases | JIS, 2009 |
| 135 | Govender, 2020_South Africa | A multicentre cross-sectional descriptive study evaluating the cardiovascular risk profile of preoperatively identified patients with hypertension. | No | Yes | No | Yes | Yes | No | Yes | Yes | Unclear | Yes | 6 | Moderate risk of bias | Hypertensive patients | Revised NCEP-ATP III, 2005 |
| 136 | Guira, 2016_Burkina Faso | Features of Metabolic Syndrome and Its Associated Factors during Highly Active Antiretroviral Therapy in Ouagadougou (Burkina Faso). | No | Yes | No | Yes | Yes | Yes | Yes | Yes | Unclear | Yes | 7 | Low risk of bias | HIV infected patients | IDF, 2005 |
| 137 | Gyakobo, 2012_Ghana | Prevalence of the metabolic syndrome in a rural population in Ghana. | No | Yes | No | Yes | Yes | Yes | Yes | Yes | Unclear | Yes | 7 | Low risk of bias | Apparently healthy individuals | IDF, 2005 |
| 138 | Hamed, 2022_Egypt | Androgenetic Alopecia and Metabolic Syndrome: Is Alarin a Missing Link? | No | Yes | No | Yes | Yes | No | Yes | Yes | No | Yes | 6 | Moderate risk of bias | Apparently healthy individuals | IDF, 2005 |
| 139 | Hamed, 2022_Egypt | Androgenetic Alopecia and Metabolic Syndrome: Is Alarin a Missing Link? | No | Yes | No | Yes | Yes | No | Yes | Yes | No | Yes | 6 | Moderate risk of bias | Patients with dermatological diseases | IDF, 2005 |
| 140 | Hamlaoui, 2018_Algeria | Relationship of metabolic syndrome and its components with thyroid dysfunction in Algerian patients. | No | Yes | No | Yes | Yes | No | Yes | Yes | Yes | Yes | 7 | Low risk of bias | Patients with thyroid disease | Revised NCEP-ATP III, 2005 |
| 141 | Hammam, 2018_Egypt | Metabolic syndrome in systemic lupus erythematosus patients : Relationship to disease activity and neuropsychiatric lupus. | No | Yes | No | Yes | Yes | No | Yes | Yes | Unclear | Yes | 6 | Moderate risk of bias | Patients with dermatological diseases | NCEP-ATP III, 2001 |
| 142 | Hamooya , 2021_Zambia | Metabolic syndrome in Zambian adults with human immunodeficiency virus on antiretroviral therapy: Prevalence and associated factors. | No | Yes | Yes | Yes | Yes | Yes | Yes | Yes | Yes | Yes | 9 | Low risk of bias | HIV infected patients | JIS, 2009 |
| 143 | Hanley, 2021_South Africa | Obesity in young South African women living with HIV: A cross-sectional analysis of risk factors for cardiovascular disease. | No | Yes | No | Yes | Yes | No | Yes | Yes | No | Yes | 6 | Moderate risk of bias | HIV infected patients | JIS, 2009 |
| 144 | Hannoun, 2021_Morocco | [Study of the metabolic syndrome and physical activity in a population from Marrakesh, in Morocco]. | No | Yes | No | Yes | Yes | No | Yes | Yes | No | Yes | 6 | Moderate risk of bias | Apparently healthy individuals | NCEP-ATP III, 2001 |
| 145 | Harrabi , 2009_Tunisia | Prevalence of the metabolic syndrome among urban schoolchildren in Sousse, Tunisia. | No | Yes | No | Yes | Yes | No | Yes | Yes | Unclear | Yes | 6 | Moderate risk of bias | Apparently healthy individuals | NCEP-ATP III, 2001 |
| 146 | Harraqui, 2022_Morocco | Frequency of Metabolic Syndrome and Study of Anthropometric, Clinical and Biological Characteristics in Peri- and Postmenopausal Women in the City of Ksar El Kebir (Northern Morocco). | No | Yes | No | Yes | Yes | No | Yes | Yes | Yes | Yes | 7 | Low risk of bias | Postmenopausal women | NCEP-ATP III, 2001 |
| 147 | Harzallah, 2006_Tunisia | The metabolic syndrome in an Arab population: a first look at the new International Diabetes Federation criteria. | No | Yes | Yes | Yes | Yes | No | Yes | Yes | Yes | Yes | 8 | Low risk of bias | Apparently healthy individuals | IDF, 2005 |
| 148 | Hasanain, 2017_Egypt | Erectile dysfunction in patients with nonalcoholic fatty liver disease. | No | Yes | No | Yes | Yes | No | Yes | Yes | Yes | Yes | 7 | Low risk of bias | Patients with nonalcoholic fatty liver disease | NCEP-ATP III, 2001 |
| 149 | Hasni , 2019_Tunisia | Biochemical Data and Metabolic Profiles of Male Exclusive Narghile Smokers (ENSs) Compared With Apparently Healthy Nonsmokers (AHNSs). | No | Yes | No | Yes | Yes | Yes | Yes | Yes | No | Yes | 7 | Low risk of bias | Apparently healthy individuals | IDF, 2005 |
| 150 | Hasni , 2019_Tunisia | Biochemical Data and Metabolic Profiles of Male Exclusive Narghile Smokers (ENSs) Compared With Apparently Healthy Nonsmokers (AHNSs). | No | Yes | No | Yes | Yes | Yes | Yes | Yes | No | Yes | 7 | Low risk of bias | Exclusive narghile smokers | IDF, 2005 |
| 151 | Hassan, 2015_Egypt | Is Neck Circumference an Indicator for Metabolic Complication of Childhood Obesity? | No | Yes | No | Yes | Yes | No | Yes | Yes | No | Yes | 6 | Moderate risk of bias | Apparently healthy individuals | NCEP-ATP III, 2001 |
| 152 | Hassan, 2015_Egypt | Is Neck Circumference an Indicator for Metabolic Complication of Childhood Obesity? | No | Yes | No | Yes | Yes | No | Yes | Yes | No | Yes | 6 | Moderate risk of bias | Patients with obesity | NCEP-ATP III, 2001 |
| 153 | Hassan, 2019_Egypt | Metabolic syndrome in a sample of Egyptian adolescent girls and its association with apolipoprotein E. | No | Yes | No | Yes | Yes | No | Yes | Yes | Yes | Yes | 7 | Low risk of bias | Apparently healthy individuals | IDF, 2005 |
| 154 | Hassan, 2019_Egypt | Metabolic syndrome in a sample of Egyptian adolescent girls and its association with apolipoprotein E. | No | Yes | No | Yes | Yes | No | Yes | Yes | Yes | Yes | 7 | Low risk of bias | Patients with obesity | IDF, 2005 |
| 155 | Hirigo , 2016_Ethiopia | Influences of gender in metabolic syndrome and its components among people living with HIV virus using antiretroviral treatment in Hawassa, southern Ethiopia. | No | Yes | No | Yes | Yes | Yes | Yes | Yes | Unclear | Yes | 7 | Low risk of bias | HIV infected patients | IDF, 2005 |
| 156 | Hoebel , 2012_South Africa | Determining cut-off values for neck circumference as a measure of the metabolic syndrome amongst a South African cohort: the SABPA study. | No | Yes | No | Yes | Yes | No | Yes | Yes | Yes | Yes | 7 | Low risk of bias | Apparently healthy individuals | JIS, 2009 |
| 157 | Hoebel, 2014_South Africa | Optimizing waist circumference cut-points for the metabolic syndrome in a South African cohort at 3-year follow-up: the SABPA prospective cohort. | No | Yes | No | Yes | Yes | No | Yes | Yes | No | No | 5 | Moderate risk of bias | Apparently healthy individuals | JIS, 2009 |
| 158 | Houti , 2016_Algeria | Prevalence of Metabolic Syndrome and its Related Risk Factors in the City of Oran, Algeria: the ISOR Study. | No | Yes | Yes | Yes | Yes | No | Yes | Yes | Yes | Yes | 8 | Low risk of bias | Apparently healthy individuals | NCEP-ATP III, 2001 |
| 159 | Isezuo, 2005_Nigeria | Is high density lipoprotein cholesterol useful in diagnosis of metabolic syndrome in native Africans with type 2 diabetes? | No | Yes | No | Yes | Yes | No | Yes | Yes | Unclear | Yes | 6 | Moderate risk of bias | Type 2 diabetes patients | WHO,1998 |
| 160 | Ishaku, 2021_Nigeria | Metabolic syndrome following hypertensive disorders in pregnancy in a low-resource setting: A cohort study. | No | Yes | No | Yes | Yes | No | Yes | Yes | No | Yes | 6 | Moderate risk of bias | Apparently healthy individuals | IDF, 2005 |
| 161 | Ishaku, 2021_Nigeria | Metabolic syndrome following hypertensive disorders in pregnancy in a low-resource setting: A cohort study. | No | Yes | No | Yes | Yes | No | Yes | Yes | No | Yes | 6 | Moderate risk of bias | Hypertensive patients | IDF, 2005 |
| 162 | Jaballah, 2021_Tunisia | The Relationship Between Menopause and Metabolic Syndrome: Experimental and Bioinformatics Analysis. | No | Yes | No | Yes | Yes | No | Yes | Yes | No | Yes | 6 | Moderate risk of bias | Apparently healthy individuals | NCEP-ATP III, 2001 |
| 163 | Jaballah, 2021_Tunisia | The Relationship Between Menopause and Metabolic Syndrome: Experimental and Bioinformatics Analysis. | No | Yes | No | Yes | Yes | No | Yes | Yes | No | Yes | 6 | Moderate risk of bias | Postmenopausal women | NCEP-ATP III, 2001 |
| 164 | Jamoussi , 2012_Tunisia | Metabolic syndrome in Tunisian obese children and adolescents. | No | Yes | No | Yes | Yes | No | Yes | Yes | No | Yes | 6 | Moderate risk of bias | Patients with obesity | IDF, 2005 |
| 165 | Jean-Luc Gradidge, 2016_South A | Metabolic and Body Composition Risk Factors Associated with Metabolic Syndrome in a Cohort of Women with a High Prevalence of Cardiometabolic Disease. | No | Yes | No | Yes | Yes | No | Yes | Yes | Unclear | Yes | 6 | Moderate risk of bias | Apparently healthy individuals | JIS, 2009 |
| 166 | Jennings, 2009_South Africa | The atypical presentation of the metabolic syndrome components in black African women: the relationship with insulin resistance and the influence of regional adipose tissue distribution. | No | Yes | No | Yes | Yes | No | Yes | Yes | Unclear | Yes | 6 | Moderate risk of bias | Apparently healthy individuals | IDF, 2005 |
| 167 | Jennings, 2009_South Africa | The atypical presentation of the metabolic syndrome components in black African women: the relationship with insulin resistance and the influence of regional adipose tissue distribution. | No | Yes | No | Yes | Yes | No | Yes | Yes | Unclear | Yes | 6 | Moderate risk of bias | Patients with obesity | IDF, 2005 |
| 168 | Jmal, 2019_Tunisia | Prevalence of metabolic syndrome in Tunisian overweight and obese children. | No | Yes | No | Yes | Yes | No | Yes | Yes | Unclear | Yes | 6 | Moderate risk of bias | Apparently healthy individuals | IDF, 2005 |
| 169 | Jmal, 2019_Tunisia | Prevalence of metabolic syndrome in Tunisian overweight and obese children. | No | Yes | No | Yes | Yes | No | Yes | Yes | Unclear | Yes | 6 | Moderate risk of bias | Patients with obesity | IDF, 2005 |
| 170 | Kacem, 2019_Tunisia | Evaluation of the risk of metabolic syndrome among shift workers in Tunisia. | No | Yes | No | Yes | Yes | No | Yes | Yes | Unclear | Yes | 6 | Moderate risk of bias | Apparently healthy individuals | IDF, 2005 |
| 171 | Kaduka, 2012_Kenya | Prevalence of metabolic syndrome among an urban population in Kenya. | No | Yes | Yes | Yes | Yes | No | Yes | Yes | No | Yes | 7 | Low risk of bias | Apparently healthy individuals | JIS, 2009 |
| 172 | Kalk , 2008_South Africa | The metabolic syndrome, insulin resistance, and its surrogates in African and white subjects with type 2 diabetes in South Africa. | No | Yes | No | Yes | Yes | No | Yes | Yes | Yes | Yes | 7 | Low risk of bias | Type 2 diabetes patients | IDF, 2005 |
| 173 | Katchunga, 2010_Democratic Rep | [Hypertension, insulin resistance and chronic kidney disease in type 2 diabetes patients from South Kivu, DR Congo]. | No | Yes | No | Yes | Yes | No | Yes | Yes | Yes | Yes | 7 | Low risk of bias | Type 2 diabetes patients | JIS, 2009 |
| 174 | Katoto, 2018_Democratic Republic | Prevalence and risk factors of metabolic syndrome in HIV-infected adults at three urban clinics in a post-conflict setting, eastern Democratic Republic of the Congo. | No | Yes | No | Yes | Yes | No | Yes | Yes | Unclear | Yes | 6 | Moderate risk of bias | HIV infected patients | IDF, 2005 |
| 175 | Kelliny, 2008_Seychelles | Metabolic syndrome according to different definitions in a rapidly developing country of the African region. | No | Yes | No | Yes | Yes | No | Yes | Yes | Unclear | Yes | 6 | Moderate risk of bias | Apparently healthy individuals | IDF, 2005 |
| 176 | Kengne, 2012_Cameroon | Metabolic syndrome in type 2 diabetes: comparative prevalence according to two sets of diagnostic criteria in sub-Saharan Africans. | No | Yes | No | Yes | Yes | Yes | Yes | Yes | Yes | Yes | 8 | Low risk of bias | Type 2 diabetes patients | IDF, 2005 |
| 177 | Ker, 2007_South Africa | Frequency of the metabolic syndrome in screened South African corporate executives. | No | Yes | No | Yes | Yes | No | Yes | Yes | Unclear | Yes | 6 | Moderate risk of bias | Apparently healthy individuals | NCEP-ATP III, 2001 |

| | | | | | | | | | | | | | | | | |
|---|---|---|---|---|---|---|---|---|---|---|---|---|---|---|---|---|
| 178 | Kerie, 2019_Ethiopia | Metabolic syndrome among residents of Mizan-Aman town, South West Ethiopia, 2017: A cross sectional study. | No | Yes | No | Yes | Yes | Yes | Yes | Yes | Unclear | Yes | 7 | Low risk of bias | Apparently healthy individuals | NCEP-ATP III, 2001 |
| 179 | Khalfa, 2017_Algeria | Prevalence of metabolic syndrome and its association with lifestyle and cardiovascular biomarkers among postmenopausal women in western Algeria. | No | Yes | No | Yes | Yes | No | Yes | Yes | Unclear | Yes | 6 | Moderate risk of bias | Postmenopausal women | NCEP-ATP III, 2001 |
| 180 | Kiama, 2018_Kenya | Prevalence and factors associated with metabolic syndrome in an urban population of adults living with HIV in Nairobi, Kenya. | No | Yes | Yes | Yes | Yes | Yes | Yes | Yes | No | Yes | 8 | Low risk of bias | HIV infected patients | JIS, 2009 |
| 181 | Kingery, 2016_Tanzania | Short-term and long-term cardiovascular risk, metabolic syndrome and HIV in Tanzania. | No | Yes | No | Yes | Yes | No | Yes | Yes | No | Yes | 6 | Moderate risk of bias | Apparently healthy individuals | IDF, 2005 |
| 182 | Kingery, 2016_Tanzania | Short-term and long-term cardiovascular risk, metabolic syndrome and HIV in Tanzania. | No | Yes | No | Yes | Yes | No | Yes | Yes | No | Yes | 6 | Moderate risk of bias | HIV infected patients | IDF, 2005 |
| 183 | Kingue, 2017_Cameroon | Prevalence of selected cardiometabolic risk factors among adults in urban and semi-urban hospitals in four sub-Saharan African countries. | No | Yes | No | Yes | Yes | No | Yes | Yes | Yes | Yes | 7 | Low risk of bias | Apparently healthy individuals | IDF, 2005 |
| 184 | Kingue, 2017_Democratic Republi | Prevalence of selected cardiometabolic risk factors among adults in urban and semi-urban hospitals in four sub-Saharan African countries. | No | Yes | No | Yes | Yes | No | Yes | Yes | Yes | Yes | 7 | Low risk of bias | Apparently healthy individuals | IDF, 2005 |
| 185 | Kingue, 2017_Madagascar | Prevalence of selected cardiometabolic risk factors among adults in urban and semi-urban hospitals in four sub-Saharan African countries. | No | Yes | No | Yes | Yes | No | Yes | Yes | Yes | Yes | 7 | Low risk of bias | Apparently healthy individuals | IDF, 2005 |
| 186 | Kingue, 2017_Nigeria | Prevalence of selected cardiometabolic risk factors among adults in urban and semi-urban hospitals in four sub-Saharan African countries. | No | Yes | No | Yes | Yes | No | Yes | Yes | Yes | Yes | 7 | Low risk of bias | Apparently healthy individuals | IDF, 2005 |
| 187 | Kruger, 2017_South Africa | The prevalence of the metabolic syndrome in a farm worker community in the Boland district, South Africa. | No | Yes | No | Yes | Yes | No | Yes | Yes | No | Yes | 6 | Moderate risk of bias | Apparently healthy individuals | IDF, 2005 |
| 188 | Kwobah, 2021_Kenya | Prevalence and correlates of metabolic syndrome and its components in adults with psychotic disorders in Eldoret, Kenya. | No | Yes | No | Yes | Yes | No | Yes | Yes | Yes | Yes | 7 | Low risk of bias | Apparently healthy individuals | NCEP-ATP III, 2001 |
| 189 | Kwobah, 2021_Kenya | Prevalence and correlates of metabolic syndrome and its components in adults with psychotic disorders in Eldoret, Kenya. | No | Yes | No | Yes | Yes | No | Yes | Yes | Yes | Yes | 7 | Low risk of bias | Psychiatric patients | NCEP-ATP III, 2001 |
| 190 | Labhardt, 2017_Lesotho | Metabolic syndrome in patients on first-line antiretroviral therapy containing zidovudine or tenofovir in rural Lesotho, Southern Africa. | No | Yes | No | Yes | Yes | No | Yes | Yes | No | Yes | 6 | Moderate risk of bias | HIV infected patients | IDF, 2005 |
| 191 | Lajeunesse-Trempe, 2018_Kenya | Anthropometric measures and their association with risk factors for cardio-metabolic diseases in Kenyan adults. | No | Yes | Yes | Yes | Yes | No | Yes | Yes | No | Yes | 7 | Low risk of bias | Apparently healthy individuals | JIS, 2009 |
| 192 | Lawani, 2009_Nigeria | Prevalence of Metabolic Syndrome in Schizophrenics on Antipsychotics in a Nigerian Psychiatric Hospital. | No | Yes | No | Yes | Yes | No | Yes | Yes | No | Yes | 6 | Moderate risk of bias | Psychiatric patients | NCEP-ATP III, 2001 |
| 193 | Lepira, 2010_Democratic Republic | Metabolic syndrome using national cholesterol education program criteria in black patients with arterial hypertension. | No | Yes | No | Yes | Yes | No | Yes | Yes | Unclear | Yes | 6 | Moderate risk of bias | Hypertensive patients | NCEP-ATP III, 2001 |
| 194 | Longo-Mbenza, 2011_Democratic | The metabolic syndrome in a Congolese population and its implications for metabolic syndrome definitions. | No | Yes | Yes | Yes | Yes | No | Yes | Yes | No | Yes | 7 | Low risk of bias | Type 2 diabetes patients | IDF, 2005 |
| 195 | Maaroganye, 2013_South Africa | The prevalence of metabolic syndrome and its associated factors in long-term patients in a specialist psychiatric hospital in South Africa. | No | Yes | No | Yes | Yes | Unclear | Yes | Yes | Yes | Yes | 7 | Low risk of bias | Psychiatric patients | NCEP-ATP III, 2001 |
| 196 | Magalhães, 2014_Angola | Prevalence of the metabolic syndrome and determination of optimal cut-off values of waist circumference in university employees from Angola. | No | Yes | No | Yes | Yes | No | Yes | Yes | Unclear | Yes | 6 | Moderate risk of bias | Apparently healthy individuals | JIS, 2009 |
| 197 | Maistry, 2018_South Africa | Candidate gene polymorphisms related to lipid metabolism in Asian Indians living in Durban, South Africa. | No | Yes | Yes | Yes | Yes | No | Yes | Yes | Yes | Yes | 8 | Low risk of bias | Apparently healthy individuals | JIS, 2009 |
| 198 | Maoujoud, 2011_Morocco | [Prevalence of metabolic syndrome in chronic haemodialysis patients in Morocco]. | No | Yes | No | Yes | Yes | No | Yes | Yes | No | Yes | 6 | Moderate risk of bias | Patients with chronic kidney diseases | NCEP-ATP III, 2001 |
| 199 | Marbou, 2019_Cameroon | Prevalence of Metabolic Syndrome and Its Components in Bamboutos Division's Adults, West Region of Cameroon. | No | Yes | Yes | Yes | Yes | No | Yes | Yes | Yes | Yes | 8 | Low risk of bias | Apparently healthy individuals | JIS, 2009 |
| 200 | Marceline, 2014_Burkina Faso | [Diagnosis and prevalence of metabolic syndrome in diabetics followed in a context of limited resources: the case of Burkina Faso]. | No | Yes | No | Yes | Yes | No | Yes | Yes | Unclear | Yes | 6 | Moderate risk of bias | Type 2 diabetes patients | JIS, 2009 |
| 201 | Masyuko, 2020_Kenya | Metabolic syndrome and 10-year cardiovascular risk among HIV-positive and HIV-negative adults: A cross-sectional study. | No | Yes | No | Yes | Yes | No | Yes | Yes | No | Yes | 6 | Moderate risk of bias | Apparently healthy individuals | JIS, 2009 |
| 202 | Masyuko, 2020_Kenya | Metabolic syndrome and 10-year cardiovascular risk among HIV-positive and HIV-negative adults: A cross-sectional study. | No | Yes | No | Yes | Yes | No | Yes | Yes | No | Yes | 6 | Moderate risk of bias | HIV infected patients | JIS, 2009 |
| 203 | Matsha, 2009_South Africa | Metabolic syndrome in 10-16-year-old learners from the Western Cape, South Africa: Comparison of the NCEP ATP III and IDF criteria. | No | Yes | Yes | Yes | Yes | No | Yes | Yes | Unclear | Yes | 7 | Low risk of bias | Apparently healthy individuals | NCEP-ATP III, 2001 |
| 204 | Matsha, 2019_South Africa | Visceral and subcutaneous adipose tissue association with metabolic syndrome and its components in a South African population. | No | Yes | No | Yes | Yes | Yes | Yes | Yes | Unclear | Yes | 7 | Low risk of bias | Apparently healthy individuals | JIS, 2009 |
| 205 | Mbugua, 2017_Kenya | Metabolic syndrome and its components among university students in Kenya. | No | Yes | Yes | Yes | Yes | No | Yes | Yes | Unclear | Yes | 7 | Low risk of bias | Apparently healthy individuals | JIS, 2009 |
| 206 | Mbunkah, 2014_Cameroon | Prevalence of metabolic syndrome in human immunodeficiency virus - infected patients from the South-West region of Cameroon, using the adult treatment panel III criteria. | No | Yes | No | Yes | Yes | No | Yes | Yes | No | Yes | 6 | Moderate risk of bias | Apparently healthy individuals | NCEP-ATP III, 2001 |
| 207 | Mbunkah, 2014_Cameroon | Prevalence of metabolic syndrome in human immunodeficiency virus - infected patients from the South-West region of Cameroon, using the adult treatment panel III criteria. | No | Yes | No | Yes | Yes | No | Yes | Yes | No | Yes | 6 | Moderate risk of bias | HIV infected patients | NCEP-ATP III, 2001 |
| 208 | Mebazaa, 2011_Tunisia | Metabolic syndrome in Tunisian psoriatic patients: prevalence and determinants. | No | Yes | No | Yes | Yes | No | Yes | Yes | Yes | Yes | 7 | Low risk of bias | Apparently healthy individuals | NCEP-ATP III, 2001 |
| 209 | Mebazaa, 2011_Tunisia | Metabolic syndrome in Tunisian psoriatic patients: prevalence and determinants. | No | Yes | No | Yes | Yes | No | Yes | Yes | Yes | Yes | 7 | Low risk of bias | Patients with dermatological diseases | NCEP-ATP III, 2001 |
| 210 | Mentoor, 2018_South Africa | Metabolic syndrome and body shape predict differences in health parameters in farm working women. | No | Yes | No | Yes | Yes | No | Yes | Yes | No | Yes | 6 | Moderate risk of bias | Apparently healthy individuals | IDF, 2005 |
| 211 | Meziane, 2016_Morocco | Metabolic syndrome in Moroccan patients with psoriasis. | No | Yes | No | Yes | Yes | No | Yes | Yes | Yes | Yes | 7 | Low risk of bias | Patients with dermatological diseases | IDF, 2005 |
| 212 | Millogo, 2014_Burkina Faso | [Metabolic syndrome in hypertensive patients in the cardiology service Yalgado Ouedraogo of Ouagadougou, Burkina Faso]. | No | Yes | No | Yes | Yes | No | Yes | Yes | Yes | Yes | 7 | Low risk of bias | Hypertensive patients | IDF, 2005 |
| 213 | Mogre, 2014_Ghana | Prevalence, components and associated demographic and lifestyle factors of the metabolic syndrome in type 2 diabetes mellitus. | No | Yes | No | Yes | Yes | No | Yes | Yes | Unclear | Yes | 6 | Moderate risk of bias | Type 2 diabetes patients | IDF, 2005 |
| 214 | Mohamed, 2017_Egypt | RELATION BETWEEN SCHISTOSOME PAST INFECTION AND METABOLIC SYNDROME. | No | Yes | No | Yes | Yes | No | Yes | Yes | Yes | Yes | 7 | Low risk of bias | Apparently healthy individuals | IDF, 2005 |
| 215 | Mohammed el, 2008_Morocco | Prevalence of parameter indicators of obesity and its relationship with metabolic syndrome in urban Moroccan women. | No | Yes | No | Yes | Yes | Yes | Yes | Yes | Unclear | No | 6 | Moderate risk of bias | Apparently healthy individuals | NCEP-ATP III, 2001 |
| 216 | Mokwena, 2022_South Africa | Pre-morbid cardiometabolic risks among South Africans living in informal settlements. | No | Yes | No | Yes | Yes | No | Yes | Yes | No | Yes | 6 | Moderate risk of bias | Apparently healthy individuals | JIS, 2009 |
| 217 | Motala, 2011_South Africa | The prevalence of metabolic syndrome and determination of the optimal waist circumference cutoff points in a rural South african community. | No | Yes | Yes | Yes | Yes | No | Yes | Yes | Unclear | Yes | 7 | Low risk of bias | Apparently healthy individuals | JIS, 2009 |
| 218 | Motuma, 2020_Ethiopia | Metabolic Syndrome Among Working Adults in Eastern Ethiopia. | No | Yes | No | Yes | Yes | Yes | Yes | Yes | No | Yes | 7 | Low risk of bias | Apparently healthy individuals | IDF, 2005 |
| 219 | Moustakim, 2021_Morocco | Association of Metabolic Syndrome and Chronic Kidney Disease in Moroccan Adult Population. | No | Yes | No | Yes | Yes | Unclear | Yes | Yes | Unclear | Yes | 6 | Moderate risk of bias | Apparently healthy individuals | NCEP-ATP III, 2001 |
| 220 | Muchanga Sifa, 2014_Democratic | Prevalence and predictors of metabolic syndrome among Congolese pre- and postmenopausal women. | No | Yes | No | Yes | Yes | Yes | Yes | Yes | No | Yes | 7 | Low risk of bias | Apparently healthy individuals | NCEP-ATP III, 2001 |
| 221 | Muhammad, 2017_Nigeria | Metabolic syndrome among HIV infected patients: A comparative cross sectional study in northwestern Nigeria. | No | Yes | No | Yes | Yes | No | Yes | Yes | Unclear | Yes | 6 | Moderate risk of bias | HIV infected patients | NCEP-ATP III, 2001 |
| 222 | Mustafa, 2021_Egypt | Metabolic syndrome in androgenetic alopecia patients; Is serum regulated on activation, normal T-cell expressed and secreted the missing link? | No | Yes | No | Yes | Yes | No | Yes | Yes | Unclear | Yes | 6 | Moderate risk of bias | Apparently healthy individuals | NCEP-ATP III, 2001 |
| 223 | Mustafa, 2021_Egypt | Metabolic syndrome in androgenetic alopecia patients; Is serum regulated on activation, normal T-cell expressed and secreted the missing link? | No | Yes | No | Yes | Yes | No | Yes | Yes | Unclear | Yes | 6 | Moderate risk of bias | Patients with dermatological diseases | NCEP-ATP III, 2001 |
| 224 | Muyanja, 2016_Uganda | High Prevalence of Metabolic Syndrome and Cardiovascular Disease Risk Among People with HIV on Stable ART in Southwestern Uganda. | No | Yes | No | Yes | Yes | No | Yes | Yes | Unclear | Yes | 6 | Moderate risk of bias | HIV infected patients | Revised NCEP-ATP III, 2005 |
| 225 | Nalado, 2015_Nigeria | PREVALENCE OF METABOLIC SYNDROME AMONG APPARENTLY HEALTHY ADULTS IN A RURAL COMMUNITY, IN NORTH-WESTERN NIGERIA. | No | Yes | Yes | Yes | Yes | Unclear | Yes | Yes | Unclear | Yes | 7 | Low risk of bias | Apparently healthy individuals | NCEP-ATP III, 2001 |
| 226 | Ngatchou, 2013_Cameroon | Increased burden and severity of metabolic syndrome and arterial stiffness in treatment-naïve HIV+ patients from Cameroon. | No | Yes | No | Yes | Yes | No | Yes | Yes | No | Yes | 6 | Moderate risk of bias | Apparently healthy individuals | Revised NCEP-ATP III, 2005 |
| 227 | Ngatchou, 2013_Cameroon | Increased burden and severity of metabolic syndrome and arterial stiffness in treatment-naïve HIV+ patients from Cameroon. | No | Yes | No | Yes | Yes | No | Yes | Yes | No | Yes | 6 | Moderate risk of bias | HIV infected patients | Revised NCEP-ATP III, 2005 |
| 228 | Ngoude, 2021_Cameroon | Relationship between periodontal diseases and newly-diagnosed metabolic syndrome components in a sub-Saharan population: a cross sectional study. | No | Yes | No | Yes | Yes | Yes | Yes | Yes | No | Yes | 7 | Low risk of bias | Patients with chronic diseases | IDF, 2005 |
| 229 | N'Guetta, 2016_Côte d'Ivoire | [Prevalence and characteristics of metabolic syndrome among hypertensive patients in Abidjan]. | No | Yes | No | Yes | Yes | Yes | Yes | Yes | No | Yes | 7 | Low risk of bias | Hypertensive patients | IDF, 2005 |
| 230 | Nguyen, 2017_South Africa | Metabolic Syndrome in People Living with Human Immunodeficiency Virus: An Assessment of the Prevalence and the Agreement between Diagnostic Criteria. | No | Yes | No | Yes | Yes | No | Yes | Yes | Yes | Yes | 7 | Low risk of bias | HIV infected patients | JIS, 2009 |
| 231 | Nibouche, 2016_Algeria | [Arterial hypertension at the time of diagnosis of type 2 diabetes in adults]. | No | Yes | No | Yes | Yes | Yes | Yes | Yes | Unclear | Yes | 7 | Low risk of bias | Type 2 diabetes patients | NCEP-ATP III, 2001 |
| 232 | Nibouche-Hattab, 2017_South Afri | Orthostatic hypertension in normotensive type 2 diabetics: What characteristics? | No | Yes | No | Yes | Yes | No | Yes | Yes | Yes | Yes | 7 | Low risk of bias | Type 2 diabetes patients | JIS, 2009 |
| 233 | Nicolaou, 2022_South Africa | Cardiometabolic outcomes of women exposed to hyperglycaemia first detected in pregnancy at 3-6 years post-partum in an urban South African setting. | No | Yes | No | Yes | Yes | Yes | Yes | Yes | No | Yes | 7 | Low risk of bias | Apparently healthy individuals | JIS, 2009 |
| 234 | Nicolaou, 2022_South Africa | Cardiometabolic outcomes of women exposed to hyperglycaemia first detected in pregnancy at 3-6 years post-partum in an urban South African setting. | No | Yes | No | Yes | Yes | Yes | Yes | Yes | No | Yes | 7 | Low risk of bias | Type 2 diabetes patients | JIS, 2009 |
| 235 | Nsiah, 2015_Ghana | Prevalence of metabolic syndrome in type 2 diabetes mellitus patients. | No | Yes | No | Yes | Yes | No | Yes | Yes | No | Yes | 6 | Moderate risk of bias | Type 2 diabetes patients | NCEP-ATP III, 2001 |
| 236 | Ntandou, 2009_Benin | Abdominal obesity explains the positive rural-urban gradient in the prevalence of the metabolic syndrome in Benin, West Africa. | No | Yes | Yes | Yes | Yes | No | Yes | Yes | Unclear | Yes | 7 | Low risk of bias | Apparently healthy individuals | IDF, 2005 |
| 237 | Ntyintyane, 2006_South Africa | Metabolic syndrome, undiagnosed diabetes mellitus and insulin resistance are highly prevalent in urbanised South African blacks with coronary artery disease. | No | Yes | No | Yes | Yes | No | Yes | Yes | Unclear | Yes | 6 | Moderate risk of bias | Patients with coronary artery disease | NCEP-ATP III, 2001 |
| 238 | Ntyintyane, 2008_South Africa | Postprandial lipaemia, metabolic syndrome and LDL particle size in urbanised South African blacks with and without coronary artery disease. | No | Yes | No | Yes | Yes | No | Yes | Yes | Unclear | Yes | 6 | Moderate risk of bias | Apparently healthy individuals | IDF, 2005 |

| | | | | | | | | | | | | | | | | |
|---|---|---|---|---|---|---|---|---|---|---|---|---|---|---|---|---|
| 239 | Ntyintyane, 2008_South Africa | Postprandial lipaemia, metabolic syndrome and LDL particle size in urbanised South African blacks with and without coronary artery disease. | No | Yes | No | Yes | Yes | No | Yes | Yes | Unclear | Yes | 6 | Moderate risk of bias | Patients with coronary artery disease | IDF, 2005 |
| 240 | Ntyintyane, 2009_South Africa | Leptin, adiponectin, and high-sensitivity C-reactive protein in relation to the metabolic syndrome in urban South African blacks with and without coronary artery disease. | No | Yes | No | Yes | Yes | No | Yes | Yes | Unclear | Yes | 6 | Moderate risk of bias | Apparently healthy individuals | IDF, 2005 |
| 241 | Ntyintyane, 2009_South Africa | Leptin, adiponectin, and high-sensitivity C-reactive protein in relation to the metabolic syndrome in urban South African blacks with and without coronary artery disease. | No | Yes | No | Yes | Yes | No | Yes | Yes | Unclear | Yes | 6 | Moderate risk of bias | Patients with coronary artery disease | IDF, 2005 |
| 242 | Nwankwo, 2019_Nigeria | Association between high blood pressure with risk of type 2 diabetes, metabolic syndrome and its predictors: A cross-sectional study. | No | Yes | No | Yes | Yes | No | Yes | Yes | Yes | Yes | 7 | Low risk of bias | Patients with chronic diseases | NCEP-ATP III, 2001 |
| 243 | Obirikorang, 2016_Ghana | Prevalence of metabolic syndrome among HIV-infected patients in Ghana: A cross-sectional study. | No | Yes | No | Yes | Yes | No | Yes | Yes | No | Yes | 6 | Moderate risk of bias | HIV infected patients | NCEP-ATP III, 2001 |
| 244 | Obirikorang, 2018_Ghana | Association of Wrist Circumference and Waist-to-Height Ratio with Cardiometabolic Risk Factors among Type II Diabetics in a Ghanaian Population. | No | Yes | No | Yes | Yes | Yes | Yes | Yes | Unclear | Yes | 7 | Low risk of bias | Apparently healthy individuals | AACE, 2003 |
| 245 | Obirikorang, 2018_Ghana | Association of Wrist Circumference and Waist-to-Height Ratio with Cardiometabolic Risk Factors among Type II Diabetics in a Ghanaian Population. | No | Yes | No | Yes | Yes | Yes | Yes | Yes | Unclear | Yes | 7 | Low risk of bias | Type 2 diabetes patients | IDF, 2005 |
| 246 | Odum, 2018_Nigeria | Elevated cardiac troponin I, creatine kinase and myoglobin and their relationship with cardiovascular risk factors in patients with type 2 diabetes. | No | Yes | No | Yes | Yes | No | Yes | Yes | Unclear | Yes | 6 | Moderate risk of bias | Type 2 diabetes patients | Revised NCEP-ATP III, 2005 |
| 247 | Ogbera, 2010_Nigeria | Prevalence and gender distribution of the metabolic syndrome. | No | Yes | No | Yes | Yes | No | Yes | Yes | Unclear | Yes | 6 | Moderate risk of bias | Type 2 diabetes patients | JIS, 2009 |
| 248 | Ogbera, 2010_Nigeria | Hyperuricaemia and the metabolic syndrome in type 2 DM. | No | Yes | No | Yes | Yes | No | Yes | Yes | No | Yes | 6 | Moderate risk of bias | Type 2 diabetes patients | JIS, 2009 |
| 249 | Ogbera, 2011_Nigeria | Menopausal symptoms and the metabolic syndrome in Nigerian women with type 2 diabetes mellitus. | No | Yes | No | Yes | Yes | No | Yes | Yes | Unclear | Yes | 6 | Moderate risk of bias | Type 2 diabetes patients | NCEP-ATP III, 2001 |
| 250 | Ogbera, 2011_Nigeria | Relationship between serum testosterone levels and features of the metabolic syndrome defining criteria in patients with type 2 diabetes mellitus. | No | Yes | No | Yes | Yes | No | Yes | Yes | No | Yes | 6 | Moderate risk of bias | Type 2 diabetes patients | JIS, 2009 |
| 251 | Ogbera, 2012_Nigeria | The metabolic syndrome in thyroid disease: A report from Nigeria. | No | Yes | No | Yes | Yes | No | Yes | Yes | Unclear | Yes | 6 | Moderate risk of bias | Patients with thyroid disease | JIS, 2009 |
| 252 | Ogbu, 2012_Nigeria | Prevalence of metabolic syndrome using weight and weight indices in an apparently healthy Nigerian population. | No | Yes | No | Yes | Yes | No | Yes | Yes | Unclear | Yes | 6 | Moderate risk of bias | Apparently healthy individuals | NCEP-ATP III, 2001 |
| 253 | Ogbu, 2012_Nigeria | The Prevalence of the Metabolic Syndrome among normal weight Nigerians. | No | Yes | No | Yes | Yes | No | Yes | Yes | Unclear | Yes | 6 | Moderate risk of bias | Apparently healthy individuals | NCEP-ATP III, 2001 |
| 254 | Ogedengbe, 2014_Nigeria | Profile of metabolic abnormalities seen in patients with type 2 diabetes mellitus and their first degree relatives with metabolic syndrome seen in Benin City, Edo state Nigeria. | No | Yes | No | Yes | Yes | No | Yes | Yes | Unclear | Yes | 6 | Moderate risk of bias | Apparently healthy individuals | WHO,1998 |
| 255 | Ogedengbe, 2014_Nigeria | Profile of metabolic abnormalities seen in patients with type 2 diabetes mellitus and their first degree relatives with metabolic syndrome seen in Benin City, Edo state Nigeria. | No | Yes | No | Yes | Yes | No | Yes | Yes | Unclear | Yes | 6 | Moderate risk of bias | Type 2 diabetes patients | WHO,1998 |
| 256 | Oguoma, 2016_Nigeria | Association between metabolic syndrome and 10-year risk of developing cardiovascular disease in a Nigerian population. | No | Yes | Yes | Yes | Yes | No | Yes | Yes | Not applicable | Yes | 7 | Low risk of bias | Apparently healthy individuals | JIS, 2009 |
| 257 | Ojji, 2012_Nigeria | Prevalence of metabolic syndrome among hypertensive patients in Abuja, Nigeria. | No | Yes | No | Yes | Yes | Unclear | Yes | Yes | Unclear | Unclear | 5 | Moderate risk of bias | Hypertensive patients | WHO,1998 |
| 258 | Okpala, 2019_Nigeria | Metabolic Syndrome and Dyslipidemia among Nigerians with Lichen Planus: A Cross-Sectional Study. | No | Yes | Yes | Yes | Yes | No | Yes | Yes | Yes | Yes | 8 | Low risk of bias | Apparently healthy individuals | NCEP-ATP III, 2001 |
| 259 | Okpala, 2019_Nigeria | Metabolic Syndrome and Dyslipidemia among Nigerians with Lichen Planus: A Cross-Sectional Study. | No | Yes | Yes | Yes | Yes | No | Yes | Yes | Yes | Yes | 8 | Low risk of bias | Patients with dermatological diseases | NCEP-ATP III, 2001 |
| 260 | Okpechi, 2007_South Africa | Microalbuminuria and the metabolic syndrome in non-diabetic black Africans. | No | Yes | No | Yes | Yes | No | Yes | Yes | Yes | No | 6 | Moderate risk of bias | Apparently healthy individuals | NCEP-ATP III, 2001 |
| 261 | Okube, 2020_Kenya | Association of dietary patterns and practices on metabolic syndrome in adults with central obesity attending a mission hospital in Kenya: a cross-sectional study. | No | Yes | Yes | Yes | Yes | Yes | Yes | Yes | Unclear | Yes | 8 | Low risk of bias | Patients with chronic diseases | JIS, 2009 |
| 262 | Olokor, 2018_Nigeria | Prevalence of metabolic syndrome among chronic kidney disease patients attending a tertiary hospital in Nigeria – a cross - sectional study. | No | Yes | No | Yes | Yes | No | Yes | Yes | Unclear | Yes | 6 | Moderate risk of bias | Patients with chronic kidney diseases | NCEP-ATP III, 2001 |
| 263 | Omech, 2016_Botswana | Prevalence and determinants of metabolic syndrome: a cross-sectional survey of general medical outpatient clinics using National Cholesterol Education Program-Adult Treatment Panel III criteria in Botswana. | No | Yes | Yes | Yes | Yes | Yes | Yes | Yes | No | Yes | 8 | Low risk of bias | Apparently healthy individuals | NCEP-ATP III, 2001 |
| 264 | Omuse, 2017_Kenya | Metabolic syndrome and its predictors in an urban population in Kenya: A cross sectional study. | No | Yes | No | Yes | Yes | Yes | Yes | Yes | No | Yes | 7 | Low risk of bias | Apparently healthy individuals | JIS, 2009 |
| 265 | Onesi, 2014_Nigeria | Metabolic syndrome: Performance of five different diagnostic criterias. | No | Yes | No | Yes | Yes | No | Yes | Yes | Unclear | Yes | 6 | Moderate risk of bias | Apparently healthy individuals | IDF, 2005 |
| 266 | Onesi, 2014_Nigeria | Metabolic syndrome: Performance of five different diagnostic criterias. | No | Yes | No | Yes | Yes | No | Yes | Yes | Unclear | Yes | 6 | Moderate risk of bias | Type 2 diabetes patients | WHO,1998 |
| 267 | Onyekwere, 2011_Nigeria | Non-alcoholic fatty liver disease and the metabolic syndrome in an urban hospital serving an African community. | No | Yes | No | Yes | Yes | No | Yes | Yes | No | Yes | 6 | Moderate risk of bias | Apparently healthy individuals | JIS, 2009 |
| 268 | Onyekwere, 2011_Nigeria | Non-alcoholic fatty liver disease and the metabolic syndrome in an urban hospital serving an African community. | No | Yes | No | Yes | Yes | No | Yes | Yes | No | Yes | 6 | Moderate risk of bias | Type 2 diabetes patients | JIS, 2009 |
| 269 | Onyenekwu, 2017_Nigeria | Relationship between plasma osteocalcin, glycaemic control and components of metabolic syndrome in adult Nigerians with type 2 diabetes mellitus. | No | Yes | No | Yes | Yes | No | Yes | Yes | Unclear | Yes | 6 | Moderate risk of bias | Apparently healthy individuals | IDF, 2005 |
| 270 | Onyenekwu, 2017_Nigeria | Relationship between plasma osteocalcin, glycaemic control and components of metabolic syndrome in adult Nigerians with type 2 diabetes mellitus. | No | Yes | No | Yes | Yes | No | Yes | Yes | Unclear | Yes | 6 | Moderate risk of bias | Type 2 diabetes patients | IDF, 2005 |
| 271 | Onyenekwu, 2017_Nigeria | The prevalence of metabolic syndrome and its components among overweight and obese Nigerian adolescents and young adults. | No | Yes | No | Yes | Yes | No | Yes | Yes | No | Yes | 6 | Moderate risk of bias | Type 2 diabetes patients | IDF, 2005 |
| 272 | Osei-Yeboah, 2017_Ghana | The Prevalence of Metabolic Syndrome and Its Components among People with Type 2 Diabetes in the Ho Municipality, Ghana: A Cross-Sectional Study. | No | Yes | No | Yes | Yes | No | Yes | Yes | No | Yes | 6 | Moderate risk of bias | Type 2 diabetes patients | IDF, 2005 |
| 273 | Osoti, 2018_Kenya | Metabolic Syndrome Among Antiretroviral Therapy-Naive Versus Experienced HIV-Infected Patients Without Preexisting Cardiometabolic Disorders in Western Kenya. | No | Yes | No | Yes | Yes | No | Yes | Yes | No | No | 5 | Moderate risk of bias | HIV infected patients | IDF, 2005 |
| 274 | Osuji, 2012_Nigeria | Prevalence and characteristics of the metabolic syndrome among newly diagnosed hypertensive patients. | No | Yes | No | Yes | Yes | No | Yes | Yes | Unclear | Yes | 6 | Moderate risk of bias | Hypertensive patients | NCEP-ATP III, 2001 |
| 275 | Osuji, 2012_Nigeria | Metabolic syndrome in newly diagnosed type 2 diabetes mellitus using NCEP-ATP III, the Nnewi experience. | No | Yes | No | Yes | Yes | No | Yes | Yes | Unclear | Yes | 6 | Moderate risk of bias | Type 2 diabetes patients | NCEP-ATP III, 2001 |
| 276 | Osunkwo, 2022_Nigeria | Prevalence and Predictors of Metabolic Syndrome among Adults in North-Central, Nigeria. | No | Yes | No | Yes | Yes | No | Yes | Yes | No | Yes | 6 | Moderate risk of bias | Apparently healthy individuals | NCEP-ATP III, 2001 |
| 277 | Owiredu, 2011_Ghana | The prevalence of metabolic syndrome among active sportsmen/sportswomen and sedentary workers in the Kumasi metropolis. | No | Yes | No | Yes | Yes | No | Yes | Yes | No | Yes | 6 | Moderate risk of bias | Apparently healthy individuals | IDF, 2005 |
| 278 | Owiredu, 2016_Ghana | Co-existence of syndrome X and hypertension among Ghanaians. | No | Yes | No | Yes | Yes | No | Yes | Yes | Yes | Yes | 7 | Low risk of bias | Apparently healthy individuals | IDF, 2005 |
| 279 | Owiredu, 2016_Ghana | Co-existence of syndrome X and hypertension among Ghanaians. | No | Yes | No | Yes | Yes | No | Yes | Yes | Yes | Yes | 7 | Low risk of bias | Hypertensive patients | IDF, 2005 |
| 280 | Owusu-Ansah, 2018_Ghana | Metabolic Syndrome among Schizophrenic Patients: A Comparative Cross-Sectional Study in the Middle Belt of Ghana. | No | Yes | Yes | Yes | Yes | No | Yes | Yes | No | Yes | 7 | Low risk of bias | Psychiatric patients | IDF, 2005 |
| 281 | Paquette, 2017_Kenya | Association Between Plasma Proprotein Convertase Subtilisin/Kexin Type 9 and the Presence of Metabolic Syndrome in a Predominantly Rural-Based Sub-Saharan African Population. | No | Yes | Yes | Yes | Yes | No | Yes | Yes | No | Yes | 7 | Low risk of bias | Apparently healthy individuals | JIS, 2009 |
| 282 | Paruk, 2019_South Africa | Prevalence of low serum testosterone levels among men with type 2 diabetes mellitus attending two outpatient diabetes clinics in KwaZulu-Natal Province, South Africa. | No | Yes | No | Yes | Yes | Yes | Yes | Yes | Yes | Yes | 8 | Low risk of bias | Type 2 diabetes patients | JIS, 2009 |
| 283 | Peer, 2016_South Africa | Differential obesity indices identify the metabolic syndrome in Black men and women in Cape Town: the CRIBSA study. | No | Yes | Yes | Yes | Yes | No | Yes | Yes | Unclear | Yes | 7 | Low risk of bias | Apparently healthy individuals | JIS, 2009 |
| 284 | Pengpid, 2020_Morocco | Prevalence and correlates of the metabolic syndrome in a cross-sectional community-based sample of 18-100 year-olds in Morocco: Results of the first national STEPS survey in 2017. | Yes | Yes | Yes | Yes | Yes | Yes | Yes | Yes | Unclear | Yes | 9 | Low risk of bias | Apparently healthy individuals | JIS, 2009 |
| 285 | Pessinaba, 2013_Senegal | [Prevalence survey of cardiovascular risk factors in the general population in St. Louis (Senegal)]. | No | Yes | No | Yes | Yes | No | Yes | Yes | Unclear | No | 5 | Moderate risk of bias | Apparently healthy individuals | Revised NCEP-ATP III, 2005 |
| 286 | Phalane, 2018_South Africa | The metabolic syndrome and renal function in an African cohort infected with human immunodeficiency virus. | No | Yes | No | Yes | Yes | No | Yes | Yes | Unclear | Yes | 6 | Moderate risk of bias | Apparently healthy individuals | IDF, 2005 |
| 287 | Phalane, 2018_South Africa | The metabolic syndrome and renal function in an African cohort infected with human immunodeficiency virus. | No | Yes | No | Yes | Yes | No | Yes | Yes | Unclear | Yes | 6 | Moderate risk of bias | HIV infected patients | IDF, 2005 |
| 288 | Quaye, 2019_Ghana | Comparative Abilities of Body Mass Index, Waist Circumference, Abdominal Volume Index, Body Adiposity Index, and Conicity Index as Predictive Screening Tools for Metabolic Syndrome among Apparently Healthy Ghanaian Adults. | No | Yes | No | Yes | Yes | Yes | Yes | Yes | No | Yes | 7 | Low risk of bias | Apparently healthy individuals | JIS, 2009 |
| 289 | Raharinavalona, 2020_Madagasca | [Prevalences of metabolic syndrome and cardiovascular risk factors in type 2 diabetics hospitalized in the Department of Endocrinology, Antananarivo]. | No | Yes | No | Yes | Yes | No | Yes | Yes | No | Yes | 6 | Moderate risk of bias | Type 2 diabetes patients | JIS, 2009 |
| 290 | Regaieg, 2018_Tunisia | Metabolic syndrome and physical activity measured by pedometer among adolescents. | No | Yes | No | Yes | Yes | No | Yes | Yes | No | Yes | 6 | Moderate risk of bias | Apparently healthy individuals | IDF, 2005 |
| 291 | Rejeb, 2010_Tunisia | Metabolic syndrome is a risk factor for coronary artery disease in a tunisian population. | No | Yes | No | Yes | Yes | No | Yes | Yes | No | Yes | 6 | Moderate risk of bias | Patients with coronary artery disease | IDF, 2005 |
| 292 | Rguibi, 2004_Morocco | Metabolic syndrome among Moroccan Sahraoui adult Women. | No | Yes | Yes | Yes | Yes | No | Yes | Yes | No | No | 6 | Moderate risk of bias | Apparently healthy individuals | NCEP-ATP III, 2001 |
| 293 | Rguibi, 2007_Morocco | High blood pressure in urban Moroccan Sahraoui women. | No | Yes | Yes | Yes | Yes | No | Yes | Yes | No | No | 6 | Moderate risk of bias | Apparently healthy individuals | NCEP-ATP III, 2001 |
| 294 | Rguibi, 2007_Morocco | High blood pressure in urban Moroccan Sahraoui women. | No | Yes | Yes | Yes | Yes | No | Yes | Yes | No | No | 6 | Moderate risk of bias | Hypertensive patients | NCEP-ATP III, 2001 |
| 295 | Rostom, 2013_Morocco | Metabolic syndrome in rheumatoid arthritis: case control study. | No | Yes | No | Yes | Yes | No | Yes | Yes | Yes | Yes | 7 | Low risk of bias | Apparently healthy individuals | IDF, 2005 |
| 296 | Rostom, 2013_Morocco | Metabolic syndrome in rheumatoid arthritis: case control study. | No | Yes | No | Yes | Yes | No | Yes | Yes | Yes | Yes | 7 | Low risk of bias | Patients with rheumatoid arthritis | IDF, 2005 |
| 297 | Rwegerera, 2021_Botswana | Metabolic Control and Determinants Among HIV-Infected Type 2 Diabetes Mellitus Patients Attending a Tertiary Clinic in Botswana. | No | Yes | No | Yes | Yes | Yes | Yes | Yes | No | Yes | 7 | Low risk of bias | Apparently healthy individuals | IDF, 2005 |

| | | | | | | | | | | | | | | | | |
|---|---|---|---|---|---|---|---|---|---|---|---|---|---|---|---|---|
| 298 | Rwegerera, 2021_Botswana | Metabolic Control and Determinants Among HIV-Infected Type 2 Diabetes Mellitus Patients Attending a Tertiary Clinic in Botswana. | No | Yes | No | Yes | Yes | Yes | Yes | Yes | No | Yes | 7 | Low risk of bias | HIV infected patients | IDF, 2005 |
| 299 | Sabir, 2016_Nigeria | Oxidative stress among subjects with metabolic syndrome in Sokoto, North.Western Nigeria. | No | Yes | Yes | Yes | Yes | No | Yes | Yes | Unclear | Yes | 7 | Low risk of bias | Apparently healthy individuals | NCEP-ATP III, 2001 |
| 300 | Sabir, 2016_Nigeria | Metabolic syndrome in urban city of North-Western Nigeria: prevalence and determinants. | No | Yes | Yes | Yes | Yes | No | Yes | Yes | Unclear | No | 6 | Moderate risk of bias | Apparently healthy individuals | NCEP-ATP III, 2001 |
| 301 | Salawu, 2015_Nigeria | Prevalence of metabolic syndrome and its component traits among students in a Nigerian university. | No | Yes | No | Yes | Yes | Yes | Yes | Yes | Unclear | Yes | 7 | Low risk of bias | Apparently healthy individuals | JIS, 2009 |
| 302 | Salem, 2021_Egypt | Association between Toxoplasma gondii infection and metabolic syndrome in obese adolescents: A possible immune-metabolic link. | No | Yes | No | Yes | Yes | No | Yes | Yes | No | Yes | 6 | Moderate risk of bias | Patients with obesity | IDF, 2005 |
| 303 | Saloojee, 2018_South Africa | Metabolic syndrome in antipsychotic naive African patients with severe mental illness in usual care. | No | Yes | No | Yes | Yes | No | Yes | Yes | Yes | Yes | 7 | Low risk of bias | Apparently healthy individuals | JIS, 2009 |
| 304 | Saloojee, 2018_South Africa | Metabolic syndrome in antipsychotic naive African patients with severe mental illness in usual care. | No | Yes | No | Yes | Yes | No | Yes | Yes | Yes | Yes | 7 | Low risk of bias | Psychiatric patients | JIS, 2009 |
| 305 | Samaan, 2022_Egypt | The Impact of Metabolic Syndrome on Quality of Life Among Individuals With Knee Osteoarthritis Living in Egypt. | No | Yes | No | Yes | Yes | No | Yes | Yes | No | Yes | 6 | Moderate risk of bias | Patients with Chronic musculoskeletal diseases | JIS, 2009 |
| 306 | Sanad, 2011_Egypt | Evaluation of microalbuminuria in obese children and its relation to metabolic syndrome. | No | Yes | No | Yes | Yes | No | Yes | Yes | Yes | Yes | 7 | Low risk of bias | Patients with obesity | NCEP-ATP III, 2001 |
| 307 | Sawadogo, 2014_Burkina Faso | [Metabolic syndrome and cardiovascular risk patients under antiretrovirals in a day hospital at Bobo-Dioulasso (Burkina Faso)]. | No | Yes | No | Yes | Yes | No | Yes | Yes | Unclear | Yes | 6 | Moderate risk of bias | HIV infected patients | NCEP-ATP III, 2001 |
| 308 | Schutte, 2007_South Africa | Metabolic syndrome risk in black South African women compared to Caucasian women. | No | Yes | No | Yes | Yes | No | Yes | Yes | Yes | Yes | 7 | Low risk of bias | Apparently healthy individuals | IDF, 2005 |
| 309 | Schutte, 2009_South Africa | Classifying Africans with the metabolic syndrome. | No | Yes | No | Yes | Yes | No | Yes | Yes | Unclear | Yes | 6 | Moderate risk of bias | Apparently healthy individuals | AACE, 2003 |
| 310 | Sekgala, 2018_South Africa | The risk of metabolic syndrome as a result of lifestyle among Ellisras rural young adults. | No | Yes | No | Yes | Yes | No | Yes | Yes | Unclear | No | 5 | Moderate risk of bias | Apparently healthy individuals | JIS, 2009 |
| 311 | Setroame, 2020_Ghana | Prevalence of Metabolic Syndrome and Nonalcoholic Fatty Liver Disease among Premenopausal and Postmenopausal Women in Ho Municipality: A Cross-Sectional Study. | No | Yes | No | Yes | Yes | Yes | Yes | Yes | No | Yes | 7 | Low risk of bias | Apparently healthy individuals | NCEP-ATP III, 2001 |
| 312 | Setroame, 2020_Ghana | Prevalence of Metabolic Syndrome and Nonalcoholic Fatty Liver Disease among Premenopausal and Postmenopausal Women in Ho Municipality: A Cross-Sectional Study. | No | Yes | No | Yes | Yes | Yes | Yes | Yes | No | Yes | 7 | Low risk of bias | Postmenopausal women | NCEP-ATP III, 2001 |
| 313 | Shaheen, 2012_Egypt | Assessment of serum leptin, insulin resistance and metabolic syndrome in patients with skin tags. | No | Yes | Yes | Yes | Yes | No | Yes | Yes | No | Yes | 7 | Low risk of bias | Apparently healthy individuals | Revised NCEP-ATP III, 2005 |
| 314 | Shaheen, 2012_Egypt | Assessment of serum leptin, insulin resistance and metabolic syndrome in patients with skin tags. | No | Yes | Yes | Yes | Yes | No | Yes | Yes | No | Yes | 7 | Low risk of bias | Patients with dermatological diseases | Revised NCEP-ATP III, 2005 |
| 315 | Siminialayi, 2008_Nigeria | Metabolic Syndrome in a Rural Nigerian Community: Is Central Obesity always the key Determinant? | No | Yes | No | Yes | Yes | No | Yes | Yes | Unclear | Yes | 6 | Moderate risk of bias | Apparently healthy individuals | NCEP-ATP III, 2001 |
| 316 | Slimani, 2017_Algeria | Prevalence of metabolic syndrome in Algerian rheumatoid arthritis patients. Correlation with disease activity and functional status. | No | Yes | No | Yes | Yes | No | Yes | Yes | No | Yes | 6 | Moderate risk of bias | Patients with rheumatoid arthritis | NCEP-ATP III, 2001 |
| 317 | Soliman, 2019_Egypt | The prevalence and the clinical profile of metabolic syndrome in children and adolescents with Type 1 diabetes. | No | Yes | No | Yes | Yes | No | Yes | Yes | Yes | Yes | 7 | Low risk of bias | Patients with type 1 diabetes | IDF, 2005 |
| 318 | Solomon, 2019_Ethiopia | Disease burden and associated risk factors for metabolic syndrome among adults in Ethiopia. | No | Yes | No | Yes | Yes | No | Yes | Yes | No | Yes | 6 | Moderate risk of bias | Patients with chronic diseases | Revised NCEP-ATP III, 2005 |
| 319 | Sossa, 2016_Benin | Performances comparées du HDL-cholestérol et du ratio cholestérol total/HDL pour le dépistage du syndrome métabolique chez des adultes du Sud-Bénin (Afrique de l'Ouest). | No | Yes | Yes | Yes | Yes | Yes | Yes | Yes | Unclear | Yes | 8 | Low risk of bias | Apparently healthy individuals | JIS, 2009 |
| 320 | Tachebele, 2014_Ethiopia | Metabolic syndrome among hypertensive patients at University of Gondar Hospital, North West Ethiopia: a cross sectional study. | No | Yes | Yes | Yes | Yes | Yes | Yes | Yes | No | No | 7 | Low risk of bias | Hypertensive patients | Revised NCEP-ATP III, 2005 |
| 321 | Tadewos, 2017_Ethiopia | Risk factors of metabolic syndrome among hypertensive patients at Hawassa University Comprehensive Specialized Hospital, Southern Ethiopia. | No | Yes | Yes | Yes | Yes | Yes | Yes | Yes | No | Yes | 8 | Low risk of bias | Hypertensive patients | NCEP-ATP III, 2001 |
| 322 | Tesfaye, 2014_Ethiopia | Burden of metabolic syndrome among HIV-infected patients in Southern Ethiopia. | No | Yes | Yes | Yes | Yes | Yes | Yes | Yes | No | No | 7 | Low risk of bias | HIV infected patients | IDF, 2005 |
| 323 | Teshome, 2020_Ethiopia | Prevalence and Associated Factors of Metabolic Syndrome Among Patients with Severe Mental Illness at Hawassa, Southern-Ethiopia. | No | Yes | Yes | Yes | Yes | Yes | Yes | Yes | No | Yes | 8 | Low risk of bias | Psychiatric patients | IDF, 2005 |
| 324 | Titty, 2009_Ghana | Incidence and Major Metabolic Risk Factors of Metabolic Syndrome in Type 2 Diabetic Out-Patients Visiting Tamale Teaching Hospital in Ghana. | No | Yes | No | Yes | Yes | No | Yes | Yes | Yes | Yes | 7 | Low risk of bias | Type 2 diabetes patients | NCEP-ATP III, 2001 |
| 325 | Titty, 2010_Ghana | Glycaemic control, dyslipidaemia and metabolic syndrome among recently diagnosed diabetes mellitus patients in Tamale Teaching Hospital, Ghana. | No | Yes | No | Yes | Yes | No | Yes | Yes | Yes | Yes | 7 | Low risk of bias | Type 2 diabetes patients | NCEP-ATP III, 2001 |
| 326 | Tladi, 2021_Botswana | Prevalence of the Metabolic Syndrome Among Batswana Adults in Urban and Semi-Urban Gaborone. | No | Yes | Yes | Yes | Yes | No | Yes | Yes | Unclear | No | 6 | Moderate risk of bias | Apparently healthy individuals | IDF, 2005 |
| 327 | Tran, 2011_Ethiopia | Prevalence of Metabolic Syndrome among Working Adults in Ethiopia. | No | Yes | Yes | Yes | Yes | Yes | Yes | Yes | No | No | 7 | Low risk of bias | Apparently healthy individuals | IDF, 2005 |
| 328 | Udenze, 2013_Nigeria | The prevalence of metabolic syndrome in persons with type 2 diabetes at the Lagos University Tteaching Hospital, Lagos, Nigeria. | No | Yes | No | Yes | Yes | No | Yes | Yes | Unclear | Yes | 6 | Moderate risk of bias | Type 2 diabetes patients | WHO,1998 |
| 329 | Ulasi, 2010_Nigeria | A community-based study of hypertension and cardio-metabolic syndrome in semi-urban and rural communities in Nigeria. | No | Yes | Yes | Yes | Yes | No | Yes | Yes | Unclear | No | 6 | Moderate risk of bias | Apparently healthy individuals | IDF, 2005 |
| 330 | Unadike, 2009_Nigeria | Prevalence of the metabolic syndrome among patients with type 2 diabetes mellitus in Uyo, Nigeria. | No | Yes | No | Yes | Yes | No | Yes | Yes | No | Yes | 6 | Moderate risk of bias | Type 2 diabetes patients | NCEP-ATP III, 2001 |
| 331 | Uwanuruochi, 2013_Nigeria | Cardiovascular risk factors in adult staff of Federal Medical Centre, Umuahia: a comparison with other Nigerian studies. | No | Yes | No | Yes | Yes | No | Yes | Yes | No | Yes | 6 | Moderate risk of bias | Apparently healthy individuals | IDF, 2005 |
| 332 | van der Heijden, 2021_Ghana | The Magnitude and Directions of the Associations between Early Life Factors and Metabolic Syndrome Differ across Geographical Locations among Migrant and Non-Migrant Ghanaians-The RODAM Study. | No | Yes | No | Yes | Yes | No | Yes | Yes | Yes | Yes | 7 | Low risk of bias | Apparently healthy individuals | JIS, 2009 |
| 333 | Veldsman, 2022_South Africa | The relationship between physical activity, body fatness and metabolic syndrome in urban South African school teachers: The sympathetic activity and ambulatory blood pressure in Africans study. | No | Yes | No | Yes | Yes | No | Yes | Yes | Yes | Yes | 7 | Low risk of bias | Apparently healthy individuals | JIS, 2009 |
| 334 | Wahab, 2008_Nigeria | Frequency and determinants of the metabolic syndrome in apparently healthy adult Nigerians. | No | Yes | No | Yes | Yes | No | Yes | Yes | No | Yes | 6 | Moderate risk of bias | Apparently healthy individuals | NCEP-ATP III, 2001 |
| 335 | Woldeyes, 2022_Ethiopia | Prevalence of Clinical Cardiovascular Disease Risk Factors Among HIV Infected Patients on Anti-Retroviral Treatment in a Tertiary Hospital in Ethiopia. | No | Yes | No | Yes | Yes | No | Yes | Yes | No | Yes | 6 | Moderate risk of bias | HIV infected patients | NCEP-ATP III, 2001 |
| 336 | Woldu, 2022_Ethiopia | Biomarkers and Prevalence of Cardiometabolic Syndrome Among People Living With HIV/AIDS, Addis Ababa, Ethiopia: A Hospital-Based Study. | No | Yes | No | Yes | Yes | Yes | Yes | Yes | Yes | Yes | 8 | Low risk of bias | HIV infected patients | IDF, 2005 |
| 337 | Workalemahu, 2013_Ethiopia | Physical activity and metabolic syndrome among Ethiopian adults. | No | Yes | Yes | Yes | Yes | No | Yes | Yes | No | Yes | 7 | Low risk of bias | Apparently healthy individuals | IDF, 2005 |
| 338 | Woyesa, 2017_Ethiopia | Hyperuricemia and metabolic syndrome in type 2 diabetes mellitus patients at Hawassa university comprehensive specialized hospital, South West Ethiopia. | No | Yes | No | Yes | Yes | Yes | Yes | Yes | No | Yes | 7 | Low risk of bias | Type 2 diabetes patients | NCEP-ATP III, 2001 |
| 339 | Yasir, 2016_Sudan | Metabolic syndrome and its association with obesity and lifestyle factors in Sudanese population. | No | Yes | Yes | Yes | Yes | Yes | Yes | Yes | Yes | Yes | 9 | Low risk of bias | Apparently healthy individuals | NCEP-ATP III, 2001 |
| 340 | Yeboah, 2017_Ghana | Metabolic syndrome and parental history of cardiovascular disease in young adults in urban Ghana. | No | Yes | No | Yes | Yes | No | Yes | Yes | No | Yes | 6 | Moderate risk of bias | Apparently healthy individuals | JIS, 2009 |
| 341 | Yerima, 2017_Nigeria | Knee osteoarthritis and associated cardio-metabolic clusters in a tertiary hospital in Nigeria. | No | Yes | No | Yes | Yes | Yes | Yes | Yes | No | Yes | 7 | Low risk of bias | Patients with rheumatoid arthritis | IDF, 2005 |
| 342 | Young, 2016_Nigeria | Insulin resistance, metabolic syndrome, and lipids in African women. | No | Yes | No | Yes | Yes | No | Yes | Yes | Unclear | Yes | 6 | Moderate risk of bias | Apparently healthy individuals | JIS, 2009 |
| 343 | Zaki, 2015_Egypt | Indicators of the metabolic syndrome in obese adolescents. | No | Yes | No | Yes | Yes | No | Yes | Yes | Yes | Yes | 7 | Low risk of bias | Patients with obesity | IDF, 2005 |
| 344 | Zeba, 2012_Burkina Faso | The double burden of malnutrition and cardiometabolic risk widens the gender and socio-economic health gap: a study among adults in Burkina Faso (West Africa). | No | Yes | Yes | Yes | Yes | Yes | Yes | Yes | Unclear | No | 7 | Low risk of bias | Apparently healthy individuals | WHO,1998 |
| 345 | Zerga, 2020_Ethiopia | Metabolic syndrome and lifestyle factors among type 2 diabetes mellitus patients in Dessie Referral Hospital, Amhara region, Ethiopia. | No | Yes | No | Yes | Yes | Yes | Yes | Yes | No | Yes | 7 | Low risk of bias | Type 2 diabetes patients | JIS, 2009 |
