# Supplementary material for: Prevalence of the metabolic syndrome in African populations: A systematic review and meta-analysis
Source: PLoS One. 2023 Jul 27;18(7):e0289155. doi: 10.1371/journal.pone.0289155 (PMC10374159; doi:10.1371/journal.pone.0289155)
Supplement: S7 Table — (PDF) [file pone.0289155.s009.pdf]

S7 Table. Subgroup analyses of global prevalence of metabolic syndrome in Africa.

	Prevalence. % (95%CI)	95% Prediction interval	N Studies	N Participants	H (95%CI)	I² (95%CI)	P heterogeneity	P difference subtypes
**Metabolic syndrome in Africa**								
**Study Design**								0.588
Cross-sectional	32.7 [30.4-35.1]	[2.5-75.5]	318	152335	9.8 [9.6-10]	99 [98.9-99]	< 0.001	
Case control	30.6 [23.2-38.6]	[3.3-69.3]	20	3127	4.6 [4-5.3]	95.3 [93.9-96.4]	< 0.001	
Cohort (Baseline data)	22.7 [6.5-44.7]	[0-95.9]	7	1002	7.1 [6-8.5]	98 [97.2-98.6]	< 0.001	
**Sampling**								0.007
Non probabilistic	33.8 [31.2-36.4]	[2.5-77.5]	284	102016	8.7 [8.5-8.9]	98.7 [98.6-98.7]	< 0.001	
Probabilistic	26.4 [22-31.1]	[1.5-66.3]	61	54448	12 [11.5-12.5]	99.3 [99.2-99.4]	< 0.001	
**Setting**								< 0.001
Hospital-based	36.6 [33.7-39.6]	[2.6-82]	248	65375	7.9 [7.7-8.1]	98.4 [98.3-98.5]	< 0.001	
Community-based	22.1 [19.2-25.1]	[2.1-54.4]	91	79705	10 [9.6-10.4]	99 [98.9-99.1]	< 0.001	
**Timing of samples collection**								0.045
Prospetively	32.7 [30.4-35]	[2.4-75.7]	337	151603	9.6 [9.4-9.8]	98.9 [98.9-99]	< 0.001	
Retrospectively	22.2 [13.6-32.2]	[0.4-62]	8	4861	7.6 [6.5-8.9]	98.3 [97.6-98.7]	< 0.001	
**Countries**								0.009
Algeria	43.9 [19.3-70.2]	[0-100]	9	5311	19 [17.5-20.6]	99.7 [99.7-99.8]	< 0.001	
Ghana	39.7 [31.2-48.5]	[1.9-88.3]	34	9930	8.7 [8.1-9.3]	98.7 [98.5-98.8]	< 0.001	
South Africa	34.1 [28.2-40.2]	[2.7-77.4]	49	16770	8.1 [7.6-8.6]	98.5 [98.3-98.6]	< 0.001	
Egypt	33.2 [26.4-40.3]	[3.3-74.2]	31	9999	6.7 [6.1-7.2]	97.7 [97.3-98.1]	< 0.001	
Uganda	32.8 [12-57.9]	[0-100]	3	713	6.8 [5-9.4]	97.8 [96-98.9]	< 0.001	
Ethiopia	32.6 [26.6-38.9]	[3.7-72.5]	36	28329	10.6 [10-11.3]	99.1 [99-99.2]	< 0.001	
Democratic Republic of the Congo	32.3 [23.9-41.4]	[6.8-65.5]	6	2398	4.1 [3.1-5.4]	94 [89.6-96.6]	< 0.001	
Nigeria	32.3 [26.9-38.1]	[1.1-79.3]	66	21326	8.6 [8.2-9.1]	98.7 [98.5-98.8]	< 0.001	
Morocco	31.9 [24.7-39.5]	[5.4-67.4]	18	8499	6.2 [5.5-7]	97.4 [96.8-98]	< 0.001	
Tunisia	31.8 [24.9-39.1]	[2.7-73.4]	29	20972	10.5 [9.9-11.2]	99.1 [99-99.2]	< 0.001	
Botswana	30.9 [26.3-35.6]	[16.9-46.8]	5	1592	1.8 [1.1-2.8]	68.1 [17.6-87.6]	0.014	
Cameroon	30.6 [17.7-45.1]	[0-87.8]	13	4249	9.3 [8.3-10.3]	98.8 [98.6-99.1]	< 0.001	
Seychelles	29.2 [21.5-37.5]	[0-100]	3	3728	5.5 [3.8-7.9]	96.7 [93.2-98.4]	< 0.001	
Angola	28 [19.1-37.8]	[0-100]	3	1895	4.6 [3.1-6.9]	95.4 [89.7-97.9]	< 0.001	
Kenya	20.6 [10.6-32.9]	[0-74.4]	12	6358	11 [10-12.2]	99.2 [99-99.3]	< 0.001	
Burkina Faso	18.2 [8.9-29.9]	[0-66.2]	6	5634	7.9 [6.6-9.5]	98.4 [97.7-98.9]	< 0.001	

	Prevalence. % (95%CI)	95% Prediction interval	N Studies	N Participants	H (95%CI)	I² (95%CI)	P heterogeneity	P difference subtypes
Sudan	11.5 [5.1-19.9]	[0-47.2]	5	895	2.9 [2-4.2]	87.7 [73.9-94.3]	< 0.001	
**WHO Region**								0.713
Africa	32.6 [29.9-35.4]	[1.9-77.2]	260	115909	9.9 [9.7-10.1]	99 [98.9-99]	< 0.001	
Eastern Mediterranean	31.7 [27.8-35.7]	[4.1-69.6]	85	40555	8.3 [7.9-8.6]	98.5 [98.4-98.7]	< 0.001	
**UNSD Region**								0.835
Southern Africa	33.6 [28.3-39.1]	[3.4-75]	55	19528	7.9 [7.5-8.3]	98.4 [98.2-98.6]	< 0.001	
West Africa	33.1 [28.7-37.7]	[1-80.9]	112	40534	9.6 [9.2-9.9]	98.9 [98.8-99]	< 0.001	
Northern Africa	32.8 [28.6-37.3]	[2.4-76]	94	45866	9.7 [9.3-10.1]	98.9 [98.9-99]	< 0.001	
Eastern Africa	30.3 [25.6-35.2]	[2.7-70.6]	61	41868	10.5 [10.1-11]	99.1 [99-99.2]	< 0.001	
Central Africa	30.1 [22.9-37.8]	[2.5-70.6]	23	8668	7.5 [6.8-8.2]	98.2 [97.8-98.5]	< 0.001	
**Country income level**								0.437
Upper-middle-income economies	35 [29.5-40.6]	[3.5-77.1]	56	18552	7.8 [7.4-8.3]	98.4 [98.2-98.5]	< 0.001	
Lower-middle income economies	32.6 [29.7-35.5]	[2-76.8]	225	94578	9.5 [9.3-9.7]	98.9 [98.8-98.9]	< 0.001	
Low-income economies	29.7 [25.2-34.5]	[3-68.6]	61	39606	9.8 [9.3-10.3]	99 [98.9-99]	< 0.001	
High-income economies	29.2 [21.5-37.5]	[0-100]	3	3728	5.5 [3.8-7.9]	96.7 [93.2-98.4]	< 0.001	
**Age range**								< 0.001
Adults: 18+ years	33.1 [30.8-35.5]	[4.8-71.1]	257	121647	8.6 [8.4-8.8]	98.6 [98.6-98.7]	< 0.001	
Children : Birth-18 years	13.3 [7.3-20.6]	[0-53.6]	18	5722	6.9 [6.1-7.7]	97.9 [97.3-98.3]	< 0.001	
**Gender**								<0.001
Female	36.9 [33.2-40.7]	[2.9-81.7]	151	52057	8.8 [8.6-9.1]	98.7 [98.6-98.8]	<0.001	
Male	26.7 [23.1-30.5]	[0.3-71.8]	128	32460	7.4 [7.1-7.7]	98.2 [98-98.3]	<0.001	
**Population categories**								< 0.001
Type 2 diabetes patients	66.9 [60.3-73.1]	[21.3-98.3]	46	13063	7.9 [7.4-8.4]	98.4 [98.2-98.6]	< 0.001	
Patients with coronary artery disease	55.2 [50.8-59.6]	[48-62.3]	5	504	1 [1-2.2]	0 [0-79.2]	0.956	
Patients with cardiovascular diseases	48.3 [33.5-63.3]	[0-100]	3	333	2.6 [1.5-4.5]	85.6 [58-95.1]	0.001	
Patients with Chronic musculoskeletal diseases	48.3 [39-57.6]	[0-100]	3	350	1.7 [1-3.2]	65.1 [0-90]	0.057	
Patients with chronic kidney diseases	46.7 [32.7-60.9]	[4.2-92.6]	5	556	3.2 [2.3-4.6]	90.5 [80.8-95.3]	< 0.001	
Postmenopausal women	45.4 [34.9-56.1]	[11.5-82]	8	2816	5.2 [4.2-6.4]	96.3 [94.4-97.5]	< 0.001	
Patients with respiratory tract diseases	44 [16.4-73.6]	[0-100]	3	528	6.4 [4.6-8.8]	97.5 [95.2-98.7]	< 0.001	
Hypertensive patients	43.8 [36.4-51.4]	[13.4-77.1]	18	5782	5.5 [4.9-6.3]	96.7 [95.8-97.5]	< 0.001	
Patients with dermatological diseases	41.6 [29.5-54.4]	[3.9-86.8]	11	1382	4.6 [3.8-5.5]	95.3 [93.2-96.7]	< 0.001	
Patients with rheumatoid arthritis	37.8 [20.7-56.6]	[0-97.5]	5	922	5.8 [4.5-7.4]	97 [95.1-98.2]	< 0.001	
Patients with chronic diseases	33 [13.7-55.9]	[0-99.3]	6	5410	11.7 [10.2-13.5]	99.3 [99-99.5]	< 0.001	

	Prevalence. % (95%CI)	95% Prediction interval	N Studies	N Participants	H (95%CI)	I^2 (95%CI)	P heterogeneity	P difference subtypes
Patients with obesity	31.5 [22-41.7]	[3.2-71.3]	11	1413	3.9 [3.2-4.8]	93.6 [90.3-95.7]	< 0.001	
Psychiatric patients	26.2 [22.3-30.3]	[13.2-41.7]	13	2329	2.1 [1.6-2.7]	77.4 [61.7-86.7]	< 0.001	
HIV infected patients	22.6 [18.6-26.9]	[4.1-49.9]	32	11787	5.4 [4.9-5.9]	96.6 [95.8-97.2]	< 0.001	
Apparently healthy individuals	22.2 [19.9-24.5]	[1.8-55.6]	167	107532	8.9 [8.7-9.2]	98.7 [98.7-98.8]	< 0.001	
**MS definition**								0.125
WHO, 1998	44.8 [24.8-65.7]	[0-100]	12	4786	14 [12.9-15.3]	99.5 [99.4-99.6]	< 0.001	
Revised NCEP-ATP III, 2005	39.7 [31.7-48.1]	[4-84]	29	13261	9.3 [8.7-10]	98.8 [98.7-99]	< 0.001	
JIS, 2009	33.1 [28.5-37.8]	[2.7-75.7]	78	40973	10 [9.6-10.4]	99 [98.9-99.1]	< 0.001	
NCEP-ATP III, 2001	31.6 [27.8-35.6]	[2.4-73.8]	109	47098	9 [8.7-9.3]	98.8 [98.7-98.8]	< 0.001	
IDF, 2005	29.3 [25.7-33]	[1.9-70.7]	115	49868	8.7 [8.4-9.1]	98.7 [98.6-98.8]	< 0.001	
