# Supplementary material for: Prevalence of the metabolic syndrome in African populations: A systematic review and meta-analysis
Source: PLoS One. 2023 Jul 27;18(7):e0289155. doi: 10.1371/journal.pone.0289155 (PMC10374159; doi:10.1371/journal.pone.0289155)
Supplement: Attachment — Submitted filename: Response to Reviewers.doc [file pone.0289155.s010.doc]

**Review Comments to the Author**

**Reviewer #1:** In this manuscript, Bowo-Ngandji et al. conducted a systematic review and meta-analysis to assess the prevalence of metabolic syndrome in African population. They separately analyzed studies using the WHO, revised NCEP-ATP III, JIS, NCEP/ATP III and IDF definition criteria. This is a high-quality and comprehensive study. The statistical analysis sounds appropriate.

Authors: We thank the reviewer for this appreciation.

1. In the abstract, the authors mention “and type 2 diabetics were the most affected,with a prevalence of 66.9%”.The comparison seems to be incomplete.

Authors: Thank you for your valuable feedback. We agree with your observation and have now completed the	comparison in abstract.

2. I suggest that authors spell out numbers smaller than ten in the introduction.

Authors: Thank you for your valuable feedback. We agree with your suggestion and have now included in the introduction.

3. It should be “Joint Interim Statement not” “Join Interim Statement”

Authors: Thank you for your valuable feedback. We agree with your suggestion and have now included on line 79

**Reviewer #2:** Examining the overall prevalence of metabolic syndrome in Africa has been previously reported in various studies as the authors have mentioned, but overall reporting and meta-analysis have not been done before, which is important in this study.

Authors: We thank the reviewer for this summary and appreciation.

1. It would be better to report the prevalence by gender, too.

Authors: Thank you for this valuable feedback on our manuscript. We have provided more information regarding gender distribution in abstract, results, and discussion.

2. In the distribution of countries, it is good to include the names of the countries.

Authors: Thank you for your thorough review and for bringing to our attention to include the names of the countries in the distribution of countries in our article. We have revised our manuscript to include all the names of the countries in the distribution of countries.
