# Supplementary material for: Prevalence of the metabolic syndrome in African populations: A systematic review and meta-analysis
Source: PLoS One. 2023 Jul 27;18(7):e0289155. doi: 10.1371/journal.pone.0289155 (PMC10374159; doi:10.1371/journal.pone.0289155)
Supplement: Attachment — Submitted filename: Response to Reviewers.docx [file pone.0289155.s011.docx]

|  | July, 04^th^ 2023 |
| --- | --- |

#### Sebastien KENMOE, PhD

##### Department of Microbiology and Parasitology

University of Buea

e mail : [sebastien.kenmoe@ubuea.cm](mailto:sebastien.kenmoe@ubuea.cm)

##### Tél: + (237) 674 05 95 26

**Submission Manuscript ID PONE-D-22-31497**

**Article title: Prevalence of the metabolic syndrome in African populations: a systematic review and meta-analysis.**

**The Editor-in-Chief**

**Plos One**

**Review Comments to the Author**

**Editor #:**

1. Please address to the quality assessment checklist in the abstract.

Authors: Thank you for this valuable feedback on our manuscript. We have now added the method for assessing risk of bias in the abstract.

2. Please replace PRISMA 2009 with PRISMA flowchart 2020, in the main manuscript, figure 1, and ref. list.

Authors: Thank you for this valuable feedback on our manuscript. Your comment has been incorporated, and the flowchart and figure 1 have been updated with the PRISMA 2020 reference.

3. It is better to summarize reasons of excluded full texts

Authors: Thank you for your valuable feedback. We agree with your suggestion and have now included in Figure 1.

4. In PRISMA checklist (Table S1) should be inserted just page number, not location or other data.

Authors: Thank you for your valuable feedback. Your suggestion had already been taken into account in S1 Table.
